# Supplementary material for: Cost-effectiveness of sleeping sickness elimination campaigns in five settings of the Democratic Republic of Congo
Source: Nat Commun. 2022 Feb 25;13:1051. doi: 10.1038/s41467-022-28598-w (PMC8881616; doi:10.1038/s41467-022-28598-w)
Supplement: Supplementary file 1 — Supplementary Information [file 41467_2022_28598_MOESM1_ESM.pdf]

Cost-effectiveness of sleeping sickness elimination campaigns in five settings of the Democratic Republic of Congo

Contents

A Supplementary Methods **2**
- A.1 Locations 2
- A.2 Models 3
- A.3 Health outcomes denominated as disability-adjusted life-years (DALYs) 10
- A.4 Cost functions 12
- A.5 Cost-effectiveness analysis 24
- A.6 Sensitivity Analysis 25

B Supplementary Tables and Figures **26**
- B.1 Decision model of the current analysis 27
- B.2 Supplementary outcome summaries 28
- B.3 Supplementary cost summaries 30
- B.4 Supplementary cost-effectiveness acceptability curves 37
- B.5 Sensitivity analysis: time-horizon and time discounting 39
- B.6 Sensitivity analysis: vector control 43

C Supplementary Discussion **46**
- C.1 Incremental cost-effectiveness ratios, dominance, and weak dominance 46

D Supplementary Note 1: Glossary of Technical Terms **47**

E Supplementary Note 2: PRIME Criteria **48**

F Supplementary Note 3: CHEERS Checklist **49**

G Supplementary Note 4: Parameter Glossary **52**
- G.1 Principles for parameterization 54
- G.2 Organization of parameters 54
- G.3 Summary of health outcome parameters 56
- G.4 Summary of cost parameters 57
- G.5 Screening parameters 58
- G.6 Treatment parameters 61
- G.7 Life-years lost (DALY) parameters 69
- G.8 Vector control parameters 71
- G.9 Screening cost parameters 71
- G.10 Treatment cost parameters 75
- G.11 Vector control cost parameters 77

H Supplementary References **78**

A Supplementary Methods

A.1 Locations

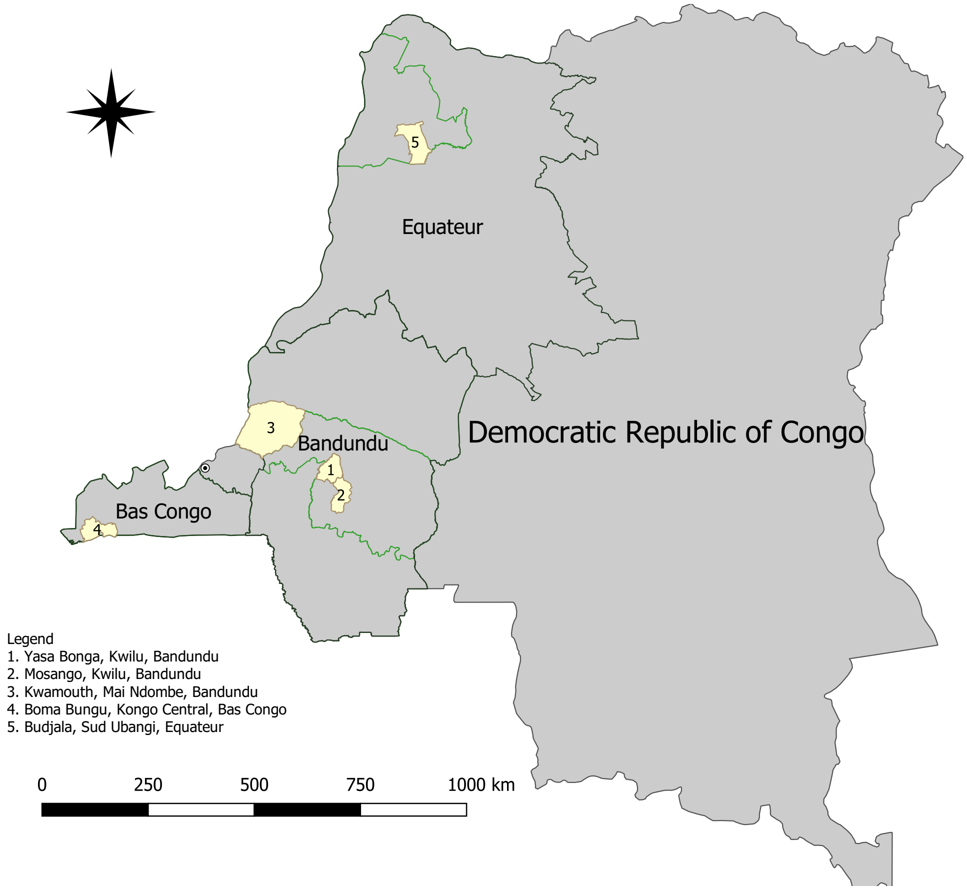

Supplementary Figure 1: Locations of the specific health zones considered in this study are shown in yellow. New provincial boundaries are denoted in green, and former provincial designations are denoted in black.

A.2 Models

A.2.1 Transmission model

The epidemiological model [1, 2], as described in the methods of the main manuscript, assumes that only low-risk people participate in AS, and high-risk individuals who become diseased are only detected in PS. The proportion of the population considered low-risk is estimated during the fitting process [1] based on historic data (2000–2016). Previous modelling exercises have demonstrated that this is a reasonable modelling assumption based on observed case trends in some parts of DRC and Chad [3, 4], and this type of heterogeneity is necessary to provide a good match to data.

Trends in case reporting and infection levels from 2018, the final year of the period for which data were available, were simulated under a range of alternative intervention strategies, to which cost-effectiveness analysis could then be applied.

Case detection as well as the time lived with disease for cases that were never detected was simulated assuming that the observed prevalence is a combination of simulated prevalence and diagnostic accuracy. Our model assumes that suspects in AS activities are first screened by the card agglutination test for trypanosomiasis (CATT), although sometimes this could be done by a rapid diagnostic test (RDT) instead. Suspects that are screened in fixed facilities (passive screening) are first tested with RDTs. CATT-positive or RDT-positive suspects then have blood drawn for microscopic examination searching for trypanosomes. For patients who qualify for fexinidazole treatment according to WHO guidelines, no further testing is assumed, and for other suspects a lumbar puncture to stage their disease would be performed to determine whether pentamidine or NECT treatment would be administered.

Elimination of transmission (EOT) is assumed when the underlying transmission (not detected cases) falls below 1 new infection per year. Such a proxy threshold is necessary when using deterministic transmission models which represent average dynamics and asymptote to zero rather than a stochastic model including chance events which can hit zero. The metric of interest in this paper is regional EOT (where activities must remain to prevent re-establishment) rather than eradication because we are not treating the issue of importation of cases [5]. This is also related to the WHO's gHAT goal for 2030 which is global EOT to humans [6, 7].

Uncertainty. Uncertainty in fitted epidemiological parameter values is incorporated by drawing from their joint posterior distributions as estimated by adaptive MCMC [1, 2]. Most of the fitted parameters correspond to features of the model that can be expected to vary between health zones: including the proportion of the population at low-risk vs high-risk of infection, the rate at which tsetse bite humans rather than other non-human animals, and the reporting rate by passive screening. By contrast, parameters that correspond to features conserved throughout all health zones are assumed to be fixed at available literature estimates: e.g. human mortality rate, tsetse biting rate, and stage 1 to stage 2 disease progression in humans. As well as the variability among joint posterior samples of the fitted parameters; some uncertainty around the parameters that we assume to be fixed, measurement and recording error, and other variation from unknown sources is partially included by sampling detected cases and deaths around the deterministic model outputs from over-dispersed beta-binomial distributions. To characterise the uncertainty throughout the model, we used 10,000 draws from the posterior of the transmission model. Trace plots of the year of elimination of transmission (EOT) and probability of reactive screening (RAS) are displayed in Supplementary Figures 2-3, indicating that the features of the distribution are robustly estimated with 10,000 samples.

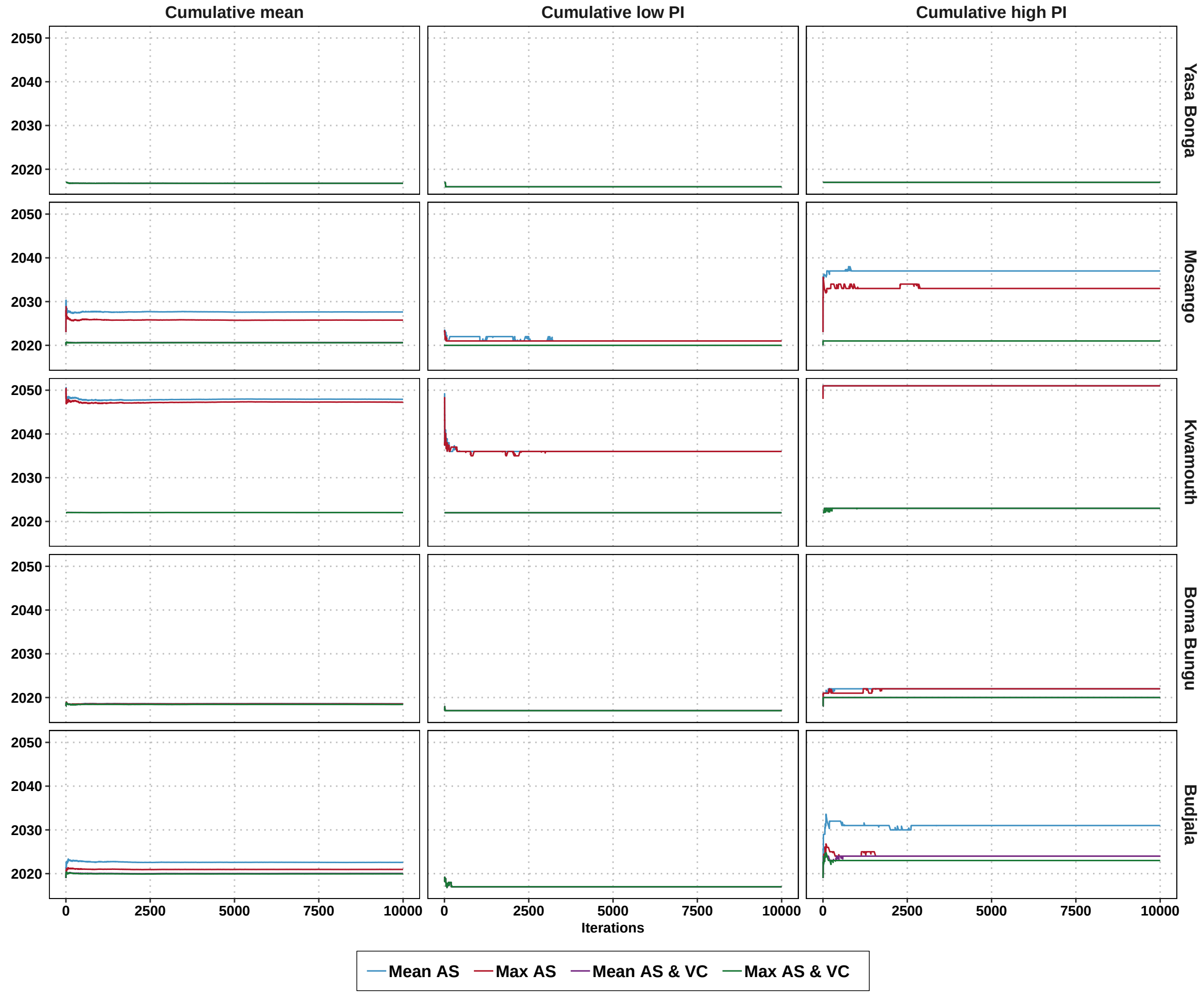

Supplementary Figure 2: Trace plot of year of elimination of transmission (EOT). On the left column, trace plots of the posterior samples of the year of transmission are shown by health zones (rows). In columns 2-4, the cumulative mean, the lower, and upper predictive intervals (PI, corresponding to the 2.5th and 97.th percentile) are calculated, with increasing numbers of iterations (x-axis) used to calculate the statistic of interest of the sample.

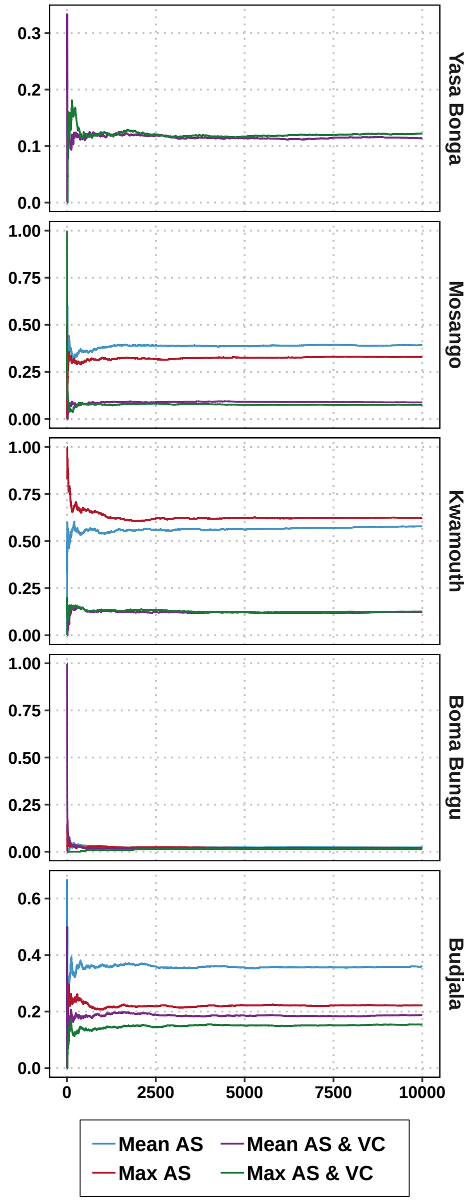

Supplementary Figure 3: Trace plot of the probability of reactive screening (RS). The cumulative probability of reactive screening is calculated by taking increasing numbers of iterations (x-axis) to iteratively calculate the probability of reactive screening – discarding the iterations where active screening never ceases.

A.2.2 Strategy components

Active screening. Each active screening team consists of 8–9 individuals that travel by truck to a village [8–10]. A team has a screening capacity of about 60,000 people per year although there is formidable variance in the literature (see Supplementary Note 4: Parameter Glossary, section G.5.6).

Coverage. Coverage of active screening is simulated at "mean" levels and "max" levels. "Mean AS" indicates that the number of people screened is the mean of the AS for the period 2014–2018 whereas under a "Max AS" strategy, the number of people screened is the maximum screened in any single year between 2000–2018. The simulations for the decision analysis were started on 2020 (as no decision can now be made about activities in 2019). The actual screening numbers are applied in 2017 and 2018 and "Mean AS" is assumed for all strategies in 2019.

Activities. AS teams can perform initial screening tests with a CATT test or RDT. For those that are CATT- or RDT-positive, the team then examines lymph node glands and performs microscopy exams, consisting of a blood sample taken and examined via mAECT (mini-anion exchange centrifugation technique).

Traditionally, the stage of disease is then determined by microscopy examination of the cerebro-spinal fluid, which is extracted via lumbar puncture for cases in which trypanosomes are present in the blood. Depending on whether trypanosomes are present in the cerebro-spinal fluid (stage 2) or not (stage 1) patients are referred to the appropriate health center or health district hospital for treatment. In the context of fexinidazole treatment, we do not expect that lumbar punctures will be performed by the active screening team, but rather that patients are referred to a health center that will determine eligibility for fexinidazole treatment. However, fexinidazole has only recently been approved for use and how eligibility takes place and lumbar punctures are administered is to be seen.

We note here that while mini-mobile teams are in operation in selected health zones of DRC, their effectiveness at linking cases to care is still under investigation. The potential implications of strategies that employ mini-mobile teams is therefore beyond the scope of this paper.

Reactive screening. Reactive screening (RS) is equivalent to AS, but it occurs after a case has been identified in a health zone where AS had ceased after a three-year period of no cases.

Passive screening. PS, or detection in fixed health facilities is assumed to take place with RDT diagnostic tests used for initial serological screening, followed by confirmatory testing (possibly at another facility with greater diagnostic capacity). The epidemiological model assumes that people are more likely to be detected through the passive system in stage 2 disease rather than stage 1, although in recent years the time until detection has decreased in some locations. For each health zone we have parameterised the stage 1 and 2 detection rates based on historic data for the health zone and the former province it is contained in [1]. In former Bandundu and Bas Congo provinces, we have simulated improvements in PS as there was a strong signal for this in the data. Unlike AS which is assumed to only identify low-risk cases, we assume any person with symptoms may self-present for passive detection regardless of risk status.

Coverage. Coverage of PS was assumed to depend on the number of health centres that can perform a serological confirmation of gHAT. We assumed that health zones were served by the same number of centres that were operational for serological confirmation in 2014, according to a publication by Simarro *et al* [11] (Table 1). Based on unpublished data by colleagues at the Institute of Tropical Medicine-Antwerp, our assumption was that between 0.003 and 0.01 of the population could be screened for gHAT by each clinic, most of which are expected to be gHAT-negative (see Supplementary Note 4: Parameter Glossary, section G.5.2). We note that the Report of the third WHO stakeholder's meeting on gHAT elimination, indicated that 437,402 patients were screened in 514 centres in 2017. We do not know the source population in the areas where the population was screened, but it indicates that approximately 851 patients were screened per centre, in line with our estimates (see Supplementary Methods, section A.4.2 and Supplementary Note 4: Parameter Glossary, section G.5.2).

Activities. A selection of fixed health centres can perform screening for gHAT in each health zone. Generally, these centres perform screening with RDT tests rather than CATT because CATT kits (of fifty tests) spoil within a week if unused. For suspects that are RDT-positive, the health worker examines lymph node glands and performs microscopy exams, consisting of a blood sample taken and examined via mAECT (mini-anion exchange centrifugation technique).

Traditionally, the stage of disease is determined by microscopy examination of the cerebro-spinal fluid, which is extracted via lumbar puncture for cases in which trypanosomes are present in the blood. Depending on whether trypanosomes are present in the cerebro-spinal fluid (stage 2) or not (stage 1) patients are referred to the appropriate health center or health district hospital for treatment. In the context of fexinidazole treatment, we do not expect that lumbar punctures will be performed at the health centre where diagnosis exists, but rather at the treatment centre where eligibility for fexinidazole treatment is determined.

We note here that while there are now some health centres that can administer RDT tests and refer patients to

health district hospitals (the referral centres) for confirmation and staging (when necessary) we did not evaluate the impact of additional health centres because the overall impact on disease detection is still under investigation [12].

Vector control. In all places, deployments are assumed to occur twice per year, based on the frequency at which it is expected that targets are knocked down or washed away [13–15]. However, other characteristics are more location-specific, which we describe below.

First, the locations where activities are ongoing (Yasa Bonga) or are planned (Kwamouth).

- Yasa Bonga: Our default assumption is that vector control must be deployed along 210 kilometers with 60 targets per kilometer with 90% tsetse reduction, which is the reduction that was observed in field studies in this health zone [15].
- Kwamouth: Our default assumption is that vector control must be deployed along 432 kilometers with 20 targets per kilometer, which is planned in this health zone (personal communication: Prof. Steve Torr). We have assumed 80% tsetse reduction, which is conservative compared to what has been observed in other regions [13, 15].

Second, in the locations where activities are neither ongoing nor planned:

- In Mosango, Boma Bungu, and Budjala: Our default assumption is that 100 kilometers of riverbank must be protected with uniform distribution of 40 targets per kilometer. Our rationale for choosing 100 km is that the incidence in these areas is rather low, and so a similar location to use as an example is Arua, in Northwestern Uganda, where only about 77 km of riverbank were protected with 20 targets per kilometer [13].

Sensitivity analyses are described in Supplemental Methods, section A.6.

A.2.3 Treatment model

We combined the case detections from the dynamic model with a model of treatment outcomes. The model was conceived as a probability tree (Figure 1). The probability of any of the outcomes at the tips of the tree (convalescence, death, treatment failure & S2, rescue treatment with NECT – all with and without serious adverse events) were the result of a series of conditional probabilities along the steps of treatment, serious adverse events, and diagnosis of treatment failure.

We assumed the treatment algorithm based on the WHO interim recommendations of 2019 [16].

- **Step 1, Group A. Patients without clinical symptoms of severe gHAT.** These patients might be eligible for fexinidazole treatment according to the following criteria:
  - **Patients under 6 years old or under 20 kg of weight.** There patients are considered ineligible for fexinidazole treatment. We assumed that all patients over 6 years old were also over 20 kg. We made this assumption because little good data on the patient characteristics are available and it made little different in the costs and effects of the current analysis. See Step 2, Group A.
  - **Patients over 6 years old and over 20 kg of weight.** There patients are considered eligible for fexinidazole treatment. See Step 2, Group B. The recommendations also stipulated that a doctor ought to be certain of adherence in the part of the patient in order to prescribe fexinidazole on an outpatient basis, but we have assumed that this is not an issue for simplicity and because it doesn't make a substantial difference in the costs or effects of this particular analysis.
- **Step 1, Group B. Patients with clinical symptoms of severe HAT.** Patients whose clinical assessment would be consistent with severe gHAT (see Annex 1 of the WHO Interim guidelines [16]) would undergo a lumbar puncture to determine the amount of WBC in the cerebro-spinal fluid. If they were to find fewer than 100 WBC per microliter, then the patient would be referred to fexinidazole treatment. In our model, we assume that none of the stage 1 patients will show more than 100 WBC/μL of CSF as this is a feature of stage 2 disease, and we have assumed that some proportion of stage 2 patients will be in late-stage disease (see Supplementary Tables 1-2). Patients with less than 100 WBC/μL of CSF would be considered for fexinidazole eligibility based on age and weight, just as those patients in Step 1, Group A.
- **Step 2, Group A. Patients ineligible for fexinidazole treatment.** Patients who were either under 6 years old or under 20 kg in weight undergo a lumbar puncture to determine disease stage. In our model, we consider the cost of a lumbar puncture in these patients (see Supplementary Table-13) but the outcome of the lumbar puncture (stage 1 or stage 2) is determined by the transmission model.
  - The WHO recommendations stipulate the following criteria: if there are under 5 leukocytes (or white blood

cells, WBC) per microliter of cerebrospinal fluid (CSF) and no trypanosoma in the CSF, then the patient undergoes pentamidine treatment on an inpatient basis. If there are more than 5 leukocytes (or white blood cells, WBC) per microliter (μL) then the patient undergoes NECT treatment on an inpatient basis. However, the stage of disease is a critical output of the transmission model (described in Supplementary Methods, section A.2.1) and therefore, the outcome of the lumbar puncture (stage 1 or stage 2) is determined by the transmission model. We consider the cost of a lumbar puncture in these patients (see Supplementary Table 13).

  – Pentamidine treatment would consist of intra-muscular infections for 7 days. We assumed all pentamidine treatment would have to be administered on an in-patient basis [16].

  – NECT (Nifurtimox-eflornithine combination therapy) is administered on an inpatient basis for 10 days. Nifurtimox is administered orally for 10 days, while eflornithine is administered intravenously for 7 days [16].

- **Step 2, Group B. Patients eligible for fexinidazole treatment.** We assumed that patients would be treated on an inpatient basis if they were over 6 years old and between 20 kg and 35 kg in weight, otherwise, they would be treated on an outpatient basis as directly observed therapy. We assumed that treatment would consist of 1800 mg of fexinidazole for four days and 1200 mg of fexinidazole for six additional days.

Uncertainty Uncertainty in the treatment and cost parameters is the variance of estimates in the literature (see Supplementary Note 4: Parameter Glossary, sections G.6 and G.10 for a list of all treatment effect and cost parameters respectively).

Patient Characteristic	Parameterization	Summary
Under 6 years old	Beta(152.5, 2427.9)	0.06 (0.05, 0.07)
Under 35 kg of weight	Beta(8.3, 359.6)	0.02 (<0.01, 0.04)
Late stage-2 disease	Beta(76.9, 44.9)	0.63 (0.54, 0.72)

Supplementary Table 1: Parameters for treatment eligibility.

Eligibility	Rationale	Summary
**Stage 1**		
Pentamidine	Under 6 years old (1)	0.06 (0.05, 0.07)
Fexinidazole-inpatient	Over 6 years old but under 35 kg of weight	0.02 (<0.01, 0.04)
Fexinidazole-outpatient	Over 6 years old and over 35 kg of weight	0.92 (0.90, 0.93)
**Stage 2**		
NECT	Under 6 years old or late-stage disease	0.65 (0.57, 0.73)
Fexinidazole-inpatient	Over 6 years old but under 35 kg of weight and early stage-2 disease	<0.01 (<0.01, 0.01)
Fexinidazole-outpatient	Over 6 years old, over 35 kg of weight, and early stage-2 disease	0.34 (0.26, 0.42)

[1] For simplicity, all patients over 6 years old were assumed to be over 20 kg in weight.

Supplementary Table 2: Eligibility for treatment. NECT: nifurtimox-eflornithine combination therapy.

Treatment	Outcomes	Estimate
**Stage 1**		
Pentamidine	Cured	0.05 (0.05, 0.06)
	Cured with SAEs	<0.01 (<0.01, <0.01)
	Rescue treatment	<0.01 (<0.01, <0.01)
	Death	<0.01 (<0.01, <0.01)
Fexinidazole - inpatient	Cured	0.02 (<0.01, 0.04)
	Cured with SAEs	<0.01 (<0.01, <0.01)
	Rescue treatment	<0.01 (<0.01, <0.01)
	Death	<0.01 (<0.01, <0.01)
Fexinidazole - outpatient	Cured	0.89 (0.87, 0.91)
	Cured with SAEs	0.01 (<0.01, 0.02)
	Rescue treatment	0.02 (<0.01, 0.03)
	Death	<0.01 (<0.01, <0.01)
**All treatments**	**Cured**	**0.97 (0.95, 0.98)**
	**Cured with SAEs**	**0.01 (<0.01, 0.03)**
	**Rescue treatment**	**0.02 (0.01, 0.03)**
	**Death**	**<0.01 (<0.01, <0.01)**
**Stage 2**		
NECT	Cured	0.56 (0.49, 0.64)
	Cured with SAEs	0.06 (0.04, 0.08)
	Rescue treatment	0.03 (0.01, 0.04)
	Death	<0.01 (<0.01, <0.01)
Fexinidazole - inpatient	Cured	<0.01 (<0.01, 0.01)
	Cured with SAEs	<0.01 (<0.01, <0.01)
	Rescue treatment	<0.01 (<0.01, <0.01)
	Death	<0.01 (<0.01, <0.01)
Fexinidazole - outpatient	Cured	0.33 (0.25, 0.41)
	Cured with SAEs	<0.01 (<0.01, <0.01)
	Rescue treatment	<0.01 (<0.01, 0.01)
	Death	<0.01 (<0.01, <0.01)
**All treatments**	**Cured**	**0.90 (0.88, 0.92)**
	**Cured with SAEs**	**0.07 (0.05, 0.09)**
	**Rescue treatment**	**0.03 (0.02, 0.05)**
	**Death**	**<0.01 (<0.01, <0.01)**

Supplementary Table 3: Treatments and outcomes distributions for stage 1 and 2 patients, calculated according to the probability tree in 1. SAE: severe adverse events.

A.3 Health outcomes denominated as disability-adjusted life-years (DALYs)

As per recommendations of the Bill and Melinda Gates Foundation's reference case and WHO's guidelines for the conduct of cost-effectiveness analyses, we defined the utility of gHAT interventions in terms of disability-adjusted life-years (DALYs) [17–19]. DALYs were discounted at a rate of 3% per year [17, 19]. We follow established conventions to calculate DALYs and evaluate the estimates in present-day terms (after applying discounting) [17, 19, 20].

A.3.1 Brief overview of disability-adjusted life-years

DALYs are defined as the sum of the years of life lost to disability (illness) (YLD) and the years of life lost by fatal cases (YLL) [18, 19]. QALYs, a metric related to DALYs averted, is not typically used in analysis of diseases in the developing settings.

$$\text{DALY}_{\text{strategy}} = \sum_i \text{YLL}_i + \text{YLD}_i \tag{1}$$

Three economic notions are integrated into these terms: time-discounting (with a yearly discount rate r), relative utility (via disability weights, which in the context of HAT must be conditional on the stage of disease), and and the average life-expectancy of the population in the analysis.

A.3.2 Years of life lost (YLL)

The years of life lost are the years that the individual i would have been expected to live less the age that the individual died in. We do not use the life-table method of accounting (which would take into account that the life-expectancy is actually conditional on the current age of the patient.) Our method is simple yet, we believe, sufficiently accurate because the average age of death of gHAT is as an adult, when life expectancy does not fluctuate appreciably. We do not adjust for the differences in life-expectancy by gender because this is not common practice, and the difference in DALYs averted would be even smaller after time-discounting is taken into account.

$$\text{YLL}_i^{\text{undiscounted}} := \text{life expectancy} - \text{age at death}_i \tag{2}$$

To calculate discounted values, we must calculate the discounted years of life lost in terms of the year in which the death occurs, and then adjust the years lost to present day terms (by discounting a second time):

$$\text{YLL}_i^{\text{discounted}} := \frac{1}{(1+r)^{(\text{year of death}_i - \text{present year})}} \sum_{k=1}^{\text{YLL}_i^{\text{undiscounted}}} \frac{1}{(1+r)^k} \tag{3}$$

For deaths that occur in the future, that means that we must discount future years of life lost to the individual at the time of death, and then we must apply the discounting rate a second time to value their death in present-day terms. For example, suppose the discounting rate is 3%, the individual loses 20 years of life at death in 2025, and the present day is 2014. In that case, that individual loses $20/(1+0.03)^{20} = 11.07$ in 2025, but then we value the years lost in present-day terms (in 2014) by applying a discount rate again: $11.07/(1+0.03)^{11} = 8$. So an individual whose life is spared from death in 2025 and lives from 2025–2045, only accrues 8 years of "DALYs averted" from the perspective of the decision-maker considering prevention options in 2014. If, on the contrary, another intervention saves a person's life in 2014 and the person is estimated to live 20 more years, that person accrues 11.07 years of "DALYs averted" from the perspective of the decision-maker considering prevention options in 2014. In this way, our calculation of DALYs averted shows a preferences for interventions that save lives in younger individuals and sooner rather than later.

We assumed that the mean age of death from gHAT is 26.6 years, according to the limited existing data on the age of infected patients (see Supplementary Note 4: Parameter Glossary, section G.6.4. We extracted life expectancy estimates from the World Bank's World Development Index [21]. We do not expect that this is a cause of major uncertainty so we have applied a fixed value. For information on the parameterisation of life-expectancy, see Supplementary Note 4: Parameter Glossary, section G.7.1.

A.3.3 Years of life lost to disability (YLD)

The years of life lost to disability are calculated as follows. It is important to note that disability and duration are conditioned on the stage for HAT.

$$\text{YLD}_i^{\text{undiscounted}} := \sum_{\text{stage}=1}^{2} \text{duration}_{\text{stage}} \times \text{disability weight}_{\text{stage}} \tag{4}$$

If we apply discounting, we must calculate the discounted years of life lost due to disability in terms of the year in which the individual experiences the disability:

$$\text{YLD}_i^{\text{discounted}} := \sum_{\text{illness years}_i} \frac{\text{YLD}_i^{\text{undiscounted}}}{(1+r)^{(\text{illness years}_i - \text{present year})}} \tag{5}$$

A disability weight, a factor between 0 (perfect health) and 1 (equivalent to death), is applied to years of life lost due to illness (but not to death) to reflect the severity of gHAT relative to other diseases and to death. These weights are calculated via studies that calculate weights for a compendium of common symptoms. We use the disability weights calculated and used by the Global Burden of Disease study [22], as detailed in Supplementary Note 4: Parameter Glossary, sections G.7.2-G.7.3.

A.4 Cost functions

The total costs can be characterised by the following expression. Each of the elements in this function, however, might have a functional form of its own, as described in the tables for active screening, passive surveillance, vector control and treatment.

$$\text{Total costs} = \sum_{i \in \text{all sub-categories}} \Big(U_i \times C_i \Big) \tag{6}$$

i is the cost sub-category. Where U is the unit of cost, which varies depending on the activity, such as people screened, teams deployed, fixed health centres outfitted with tests, etc. C is the cost per unit. All costs were denominated in 2018 US$.

A.4.1 Cost functions: active screening

The yearly costs of AS were calculated as a function of two groups of expenses: 1) overhead costs, and 2) the number of screening tests and confirmation tests that are used across all teams within the health zone. Because no synergies with other disease programs are believed to exist, we have employed a full costing method.

- Overhead costs: overhead costs are split between capital costs and recurrent costs to run an active screening team. AS teams serve a "coordination"– a subnational designation of the PNLTHA program that manages a set of health zones. Therefore, a health zone where fewer than 60,000 people are targeted for screening does not necessarily incur larger overhead costs for active screening than a health zone where the coverage is closer to a multiple of the yearly capacity of a team.
  - Capital costs consist of vehicles, medical equipment, energy (solar panels) and training (which occurs once every few years).
  - Recurrent costs consist of management and consumables that are spent on the team: fuel, staff time, etc.
- Costs that scale by population served:
  - CATT tests are scaled up according to the number of people that are screened per year, and a slight mark-up is included to account for wastage of CATT tests.
  - Confirmation tests are counted for all of those who are positive according to the CATT test, both the false positives (which are modelled according to the specificity of the test) and the true positives, which are the outputs of the dynamic model.
  - Lumbar punctures are not depicted as part of the AS costs, but are included as part of the treatment costs. Because many patients are eligible for fexinidazole treatment, which does not require lumbar punctures, we include lumbar puncture costs in the treatment portion of the analysis for those patients that are not eligible for fexinidazole. See Supplementary Methods, section A.4.4.
- For all costs, there is a PNLTHA mark-up to account for the central management at the national program headquarters. Snijders and colleagues [10] estimate that PNLTHA management equals approximately 15% of costs, and we have made the same assumption.

The parameters for AS are described in Supplementary Note 4: Parameter Glossary, section G.5 and the cost parameters are described in Supplementary Note 4: Parameter Glossary, section G.9.

Item	Units (U)	Cost (C)
Capital (annualized)	AS coverage per year × Population ÷ Capacity of one AS team	AS capital × (1+PNLTHA mark-up)
Recurrent expenses	AS coverage per year × Population ÷ Capacity of one AS team	AS recurrent × (1+PNLTHA mark-up)
CATT testing (See Note 1)	AS coverage per year × Population × (1+wastage factors for AS CATT administration)	CATT × (1+delivery mark-up) × (1+PNLTHA markup)
Confirmation (microscopy)	(1-CATT specificity) × (AS coverage per year × Population) + Cases S1 detected with AS + Cases S2 detected with AS	Microscopy × (1+PNLTHA markup)

[1] Ideally, CATT tests would be used for active screening and RDT tests would be used for passive screening because of the high wastage of CATT tests in the context of passive screening settings.

Supplementary Table 4: Active screening (AS): cost function. CATT: card agglutination test for trypanosomiasis.

Variable Name	Parameterization	Summary
Population	Fixed	See Table 1
AS coverage per year	Fixed	See Table 1
Capacity of one AS team per year	Normal(60,000, 10,000)	59,918 (40,446, 79,547)
CATT algorithm: wastage during AS	Beta(8, 92)	0.08 (0.04, 0.14)
CATT specificity	Beta(31, 2)	0.94 (0.84, 0.99)
AS capital costs (annualized)	Gamma(81, 115)	9,269 (7,322, 11,436)
AS recurrent costs (annual)	Gamma(63, 631)	39,935 (30,927, 50,321)
Cost of CATT test	Gamma(23, 0.02)	0.46 (0.29, 0.66)
Cost confirmation (microscopy)	Gamma(8.475, 1.27)	10.73 (4.80, 18.87)
Cost of delivery (markup)	Beta(45, 55)	0.45 (0.35, 0.55)
PNLTHA markup	Uniform(0.1, 0.2)	0.15 (0.10, 0.20)

Supplementary Table 5: Components of active screening costs. AS: active screening. CATT: card agglutination test for trypanosomiasis. PNLTHA: Programme National de Lutte contre la Trypanosomie Humaine Africaine.

The cost for each health zone per year, given the number of people screened per health zone, is therefore:

Supplementary Table 6: Cost breakdown for active screening activities. CATT: card agglutination test for trypanosomiasis.

Item	Units (U)	Cost per unit (C)	Cost per category
**Yasa Bonga, mean coverage**			
Capital (annualized)	2.18 (1.59, 3.13)	10,656 (8,376, 13,211)	23,169 (15,544, 34,958)
Recurrent expenses	2.18 (1.59, 3.13)	45,908 (35,231, 58,185)	99,818 (65,722, 151,683)
CATT testing	136,588 (130,945, 144,295)	0.53 (0.33, 0.76)	71,839 (44,798, 104,678)
Confirmation (microscopy)	7,636 (1,018, 19,961)	12.33 (5.51, 21.78)	94,093 (10,485, 286,739)
**Total**			**288,919 (181,210, 487,461)**
**Yasa Bonga, additional coverage in max AS**			
Capital (annualized)	1.30 (0.95, 1.87)	10,656 (8,376, 13,211)	13,820 (9,272, 20,852)
Recurrent expenses	1.30 (0.95, 1.87)	45,908 (35,231, 58,185)	59,541 (39,203, 90,478)
CATT testing	81,474 (78,108, 86,070)	0.53 (0.33, 0.76)	42,852 (26,722, 62,439)
Confirmation (microscopy)	4,555 (608, 11,907)	12.33 (5.51, 21.78)	56,125 (6,254, 171,037)
**Total**			**172,337 (108,090, 290,766)**
**Mosango, mean coverage**			
Capital (annualized)	0.73 (0.53, 1.05)	10,656 (8,376, 13,211)	7,789 (5,226, 11,752)
Recurrent expenses	0.73 (0.53, 1.05)	45,908 (35,231, 58,185)	33,558 (22,095, 50,995)
CATT testing	45,920 (44,023, 48,511)	0.53 (0.33, 0.76)	24,152 (15,061, 35,192)
Confirmation (microscopy)	2,567 (342, 6,711)	12.33 (5.51, 21.78)	31,633 (3,525, 96,399)
**Total**			**97,132 (60,922, 163,881)**
**Mosango, additional coverage in max AS**			
Capital (annualized)	0.56 (0.41, 0.80)	10,656 (8,376, 13,211)	5,956 (3,996, 8,987)
Recurrent expenses	0.56 (0.41, 0.80)	45,908 (35,231, 58,185)	25,662 (16,896, 38,996)
CATT testing	35,115 (33,664, 37,096)	0.53 (0.33, 0.76)	18,469 (11,517, 26,911)
Confirmation (microscopy)	1,963 (262, 5,132)	12.33 (5.51, 21.78)	24,190 (2,696, 73,717)
**Total**			**74,277 (46,587, 125,320)**
**Kwamouth, mean coverage**			
Capital (annualized)	1.08 (0.79, 1.55)	10,656 (8,376, 13,211)	11,519 (7,728, 17,380)
Recurrent expenses	1.08 (0.79, 1.55)	45,908 (35,231, 58,185)	49,628 (32,676, 75,415)
CATT testing	67,910 (65,104, 71,741)	0.53 (0.33, 0.76)	35,718 (22,273, 52,044)
Confirmation (microscopy)	3,797 (506, 9,924)	12.33 (5.51, 21.78)	46,782 (5,213, 142,563)
**Total**			**143,647 (90,096, 242,359)**
**Kwamouth, additional coverage in max AS**			
Capital (annualized)	0.47 (0.35, 0.68)	10,656 (8,376, 13,211)	5,040 (3,381, 7,604)

Supplementary Table 6: Cost breakdown for active screening (AS) activities *(continued)*

Item	Units (U)	Cost per unit (C)	Cost per category
Recurrent expenses	0.47 (0.35, 0.68)	45,908 (35,231, 58,185)	21,712 (14,296, 32,994)
CATT testing	29,711 (28,483, 31,387)	0.53 (0.33, 0.76)	15,626 (9,745, 22,769)
Confirmation (microscopy)	1,661 (222, 4,342)	12.33 (5.51, 21.78)	20,467 (2,281, 62,371)
**Total**			**62,845 (39,417, 106,032)**
**Boma Bungu, mean coverage**			
Capital (annualized)	0.11 (0.08, 0.15)	10,656 (8,376, 13,211)	1,134 (761, 1,710)
Recurrent expenses	0.11 (0.08, 0.15)	45,908 (35,231, 58,185)	4,884 (3,216, 7,422)
CATT testing	6,683 (6,407, 7,060)	0.53 (0.33, 0.76)	3,515 (2,192, 5,122)
Confirmation (microscopy)	374 (50, 977)	12.33 (5.51, 21.78)	4,604 (513, 14,030)
**Total**			**14,136 (8,866, 23,851)**
**Boma Bungu, additional coverage in max AS**			
Capital (annualized)	0.32 (0.24, 0.46)	10,656 (8,376, 13,211)	3,432 (2,303, 5,179)
Recurrent expenses	0.32 (0.24, 0.46)	45,908 (35,231, 58,185)	14,788 (9,736, 22,471)
CATT testing	20,235 (19,399, 21,377)	0.53 (0.33, 0.76)	10,643 (6,637, 15,507)
Confirmation (microscopy)	1,131 (151, 2,957)	12.33 (5.51, 21.78)	13,939 (1,553, 42,479)
**Total**			**42,802 (26,845, 72,215)**
**Budjala, mean coverage**			
Capital (annualized)	<0.01 (<0.01, 0.01)	10,656 (8,376, 13,211)	100 (67, 151)
Recurrent expenses	<0.01 (<0.01, 0.01)	45,908 (35,231, 58,185)	432 (284, 656)
CATT testing	591 (566, 624)	0.53 (0.33, 0.76)	311 (194, 453)
Confirmation (microscopy)	33 (4, 86)	12.33 (5.51, 21.78)	407 (45, 1,240)
**Total**			**1,249 (784, 2,108)**
**Budjala, additional coverage in max AS**			
Capital (annualized)	0.82 (0.60, 1.17)	10,656 (8,376, 13,211)	8,698 (5,835, 13,123)
Recurrent expenses	0.82 (0.60, 1.17)	45,908 (35,231, 58,185)	37,472 (24,672, 56,943)
CATT testing	51,276 (49,157, 54,169)	0.53 (0.33, 0.76)	26,969 (16,818, 39,296)
Confirmation (microscopy)	2,867 (382, 7,494)	12.33 (5.51, 21.78)	35,323 (3,936, 107,643)
**Total**			**108,461 (68,027, 182,995)**

A.4.2 Cost functions: passive screening (screening at fixed health posts)

The yearly costs of passive screening were calculated as a function of two groups of expenses: 1) overhead costs, and 2) the number of consultations and screening and confirmation tests that are done in a health zone.

- Overhead costs: overhead costs are split between capital costs and recurrent costs to equip a health center to perform serological confirmation for HAT.
  - Capital costs consist of medical equipment, energy (solar panels) and training (which occurs periodically every few years). These costs are scaled by the number of facilities that can perform serological confirmation.
  - Recurrent costs consist of management: health district and provincial management and supervision. Only one management 'unit' was assumed per health zone, irrespective of the number of health centers that are capable of screening.
- Costs that scale by population served:
  - RDT tests are scaled up according to the number of people that are screened per year, and a slight mark-up is included to account for wastage of tests.
  - Confirmation tests are counted for all of those who are positive according to the RDT test: both the false positives (which are modelled as a factor equal to the specificity of the test) and the true positives, which are the outputs of the dynamic model.
  - Lumbar punctures are not depicted as part of the passive surveillance diagnosis costs, but are included as part of the treatment costs. Because many patients are eligible for fexinidazole treatment, which does not require lumbar punctures, we include lumbar puncture costs in the treatment portion of the analysis for those patients that are not eligible for fexinidazole. See Supplemental Methods, section A.4.4.
- For all costs, there is a PNLTHA mark-up to account for the central management at the national program headquarters. Snijders *et al* [10] estimate that PNLTHA management equals approximately 15% of costs, and we have made the same assumption.
  The parameters for AS are described in Supplementary Note 4: Parameter Glossary, section G.5 and the cost parameters are described in Supplementary Note 4: Parameter Glossary, section G.9.

**Item**	**Units (U)**	**Cost (C)**
Capital (annualized)	Number of facilities capable of PS within the health zone	Capital costs (clinic) × (1+PNLTHA mark-up)
District management	Per district	District management cost × (1+PNLTHA mark-up)
Consultation	PS coverage per year per clinic × Clinics in the health zone × Population	Consultation cost × (1+PNLTHA markup)
RDT testing (See Note 1)	PS coverage per year per clinic × Clinics in the health zone × Population	RDT × (1+delivery mark-up) × (1+PNLTHA markup)
Confirmation (microscopy)	(1-RDT specificity) × (PS coverage per year per clinic × Clinics in the health zone × Population) + Cases S1 detected with PS + Cases S2 detected with PS	Microscopy × (1+PNLTHA markup)

[1] Ideally, CATT tests would be used for active screening and RDT tests would be used for passive screening because of the high wastage of CATT tests in the context of passive screening settings.

Supplementary Table 7: Passive screening (PS): cost function. RDT: rapid diagnostic test. CATT: card agglutination test for trypanosomiasis. PNLTHA: Programme National de Lutte contre la Trypanosomie Humaine. S1 & S2: stage 1 and 2 disease, respectively.

Variable Name	Parameterization	Summary
Population	Fixed	See Table 1
PS facilities	Fixed	See Table 1
PS coverage per facility	Beta(36, 7927)	0.007 (0.004, 0.010)
RDT algorithm: wastage during PS	Beta(1, 99)	0.01 (<0.01, 0.04)
RDT specificity	Beta(226, 31)	0.88 (0.84, 0.92)
Capital costs (annualized)	Gamma(8.475, 210)	1,768 (793, 3,112)
Recurrent costs (yearly)	Gamma(8.475, 986)	8,334 (3,723, 14,869)
Cost of consultation	Gamma(1.37, 3.33)	2.34 (1.41, 3.28)
Cost of RDT	Gamma(8.475, 0.19)	1.61 (0.72, 2.88)
Cost confirmation (microscopy)	Gamma(8.475, 1.27)	10.73 (4.80, 18.87)
Cost of delivery markup	Beta(45, 55)	0.45 (0.35, 0.55)
PNLTHA markup	Uniform(0.1, 0.2)	0.15 (0.10, 0.20)

Supplementary Table 8: Components of passive screening (PS) costs. RDT: rapid diagnostic tests. PNLTHA: Programme National de Lutte contre la Trypanosomie Humaine Africaine.

The cost for each health zone per year, given the number of people screened per health zone and the number of health centres available for PS, is shown in table 9.

Item	Units (U)	Cost per unit (C)	Cost per category
**Yasa Bonga**			
Capital (annualized)	4	2,033 (909, 3,590)	8,131 (3,635, 14,359)
District management	1	9,580 (4,261, 17,063)	9,580 (4,261, 17,063)
Consultation	5,893 (3,210, 9,310)	2.69 (1.62, 3.80)	15,850 (6,904, 29,000)
RDTs	5,893 (3,210, 9,310)	2.68 (1.21, 4.82)	15,824 (5,712, 33,225)
Confirmation (microscopy)	711 (355, 1,225)	12.33 (5.51, 21.78)	8,768 (2,954, 19,035)
**Total**			**58,153 (35,668, 90,214)**
**Mosango**			
Capital (annualized)	1	2,033 (909, 3,590)	2,033 (909, 3,590)
District management	1	9,580 (4,261, 17,063)	9,580 (4,261, 17,063)
Consultation	830 (452, 1,312)	2.69 (1.62, 3.80)	2,233 (973, 4,086)
RDTs	830 (452, 1,312)	2.68 (1.21, 4.82)	2,230 (805, 4,682)
Confirmation (microscopy)	100 (50, 173)	12.33 (5.51, 21.78)	1,235 (416, 2,682)
**Total**			**17,311 (10,855, 25,836)**
**Kwamouth**			
Capital (annualized)	5	2,033 (909, 3,590)	10,164 (4,544, 17,949)
District management	1	9,580 (4,261, 17,063)	9,580 (4,261, 17,063)
Consultation	4,349 (2,369, 6,871)	2.69 (1.62, 3.80)	11,698 (5,095, 21,402)
RDTs	4,349 (2,369, 6,871)	2.68 (1.21, 4.82)	11,678 (4,216, 24,521)
Confirmation (microscopy)	525 (262, 904)	12.33 (5.51, 21.78)	6,471 (2,180, 14,048)
**Total**			**49,590 (31,425, 74,572)**
**Boma Bungu**			
Capital (annualized)	2	2,033 (909, 3,590)	4,066 (1,818, 7,180)
District management	1	9,580 (4,261, 17,063)	9,580 (4,261, 17,063)
Consultation	1,141 (622, 1,803)	2.69 (1.62, 3.80)	3,070 (1,337, 5,617)
RDTs	1,141 (622, 1,803)	2.68 (1.21, 4.82)	3,065 (1,106, 6,435)
Confirmation (microscopy)	138 (69, 237)	12.33 (5.51, 21.78)	1,698 (572, 3,687)
**Total**			**21,478 (13,939, 31,301)**
**Budjala**			
Capital (annualized)	2	2,033 (909, 3,590)	4,066 (1,818, 7,180)
District management	1	9,580 (4,261, 17,063)	9,580 (4,261, 17,063)
Consultation	1,772 (965, 2,799)	2.69 (1.62, 3.80)	4,765 (2,076, 8,718)
RDTs	1,772 (965, 2,799)	2.68 (1.21, 4.82)	4,757 (1,717, 9,988)
Confirmation (microscopy)	214 (107, 368)	12.33 (5.51, 21.78)	2,636 (888, 5,722)
**Total**			**25,803 (16,759, 37,823)**

Supplementary Table 9: Cost breakdown for passive screening activities. RDT: rapid diagnostic tests. PNLTHA: Programme National de Lutte contre la Trypanosomie Humaine Africaine.

A.4.3 Cost functions: vector control

The costs of vector control (VC) were calculated as a function of two features: 1) the extent of the rivers where VC is deployed, and 2) the number of targets per kilometre of river where the targets were deployed. Full cost methods were used for the available cost data and that method was thus employed here. Because no synergies with other disease programs are believed to exist, we believe that a full costing method is appropriate.

The costs of entomological surveys (tsetse monitoring), sensitisation of the population (information campaigns), and district management were assumed to scale in relation to the extent of the health zone that where VC is deployed. The materials and labor-time related to target deployment was then considered to scale in relation to the amount of targets deployed.

There are thus far three recent instances of vector control described in the scientific literature: Arua, Uganda [13, 23]; Mandoul, Chad [4]; and Yasa Bonga, DRC [15]. In Arua, 1551 targets were deployed at 20 targets per kilometre, for a total of 77.5 kilometres of riverbank. In Mandoul, 2708 targets were deployed at 60 targets per kilometre, for a total of 45 kilometres of riverbank. In Yasa Bonga, 22,622 targets were deployed over a year throughout two deployments at 55 targets per kilometre (personal communication with colleagues from the Liverpool School of Tropical Medicine (LSTM), June 2020). The standard operating procedures for VC from LSTM say that 40 targets should be deployed per linear kilometre, although the particular landscape of the river ought to be taken into account [14].

The extent of the riverbank that is necessary to cover for adequate coverage is determined by the case reports of the previous five years. Because both the extent of riverbank and the density of targets is unknown unless a thorough analysis of the health zone is performed, we have chosen to show extensive sensitivity analyses of the cost-effectiveness of different strategies:

- In Yasa Bonga, we only performed analyses of the costs of 210 km of river bank and 55 targets per kilometre.
- In Kwamouth, the plan is to deploy targets along 432 kilometres of riverbank at approximately 20 targets per kilometre. Although operations have begun, the full deployment has not been performed yet. To speak to concerns of uncertainty, we performed the analyses with 40 targets per kilometre, to understand the resource implications if more ambitious VC operations are necessary to reach a certain goal for tsetse density.

The full extent of our sensitivity analyses are described in Supplemental Methods, section A.6, and the results are shown in Supplementary Tables and Figures, section B.6.

The parameters for VC are described in Supplementary Note 4: Parameter Glossary, section G.8 and the VC cost parameters are described in Supplementary Note 4: Parameter Glossary, section G.11.

Item	Units (U)	Cost (C)
Entomological surveys, sensitization and management	Kilometers of river covered	Cost for entomological surveys, sensitisation and district management per kilometer × (1+PNLTHA markup)
Target deployment	Kilometers of river covered × Targets per kilometer × Number of deployments per year	Cost for target deployment per target × (1+PNLTHA markup)

Supplementary Table 10: Vector Control (VC): cost function. PNLTHA: Programme National de Lutte contre la Trypanosomie Humaine.

Variable Name	Parameterization	Summary
Linear km	Fixed (sensitivity analyses)	100 or 210 (see note 1)
Units per km	Fixed (sensitivity analyses)	20 or 40 (see note 2)
Deployments per year	Fixed	2
Cost for entomological surveys, sensitisation and district management per km	Gamma(8.475, 14.17)	120.60 (53.70, 213.63)
Cost per target deployment per target	Gamma(8.475, 0.54)	4.55 (2.02, 8.04)
PNLTHA markup	Uniform(0.1, 0.2)	0.15 (0.10, 0.20)

[1] Due to Kwamouth's large size and two transmission hotspots, VC is deployed in 432 km of river.
[2] In Yasa Bonga, the current units per km are 60, above the recommendations.

Supplementary Table 11: Components of vector control costs. PNLTHA: Programme National de Lutte contre la Trypanosomie Humaine Africaine.

Per year, the simulated costs according to the above formulation and parameters result in the following estimates.

Item	Units (U)	Cost (C)
**Yasa Bonga**		
Entomological surveys, sensitization and management	210 km	29,119 (13,023, 51,750)
Target deployment	25,200 targets	131,835 (58,625, 233,769)
**Total**		**160,954 (85,049, 265,011)**
**Kwamouth (planned): 432 km, 20 targets per kilometer**		
Entomological surveys, sensitization and management	432 km	59,902 (26,789, 106,456)
Target deployment	17,280 targets	90,401 (40,200, 160,299)
**Total**		**150,303 (87,033, 232,730)**
**Kwamouth (sensitivity analysis): 432 km, 40 targets per kilometer**		
Entomological surveys, sensitization and management	432 km	59,902 (26,789, 106,456)
Target deployment	34,560 targets	180,802 (80,400, 320,597)
**Total**		**240,704 (131,663, 386,342)**
**Other health zones (default analysis): 100 km, 40 targets per kilometer**		
Entomological surveys, sensitization and management	100 km	13,866 (6,201, 24,643)
Target deployment	8000 targets	41,852 (18,611, 74,212)
**Total**		**55,719 (30,478, 89,431)**
**Other health zones (sensitivity analysis 1): 100 km, 20 targets per kilometer**		
Entomological surveys, sensitization and management	100 km	13,866 (6,201, 24,643)
Target deployment	4000 targets	20,926 (9,306, 37,106)
**Total**		**34,792 (20,147, 53,873)**
**Other health zones (sensitivity analysis 2): 210 km, 20 targets per kilometer**		
Entomological surveys, sensitization and management	210 km	29,119 (13,023, 51,750)
Target deployment	8400 targets	43,945 (19,542, 77,923)
**Total**		**73,064 (42,308, 113,133)**
**Other health zones (sensitivity analysis 3): 210 km, 40 targets per kilometer**		
Entomological surveys, sensitization and management	210 km	29,119 (13,023, 51,750)
Target deployment	16,800 targets	87,890 (39,083, 155,846)
**Total**		**117,009 (64,003, 187,805)**

Supplementary Table 12: Cost breakdown for vector control activities

A.4.4 Cost functions: treatment

Item	Units (U)	Cost (C)
Doctor's consult	All patients	Outpatient consult × (1+PNLTHA markup)
Staging cases (supplies and time); patients ineligible for fexinidazole treatment (see Notes 1-2).	Patients in both stages of disease ineligible for fexinidazole treatment.	Lumbar puncture cost × (1+PNLTHA markup)
Pentamidine (see Notes 1-3).	Cases of stage 1 disease detected with AS or PS × proportion of patients ineligible for fexinidazole.	Pentamidine × (1+delivery mark-up) × (1+PNLTHA markup)
Inpatient care for stage 1 with pentamidine (see Notes 1-3).	Cases of stage 1 disease detected with AS or PS × proportion of patients ineligible for fexinidazole × length of treatment for pentamidine.	Inpatient cost per day × (1+PNLTHA markup)
NECT (see Notes 1-3).	Cases of stage 2 disease detected with AS or PS × proportion of patients ineligible for fexinidazole.	NECT × (1+delivery mark-up) × (1+PNLTHA markup)
Inpatient care for stage 2 with NECT (see Notes 1-3).	(Cases stage 2 detected with AS or PS) × proportion of patients ineligible for fexinidazole × length of treatment for NECT.	Inpatient cost per day × (1+PNLTHA markup)
Fexinidazole (see Notes 1-2).	Patients in both stages of disease eligible for fexinidazole treatment.	Fexinidazole × (1+delivery mark-up) × (1+PNLTHA markup)
Inpatient care for either stage 1 or 2 with fexinidazole (see Notes 1-3)	Patients eligible for fexinidazole on an inpatient basis × length of treatment for fexinidazole.	Inpatient cost per day × (1+PNLTHA markup)
Outpatient care for either stage 1 or 2 with fexinidazole (see Notes 1-4).	Patients eligible for outpatient treatment.	Outpatient consult × (1+PNLTHA markup)
Treatment for severe adverse events	Patients under each treatment who experience severe adverse events	(Outpatient consult + Inpatient cost per day × length of treatment for severe adverse events) × (1+PNLTHA markup)

[1] See WHO treatment recommendations for eligibility for fexinidazole. While some patients are eligible for fexinidazole (over 6 years of age and below the severe threshold of disease) the recommendations stipulate inpatient care for some patients due to low weight.

[2] The proportion of patients eligible for fexinidazole was determined as follows: (1-proportion of patients under 6 years of age) × (1-proportion of patients with signs of late stage 2 disease).

[3] The proportion of patients who had fexinidazole treatment on an inpatient basis was determined by multiplying the equation in note 2 with the proportion of patients who were over 35 kg of weight. The rest had either pentamidine or NECT depending on whether they had stage 1 or 2 disease, respectively.

[4] Fexinidazole is currently recommended on an outpatient basis as a directly-observed therapy, so we have imputed a cost for the daily administration by a village health worker. For simplicity, we have given this the same value as a regular outpatient visit since it constitutes a small portion of all costs.

Supplementary Table 13: Treatment: cost function. PNLTHA: Programme National de Lutte contre la Trypanosomie Humaine. NECT: nifurtimox-eflornithine combination therapy.

We show here the components of the costs per case treated depending on the stage and the treatment. The parameters for the above table are available in Supplementary Note 4: Parameter Glossary, section G.10 and eligibility distributions are described in Supplementary Table 2.

Variable.Name	Parameterization	Summary
Staging cost	Gamma(2.42, 3.66)	8.83 (1.41, 22.81)
PNLTHA mark-up	Uniform(0.1, 0.2)	0.15 (0.10, 0.20)
Duration of hospital stay for NECT treatment in days	Fixed	10
Duration of hospital stay fexinidazole in days	Fixed	10
Duration of SAE in days	Gamma (1.219, 2.377)	2.90 (0.13, 9.67)
Pr. of SAE - pentamidine	Beta(1,499)	0.0026 (0.0002, 0.0083)
Pr. of SAE - NECT	Beta(11.6,226.4)	0.10 (0.07, 0.13)
Pr. of SAE - fexinidazole	Beta(3,261)	0.01 (<0.01, 0.03)
Outpatient consultation cost	Gamma(1.37,3.33)	2.34 (1.41, 3.28)
Hospital cost per day	Gamma(5.81,0.24)	1.39 (0.49, 2.72)
Course of pentamidine cost	Fixed	54
Course of NECT cost	Fixed	460
Course of fexinidazole cost	Fixed	50
Delivery mark-up	Beta(45,55)	0.45 (0.35, 0.55)

Supplementary Table 14: Parameters for treatment costs. SAE: severe adverse events. NECT: nifurtimox-eflornithin combination therapy. Pr: probability.

	Pentamidine	NECT	Fexinidazole - inpatient	Fexinidazole - outpatient
Staging	10.15 (1.61, 26.22)	10.15 (1.61, 26.22)	0	0
Doctor's consult	21.52 (12.99, 30.36)	2.34 (1.41, 3.28)	2.34 (1.41, 3.28)	23.40 (14.14, 32.80)
Inpatient care	0	13.90 (4.92, 27.19)	13.90 (4.92, 27.19)	0
Medicine	78.31 (73.17, 83.56)	667.09 (623.26, 711.81)	72.51 (67.75, 77.37)	72.51 (67.75, 77.37)
Treatment for SAE	0.02 (<0.01, 0.06)	0.61 (0.19, 1.74)	0.07 (<0.01, 0.25)	0.07 (<0.01, 0.25)
**Total**	**100 (89, 117)**	**691 (645, 739)**	**87 (77, 101)**	**94 (83, 105)**

Supplementary Table 15: Cost per person for different gHAT treatments. PNLTHA markup is included. Because these are costs averaged over all patients and SAEs are rare, the average cost per patient for SAE is low.

A.5 Cost-effectiveness analysis

Cost-effectiveness analyses assess the technical efficiency of several strategies in a head-to-head comparison. Such analyses denominate the resource inputs in terms of dollars and the health benefits in terms of DALYs. The amount of resource inputs that ought to be allocated for a given health benefit (the "willingness-to-pay" (WTP)) is not pre-designated in the analysis; rather, the optimal strategies are shown for a range of resource inputs (WTP).

While the purpose of such efforts is to inform the use of budgets, an analysis that optimises budgets across disease priorities is often intractable (see the WHO CHOICE program [19, 24]), but the use of certain agreed upon conventions helps make analyses comparable across disease programs. To that end, this analysis abides by the *Consolidated Health Economic Evaluation Reporting Standards (CHEERS)* guidelines (see Supplementary Note 3: CHEERS Checklist).

The basic metric of cost-effectiveness is the incremental cost-effectiveness ratio (ICER, see Supplementary Note 1: Glossary of Technical Terms), which is the cost of averting a DALY of disease compared to the next-best strategy [25, 26]. In recent years, and as reflected in the CHEERS checklist, there has been a tendency to integrate different forms of uncertainty in decision analyses. To address parameter uncertainty, the "net monetary benefits (NMB) framework" was developed to integrate the outputs of probabilistic modelling into a metric that integrates the benefits of ICERs [26, 27].

It should be noted, that the net benefits framework yields two metrics: the probability that a strategy is optimal (in terms of cost-effectiveness), and whether the strategy yields the highest expected net benefit. While the strategy with the highest probability of being optimal is often the strategy with the highest mean NMB, it is not always the case. This situations arises when key parameters are simulated from asymmetric distributions to characterize their uncertainty. This paradox evokes a discussion in the literature as to whether decision-makers are risk-averse or utility-maximizing, and it is beyond the scope of this discussion to engage in that debate [28], but it is mentioned here as some results may reflect this paradox (see Supplementary Figures 7-8).

Decision model used. The decision model used is in the form of a decision tree, although it contains both time-dependent disease transmission nodes and single-time point disease treatment nodes (Supplementary Figure 4). The incidence of cases is modelled via an SEIRS model (briefly described in Supplementary Methods, section A.2.1 but developed in [2]). Although untreated infection lasts for 2–3 years, the majority of disease burden is borne by fatal cases and the majority of the costs arises from detection, so a detailed Markov model of disease progression with respect to time was not necessary.

Costs included. It should be noted that cost-effectiveness analyses aim to quantify all economic costs in the perspective of the agent (the health system in our analysis) therefore, materials that are not directly purchased for the purpose of the named strategies but that are kept from other medical use are also quantified. A separate analysis by Sutherland *et al* [29] examined the financial as well as the economic impacts of gHAT strategies.

Because some of the strategies proposed in this paper have not been put into place, a model-based estimate of the costs has been employed in order to project the size of those costs, detailed in Supplementary Methods, section A.4.

Time horizon and discounting. Since the purpose of the analysis is to assess the resource implications of EOT, which may require large investments in the short-term in return for long-term benefits, we have chosen the 20-year horizon as a default in the analysis. A ten-year horizon essentially discounts every year at 3% for ten years and at 100% thereafter, implying that long-term benefits are of no consequence to the decision-maker, but we do not believe this is the case in the context of gHAT elimination. We have provided select metrics in 10-year and 30-year horizons here in the supplement and in the companion website (see Supplementary Tables and Figures, section B.5) and in the companion website (`https://hatmepp.warwick.ac.uk/5HZCEA/v2/`). We have not chosen to provide time-horizons of longer than 30 years out of concern for our capacity to project robust costs and benefits this far into the future.

A.6 Sensitivity Analysis

Vector control. While the default level of tsetse population reduction used in this paper (80%) appears realistic and possibly conservative [4, 13, 15, 30], the operations necessary to achieve a certain level of effectiveness for VC are unclear. Therefore, we performed three way sensitivity analysis of VC effectiveness, vector extent, and vector target density:

- VC is assumed to decrease the population of tsetse in the first year of deployment by 80% in the baseline analysis, but we examined outcomes and optimal choices assuming a decline of 60% and 90% in tsetse population. In Yasa Bonga, where a 90% decrease in the population of tsetse has been observed, we do not run this sensitivity analysis.
- The extent of the riverbank treated: for Mosango, Boma Bungu, and Budjala, where no VC activities are currently planned, we examined the cost-effectiveness of interventions if 210 km of riverbank had to be treated instead of the default assumption of 100 km.
- The density of targets deployed per kilometre of riverbank treated. We assumed that 20 targets per kilometre would be applied in Kwamouth (which is what is planned) and that 40 targets per kilometre would be necessary in Mosango, Boma Bungu, and Budjala. In sensitivity analyses, we tested the robustness of our decision analysis if the density of targets would have be doubled in Kwamouth or halved in Mosango, Boma Bungu, and Budjala.

We point out in the supplementary results (section B.6) any policy-related changes to decision-making that were notable during sensitivity analyses, but complete calculations of costs, effects, and cost-effectiveness with each configuration of assumptions are available in the companion website for the paper: https://hatmepp.warwick.ac.uk/5HZCEA/v2/.

Decision analysis conventions. Two further elements of the decision analysis were re-examined in supplemental analyses. First, while an investment horizon of 2020–2040 was chosen for the principle analysis; shorter (2020–2030) and longer (2020–2050) investment horizons are reported in the Supplementary Tables and Figures, section B.5. In order to examine any differences in optimal choice. Second, while the convention in the cost-effectiveness literature is to discount both costs and health benefits by 3% per year, we have also reported the results when no discounting is taken into account. Results of these analyses are available in the companion website for the paper: https://hatmepp.warwick.ac.uk/5HZCEA/v2/.

B Supplementary Tables and Figures

B.1 Decision model of the current analysis . . . 27
B.2 Supplementary outcome summaries . . . 28
B.3 Supplementary cost summaries . . . 30
B.3.1 Strategy costs per person, per case, and per area, 2020-2040 . . . 32
B.4 Supplementary cost-effectiveness acceptability curves . . . 37
B.5 Sensitivity analysis: time-horizon and time discounting . . . 39
B.6 Sensitivity analysis: vector control . . . 43

B.1 Decision model of the current analysis

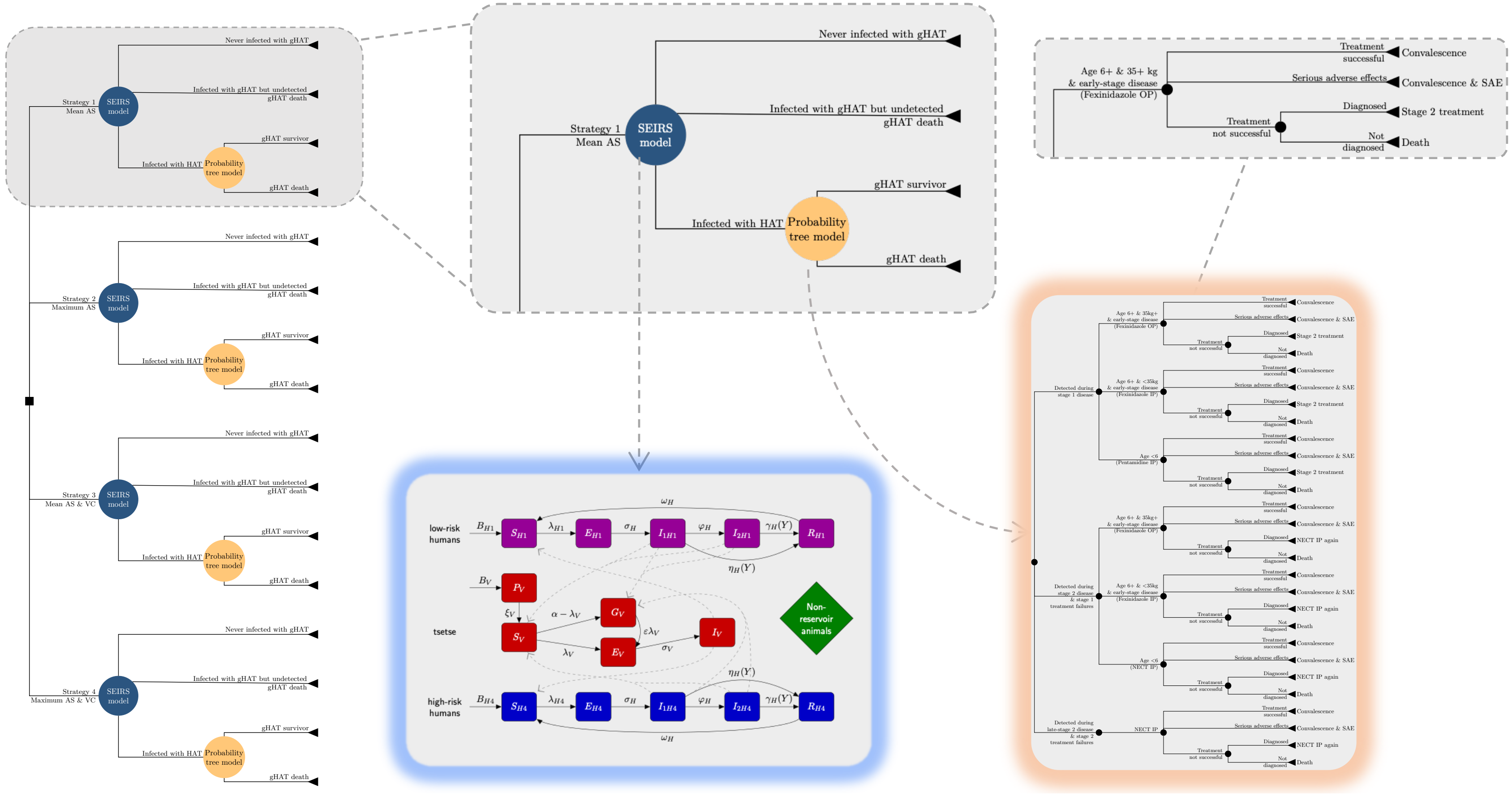

Supplementary Figure 4: Decision model of the current analysis. The relationship between the dynamic (susceptible-exposed-infected-recovered-susceptible, SEIRS) model and the probability tree model of disease treatment outcomes is shown here. The SEIRS model is described briefly in Supplementary Methods, section A.2.1, and the treatment model is shown in Figure 1 and described in Supplementary Methods, section A.2.3. Fitting of transmission model parameters and simulation of the model are described for the whole of DRC in other publications [1, 2]. Parameters denoted with (Y) are year-specific parameters, used to explain the evolution of transmission. Enhanced screening strategies increase $\eta_H(Y)$ (the rate of recovery from stage 1 disease) and $\lambda_H(Y)$ whereas vector control strategies increase background mortality of vectors (tsetse flies) in order to decrease the prevalence of infected tsetse flies (I_V) and therefore decrease the force of infection among humans.

B.2 Supplementary outcome summaries

	Cases detected (95% PI)	Deaths (95% PI)	DALYs (95% PI)	Total costs ($ millions) (95% PI)	Yearly costs ($) per capita (95% PI)
**Yasa Bonga**					
Mean AS & VC	5 (0, 23)	2 (0, 7)	62 (1, 238)	2.52 (1.14, 4.64)	1.03 (0.47, 1.90)
Max AS & VC	4 (0, 23)	2 (0, 7)	62 (1, 240)	3.26 (1.32, 6.15)	1.33 (0.54, 2.52)
**Mosango**					
Mean AS	22 (1, 74)	11 (1, 36)	393 (32, 1,224)	1.00 (0.46, 1.76)	0.73 (0.34, 1.28)
Max AS	21 (0, 91)	8 (0, 25)	270 (2, 888)	1.44 (0.59, 2.72)	1.04 (0.43, 1.98)
Mean AS & VC	9 (0, 41)	5 (0, 15)	168 (2, 510)	0.98 (0.47, 1.66)	0.71 (0.34, 1.21)
Max AS & VC	10 (0, 54)	4 (0, 12)	131 (1, 421)	1.29 (0.58, 2.27)	0.93 (0.42, 1.65)
**Kwamouth**					
Mean AS	379 (129, 790)	159 (36, 430)	5,546 (1,333, 14,860)	2.27 (1.60, 3.45)	1.58 (1.11, 2.39)
Max AS	379 (122, 809)	136 (32, 363)	4,773 (1,166, 12,527)	2.95 (2.05, 4.59)	2.05 (1.42, 3.18)
Mean AS & VC	116 (41, 234)	54 (17, 115)	1,880 (627, 4,011)	3.17 (2.06, 4.69)	2.20 (1.43, 3.25)
Max AS & VC	120 (38, 269)	49 (16, 104)	1,708 (561, 3,648)	3.70 (2.33, 5.59)	2.57 (1.62, 3.88)
**Boma Bungu**					
Mean AS	1 (0, 10)	0 (0, 4)	17 (0, 149)	0.27 (0.18, 0.40)	0.29 (0.19, 0.42)
Max AS	1 (0, 10)	0 (0, 3)	13 (0, 109)	0.38 (0.22, 0.66)	0.40 (0.23, 0.70)
Mean AS & VC	1 (0, 7)	0 (0, 3)	13 (0, 107)	0.41 (0.23, 0.69)	0.43 (0.25, 0.73)
Max AS & VC	1 (0, 8)	0 (0, 3)	11 (0, 97)	0.51 (0.27, 0.92)	0.54 (0.29, 0.98)
**Budjala**					
Mean AS	4 (0, 21)	5 (0, 16)	159 (0, 564)	0.29 (0.19, 0.42)	0.20 (0.13, 0.29)
Max AS	4 (0, 24)	2 (0, 8)	80 (0, 277)	0.66 (0.24, 1.25)	0.45 (0.16, 0.85)
Mean AS & VC	2 (0, 12)	2 (0, 8)	83 (0, 274)	0.43 (0.22, 0.74)	0.29 (0.15, 0.50)
Max AS & VC	3 (0, 19)	2 (0, 6)	56 (0, 208)	0.75 (0.24, 1.39)	0.51 (0.17, 0.94)

Supplementary Table 16: Summary of effects and costs 2020-2030. Two differences should be noted between these estimates and those used for decision analysis shown in table 23. First, these estimates are not discounted. Second due to asymmetric distributions, a naive difference in mean costs would not equal the mean differences in costs across simulations – the metric we used in decision analysis. Undetected cases are reflected in deaths, as very few deaths (<1 percent) originate from treated cases. Estimates shown are means and 95% prediction intervals (PI) of the cases, deaths, disability-adjusted life-years (DALYs), and costs across iterations of the dynamic transmission model.

	Cases detected (95% PI)	Deaths (95% PI)	DALYs (95% PI)	Total costs ($ millions) (95% PI)	Yearly costs ($) per capita (95% PI)
**Yasa Bonga**					
Mean AS & VC	5 (0, 23)	2 (0, 7)	62 (1, 240)	3.69 (2.10, 5.99)	0.54 (0.31, 0.87)
Max AS & VC	4 (0, 23)	2 (0, 7)	62 (1, 242)	4.42 (2.31, 7.49)	0.64 (0.34, 1.09)
**Mosango**					
Mean AS	23 (1, 79)	12 (1, 42)	428 (32, 1,431)	1.44 (0.76, 2.54)	0.37 (0.20, 0.66)
Max AS	22 (0, 92)	8 (0, 28)	282 (2, 987)	1.86 (0.91, 3.42)	0.48 (0.24, 0.88)
Mean AS & VC	9 (0, 41)	5 (0, 15)	169 (2, 510)	1.32 (0.77, 2.06)	0.34 (0.20, 0.53)
Max AS & VC	10 (0, 54)	4 (0, 12)	131 (1, 421)	1.63 (0.90, 2.65)	0.42 (0.23, 0.68)
**Kwamouth**					
Mean AS	511 (144, 1,230)	228 (41, 709)	7,946 (1,514, 24,538)	5.68 (3.58, 8.92)	1.40 (0.88, 2.20)
Max AS	490 (137, 1,158)	188 (36, 566)	6,575 (1,310, 19,499)	7.20 (4.35, 11.78)	1.77 (1.07, 2.90)
Mean AS & VC	116 (41, 235)	54 (18, 116)	1,890 (628, 4,026)	4.28 (2.88, 6.49)	1.05 (0.71, 1.60)
Max AS & VC	120 (38, 270)	49 (16, 105)	1,718 (562, 3,660)	4.83 (3.16, 7.57)	1.19 (0.78, 1.86)
**Boma Bungu**					
Mean AS	1 (0, 10)	0 (0, 4)	17 (0, 149)	0.70 (0.46, 1.02)	0.26 (0.17, 0.38)
Max AS	1 (0, 10)	0 (0, 3)	13 (0, 109)	0.81 (0.52, 1.20)	0.30 (0.20, 0.45)
Mean AS & VC	1 (0, 7)	0 (0, 3)	13 (0, 107)	0.84 (0.54, 1.23)	0.32 (0.20, 0.46)
Max AS & VC	1 (0, 8)	0 (0, 3)	11 (0, 97)	0.94 (0.58, 1.42)	0.35 (0.22, 0.53)
**Budjala**					
Mean AS	4 (0, 22)	5 (0, 18)	163 (0, 601)	0.81 (0.53, 1.18)	0.20 (0.13, 0.28)
Max AS	4 (0, 24)	2 (0, 8)	80 (0, 277)	1.17 (0.66, 1.86)	0.28 (0.16, 0.45)
Mean AS & VC	2 (0, 12)	2 (0, 8)	83 (0, 274)	0.95 (0.59, 1.41)	0.23 (0.14, 0.34)
Max AS & VC	3 (0, 19)	2 (0, 6)	56 (0, 208)	1.26 (0.67, 1.99)	0.31 (0.16, 0.48)

Supplementary Table 17: Summary of effects and costs 2020-2050. Two differences should be noted between these estimates and those used for decision analysis shown in table 24. First, these estimates are not discounted. Second due to asymmetric distributions, a naive difference in mean costs would not equal the mean differences in costs across simulations – the metric we used in decision analysis. Undetected cases are reflected in deaths, as very few deaths (<1 percent) originate from treated cases. Estimates shown are means and 95% prediction intervals (PI) of the cases, deaths, disability-adjusted life-years (DALYs), and costs across iterations of the dynamic transmission model.

B.3 Supplementary cost summaries

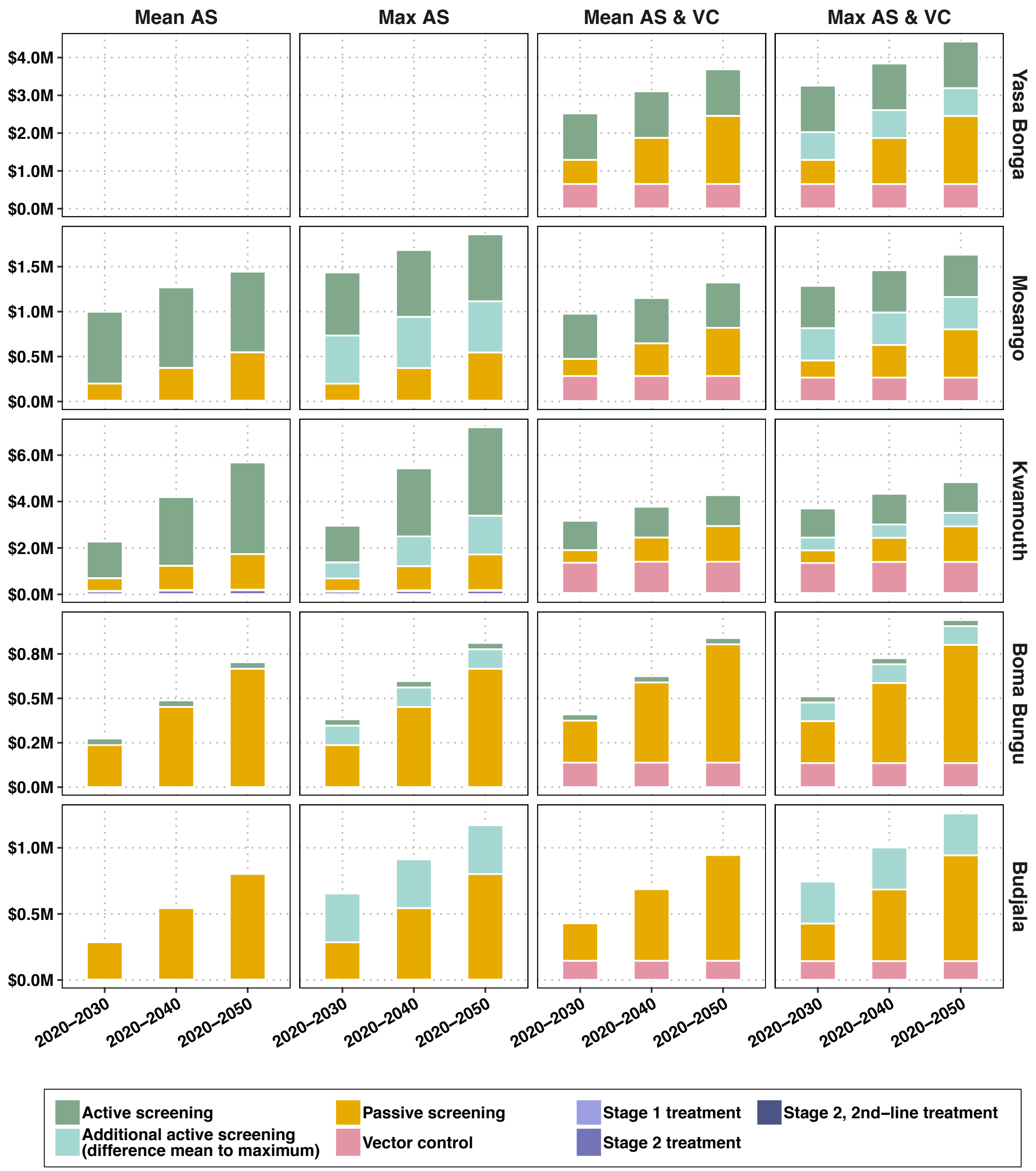

Supplementary Figure 5: Components of cumulative costs across three time horizons, by strategy and location. Costs are not discounted.

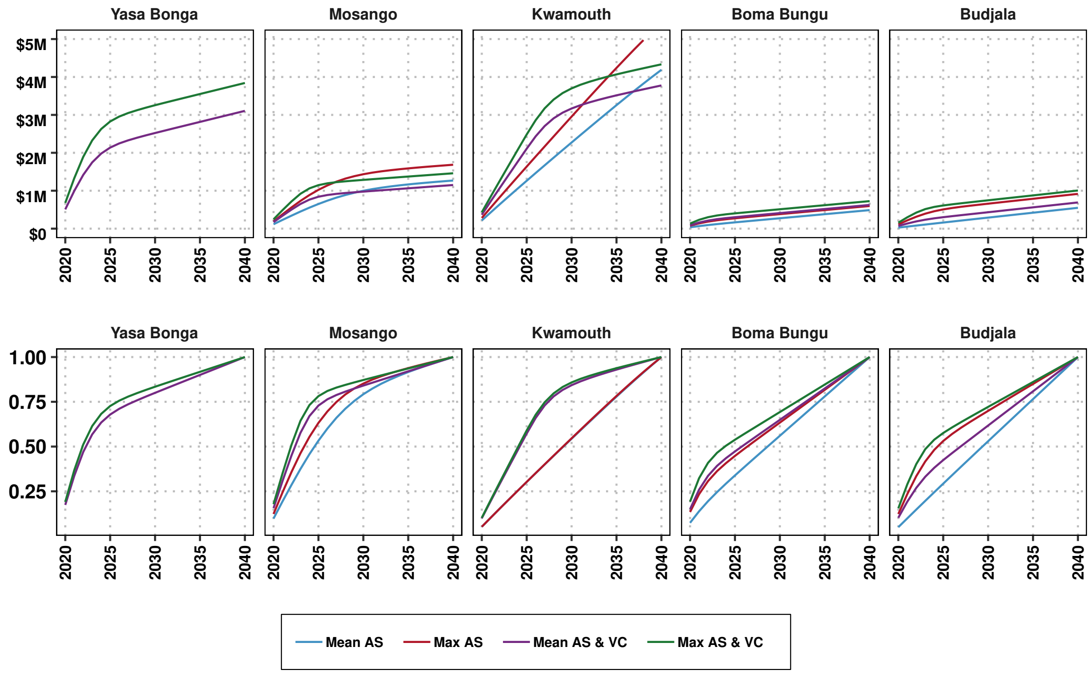

Supplementary Figure 6: Cumulative costs for each strategy through time, by health zone (top row) and the percent of the total cost spent by each year (bottom). Costs are not discounted.

B.3.1 Strategy costs per person, per case, and per area, 2020-2040

	Mean AS & VC	Max AS & VC
**Active screening**		
Total cases found	0 (0, 0)	0 (0, 0)
People screened	538,327 (126,007, 1,008,056)	860,932 (201,251, 1,610,008)
Stage 1 cases found	0 (0, 0)	0 (0, 0)
Stage 2 cases found	0 (0, 0)	0 (0, 0)
Total costs	1,233,844 (325,683, 2,744,540)	1,971,127 (499,872, 4,364,954)
Cost per person screened	2.29 (1.44, 3.87)	2.29 (1.44, 3.87)
Cost per capita	5.56 (1.47, 12.37)	8.88 (2.25, 19.67)
Cost per case detected	1,232,131 (319,224, 2,741,385)	1,970,133 (493,072, 4,364,954)
Pr. zero detections via AS	>0.99	>0.99
**Passive screening**		
Total cases found	5 (0, 23)	4 (0, 23)
People screened	123,751 (67,406, 195,510)	123,751 (67,406, 195,510)
Cases stage 1 found	0 (0, 1)	0 (0, 1)
Cases stage 2 found	4 (0, 23)	4 (0, 22)
Total costs	1,221,210 (749,019, 1,894,498)	1,221,210 (749,019, 1,894,498)
Cost per person screened	9.91 (7.08, 14.06)	9.91 (7.08, 14.06)
Cost per capita	5.50 (3.38, 8.54)	5.50 (3.38, 8.54)
Cost per case detected	713,580 (48,480, 1,747,968)	720,392 (48,120, 1,765,962)
Pr. zero detections via PS	0.34	0.34
**Vector control**		
Total costs	648,662 (151,591, 1,503,502)	646,984 (147,933, 1,479,354)
Cost per area	249 (58, 577)	248 (57, 568)
Cost per capita	2.92 (0.68, 6.78)	2.92 (0.67, 6.67)

Supplementary Table 18: Detailed summary of active screening, passive screening, and vector control in Yasa Bonga for the period of 2020-2040. Costs are not discounted.

	Mean AS	Max AS	Mean AS & VC	Max AS & VC
**Active screening**				
Total cases found	10 (0, 53)	13 (0, 76)	4 (0, 32)	6 (0, 48)
People screened	390,825 (127,128, 720,392)	572,093 (224,280, 1,046,640)	219,694 (84,752, 339,008)	362,878 (149,520, 598,080)
Stage 1 cases found	7 (0, 35)	9 (0, 52)	3 (0, 20)	4 (0, 32)
Stage 2 cases found	4 (0, 20)	4 (0, 26)	2 (0, 13)	2 (0, 19)
Total costs	895,966 (279,880, 1,919,511)	1,313,464 (400,973, 2,871,685)	504,360 (185,375, 1,018,169)	831,432 (293,111, 1,668,199)
Cost per person screened	2.29 (1.44, 3.87)	2.29 (1.44, 3.87)	2.29 (1.44, 3.87)	2.29 (1.44, 3.87)
Cost per capita	7.16 (2.24, 15.35)	10.50 (3.21, 22.96)	4.03 (1.48, 8.14)	6.65 (2.34, 13.34)
Cost per case detected	350,428 (18,207, 1,233,222)	539,729 (17,791, 1,867,479)	333,907 (15,141, 885,973)	524,994 (17,058, 1,418,912)
Pr. zero detections via AS	0.30	0.33	0.52	0.52
**Passive screening**				
Total cases found	13 (0, 42)	9 (0, 31)	5 (0, 20)	4 (0, 17)
People screened	17,437 (9,498, 27,548)	17,437 (9,498, 27,548)	17,437 (9,498, 27,548)	17,437 (9,498, 27,548)
Cases stage 1 found	2 (0, 10)	2 (0, 8)	1 (0, 4)	1 (0, 3)
Cases stage 2 found	10 (0, 34)	7 (0, 25)	4 (0, 18)	3 (0, 15)
Total costs	363,532 (227,948, 542,553)	363,532 (227,948, 542,553)	363,532 (227,948, 542,553)	363,532 (227,948, 542,553)
Cost per person screened	20.77 (12.79, 37.23)	20.77 (12.79, 37.23)	20.77 (12.79, 37.23)	20.77 (12.79, 37.23)
Cost per capita	2.91 (1.82, 4.34)	2.91 (1.82, 4.34)	2.91 (1.82, 4.34)	2.91 (1.82, 4.34)
Cost per case detected	77,306 (7,879, 406,682)	116,479 (10,355, 456,496)	171,730 (16,009, 486,859)	202,667 (19,613, 504,582)
Pr. zero detections via PS	0.04	0.09	0.18	0.26
**Vector control**				
Total costs	0 (0, 0)	0 (0, 0)	278,549 (79,794, 559,701)	261,416 (76,490, 526,134)
Cost per area	NA	NA	104 (30, 209)	98 (29, 197)
Cost per capita	NA	NA	2.23 (0.64, 4.47)	2.09 (0.61, 4.21)

Supplementary Table 19: Detailed summary of active screening, passive screening, and vector control in Mosango for the period of 2020-2040. Costs are not discounted.

	Mean AS	Max AS	Mean AS & VC	Max AS & VC
**Active screening**				
Total cases found	189 (30, 479)	222 (33, 580)	46 (2, 143)	56 (2, 188)
People screened	1,300,535 (1,072,377, 1,324,701)	1,852,338 (1,450,880, 1,904,280)	584,830 (378,486, 946,215)	834,809 (544,080, 1,360,200)
Stage 1 cases found	127 (17, 354)	155 (20, 440)	28 (1, 93)	36 (1, 126)
Stage 2 cases found	62 (11, 150)	66 (11, 162)	18 (0, 61)	20 (0, 70)
Total costs	2,961,960 (1,824,236, 5,040,768)	4,217,655 (2,544,127, 7,191,397)	1,331,356 (681,211, 2,631,073)	1,899,505 (961,791, 3,752,968)
Cost per person screened	2.28 (1.43, 3.84)	2.28 (1.43, 3.84)	2.28 (1.43, 3.84)	2.28 (1.43, 3.84)
Cost per capita	22.61 (13.92, 38.47)	32.19 (19.42, 54.89)	10.16 (5.20, 20.08)	14.50 (7.34, 28.64)
Cost per case detected	25,802 (5,412, 93,864)	32,805 (6,515, 120,955)	88,491 (7,481, 611,920)	131,763 (8,189, 1,083,753)
Pr. zero detections via AS	<0.01	<0.01	<0.01	0.01
**Passive screening**				
Total cases found	287 (96, 635)	242 (80, 530)	70 (27, 131)	64 (23, 123)
People screened	91,330 (49,747, 144,288)	91,330 (49,747, 144,288)	91,330 (49,747, 144,288)	91,330 (49,747, 144,288)
Cases stage 1 found	87 (26, 195)	74 (23, 166)	14 (4, 30)	13 (3, 27)
Cases stage 2 found	200 (66, 457)	168 (55, 373)	56 (21, 110)	51 (18, 105)
Total costs	1,041,399 (659,928, 1,566,012)	1,041,399 (659,928, 1,566,012)	1,041,399 (659,928, 1,566,012)	1,041,399 (659,928, 1,566,012)
Cost per person screened	11.46 (8.04, 16.92)	11.46 (8.04, 16.92)	11.46 (8.04, 16.92)	11.46 (8.04, 16.92)
Cost per capita	7.95 (5.04, 11.95)	7.95 (5.04, 11.95)	7.95 (5.04, 11.95)	7.95 (5.04, 11.95)
Cost per case detected	4,613 (1,428, 11,616)	5,435 (1,745, 13,853)	17,588 (6,714, 40,881)	19,457 (7,236, 46,573)
Pr. zero detections via PS	<0.01	<0.01	<0.01	<0.01
**Vector control**				
Total costs	0 (0, 0)	0 (0, 0)	1,353,920 (660,706, 2,545,956)	1,343,034 (648,485, 2,538,634)
Cost per area	NA	NA	93 (45, 175)	92 (44, 174)
Cost per capita	NA	NA	10.33 (5.04, 19.43)	10.25 (4.95, 19.38)

Supplementary Table 20: Detailed summary of active screening, passive screening, and vector control in Kwamouth for the period of 2020-2040. Costs are not discounted.

	Mean AS	Max AS	Mean AS & VC	Max AS & VC
**Active screening**				
Total cases found	0 (0, 1)	0 (0, 4)	0 (0, 1)	0 (0, 3)
People screened	16,160 (6,142, 42,994)	63,263 (24,744, 148,464)	15,441 (6,142, 36,852)	60,727 (24,744, 148,464)
Stage 1 cases found	0 (0, 1)	0 (0, 2)	0 (0, 0)	0 (0, 1)
Stage 2 cases found	0 (0, 1)	0 (0, 2)	0 (0, 0)	0 (0, 1)
Total costs	37,176 (9,415, 106,860)	145,680 (37,888, 406,611)	35,536 (9,415, 94,364)	139,533 (37,924, 362,492)
Cost per person screened	2.30 (1.44, 3.88)	2.30 (1.44, 3.88)	2.30 (1.44, 3.88)	2.30 (1.44, 3.88)
Cost per capita	0.43 (0.11, 1.24)	1.69 (0.44, 4.73)	0.41 (0.11, 1.10)	1.62 (0.44, 4.22)
Cost per case detected	36,082 (9,395, 101,975)	135,510 (34,445, 371,329)	34,800 (9,336, 91,462)	132,012 (34,858, 345,606)
Pr. zero detections via AS	0.96	0.94	0.97	0.94
**Passive screening**				
Total cases found	1 (0, 9)	1 (0, 7)	1 (0, 7)	1 (0, 6)
People screened	23,968 (13,055, 37,866)	23,968 (13,055, 37,866)	23,968 (13,055, 37,866)	23,968 (13,055, 37,866)
Cases stage 1 found	0 (0, 4)	0 (0, 3)	0 (0, 3)	0 (0, 2)
Cases stage 2 found	1 (0, 5)	0 (0, 5)	0 (0, 5)	0 (0, 4)
Total costs	451,039 (292,720, 657,315)	451,039 (292,720, 657,315)	451,039 (292,720, 657,315)	451,039 (292,720, 657,315)
Cost per person screened	18.85 (12.09, 32.24)	18.85 (12.09, 32.24)	18.85 (12.09, 32.24)	18.85 (12.09, 32.24)
Cost per capita	5.25 (3.41, 7.65)	5.25 (3.41, 7.65)	5.25 (3.41, 7.65)	5.25 (3.41, 7.65)
Cost per case detected	397,682 (48,218, 644,710)	405,111 (59,589, 649,568)	404,892 (63,109, 649,492)	413,726 (74,397, 652,577)
Pr. zero detections via PS	0.75	0.78	0.77	0.80
**Vector control**				
Total costs	0 (0, 0)	0 (0, 0)	137,698 (34,163, 358,971)	134,888 (34,129, 347,038)
Cost per area	NA	NA	48 (12, 125)	47 (12, 121)
Cost per capita	NA	NA	1.60 (0.40, 4.18)	1.57 (0.40, 4.04)

Supplementary Table 21: Detailed summary of active screening, passive screening, and vector control in Boma Bungu for the period of 2020-2040. Costs are not discounted.

	Mean AS	Max AS	Mean AS & VC	Max AS & VC
**Active screening**				
Total cases found	0 (0, 1)	2 (0, 18)	0 (0, 0)	1 (0, 15)
People screened	2,331 (0, 5,973)	165,660 (0, 339,696)	1,677 (0, 3,801)	142,352 (0, 291,168)
Stage 1 cases found	0 (0, 0)	1 (0, 12)	0 (0, 0)	1 (0, 9)
Stage 2 cases found	0 (0, 0)	1 (0, 6)	0 (0, 0)	0 (0, 6)
Total costs	5,343 (0, 15,172)	373,576 (0, 943,892)	3,851 (0, 9,585)	321,393 (0, 779,730)
Cost per person screened	2.30 (1.44, 3.88)	2.26 (1.42, 3.81)	2.30 (1.45, 3.88)	2.26 (1.42, 3.81)
Cost per capita	0.04 (<0.01, 0.11)	2.80 (<0.01, 7.07)	0.03 (<0.01, 0.07)	2.41 (<0.01, 5.84)
Cost per case detected	5,326 (0, 15,152)	297,971 (0, 829,900)	3,848 (0, 9,571)	270,249 (0, 708,646)
Pr. zero detections via AS	0.97	0.79	0.98	0.82
**Passive screening**				
Total cases found	4 (0, 22)	2 (0, 12)	2 (0, 12)	1 (0, 10)
People screened	37,202 (20,264, 58,774)	37,202 (20,264, 58,774)	37,202 (20,264, 58,774)	37,202 (20,264, 58,774)
Cases stage 1 found	2 (0, 9)	1 (0, 5)	1 (0, 5)	0 (0, 4)
Cases stage 2 found	3 (0, 15)	1 (0, 8)	1 (0, 8)	1 (0, 7)
Total costs	541,863 (351,941, 794,279)	541,863 (351,941, 794,279)	541,863 (351,941, 794,279)	541,863 (351,941, 794,279)
Cost per person screened	14.63 (9.87, 23.37)	14.63 (9.87, 23.37)	14.63 (9.87, 23.37)	14.63 (9.87, 23.37)
Cost per capita	4.06 (2.64, 5.95)	4.06 (2.64, 5.95)	4.06 (2.64, 5.95)	4.06 (2.64, 5.95)
Cost per case detected	314,247 (23,299, 743,891)	399,535 (40,726, 765,310)	404,435 (41,593, 765,381)	440,375 (50,750, 775,746)
Pr. zero detections via PS	0.33	0.48	0.49	0.59
**Vector control**				
Total costs	0 (0, 0)	0 (0, 0)	143,999 (0, 414,413)	141,440 (0, 387,365)
Cost per area	NA	NA	33 (0, 94)	32 (0, 88)
Cost per capita	NA	NA	1.08 (<0.01, 3.11)	1.06 (<0.01, 2.90)

Supplementary Table 22: Detailed summary of active screening, passive screening, and vector control in Budjala for the period of 2020-2040. Costs are not discounted.

B.4 Supplementary cost-effectiveness acceptability curves

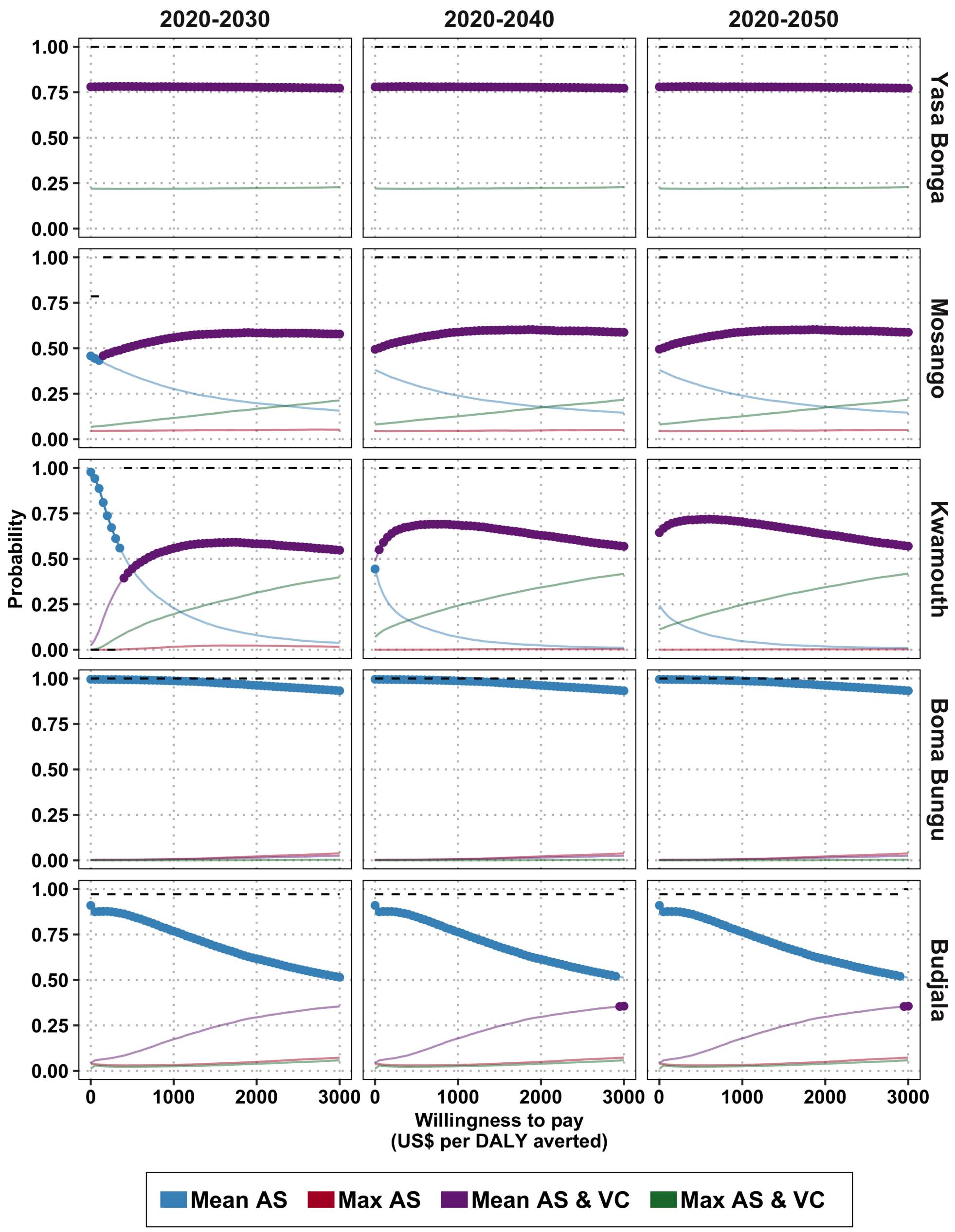

Supplementary Figure 7: Cost-effectiveness acceptability curves across different horizons assuming both costs and health effects are discounted. The probability that each intervention is optimal (cost-effective) is expressed by the solid lines in colors. The preferred intervention (in terms of mean net monetary benefit) is expressed by the dotted lines. The black dashed line shows probability that the preferred intervention reaches elimination of transmission (EOT) by 2030. For example, in Kwamouth, if one considers a 10-year horizon for investments, the strategy 'Mean AS' is cost effective at willingness-to-pay values below $500 per DALY averted, as shown by the dark blue dotted line, but that strategy has almost zero probability of reaching EOT by 2030, as shown by the black dashed line. If a decision-maker is willing to pay more than $500 per DALY averted, then the cost-effective strategy is 'Mean AS & VC', as displayed by the dark purple dotted line, and that strategy has an almost 100% probability of reaching EoT by 2030.

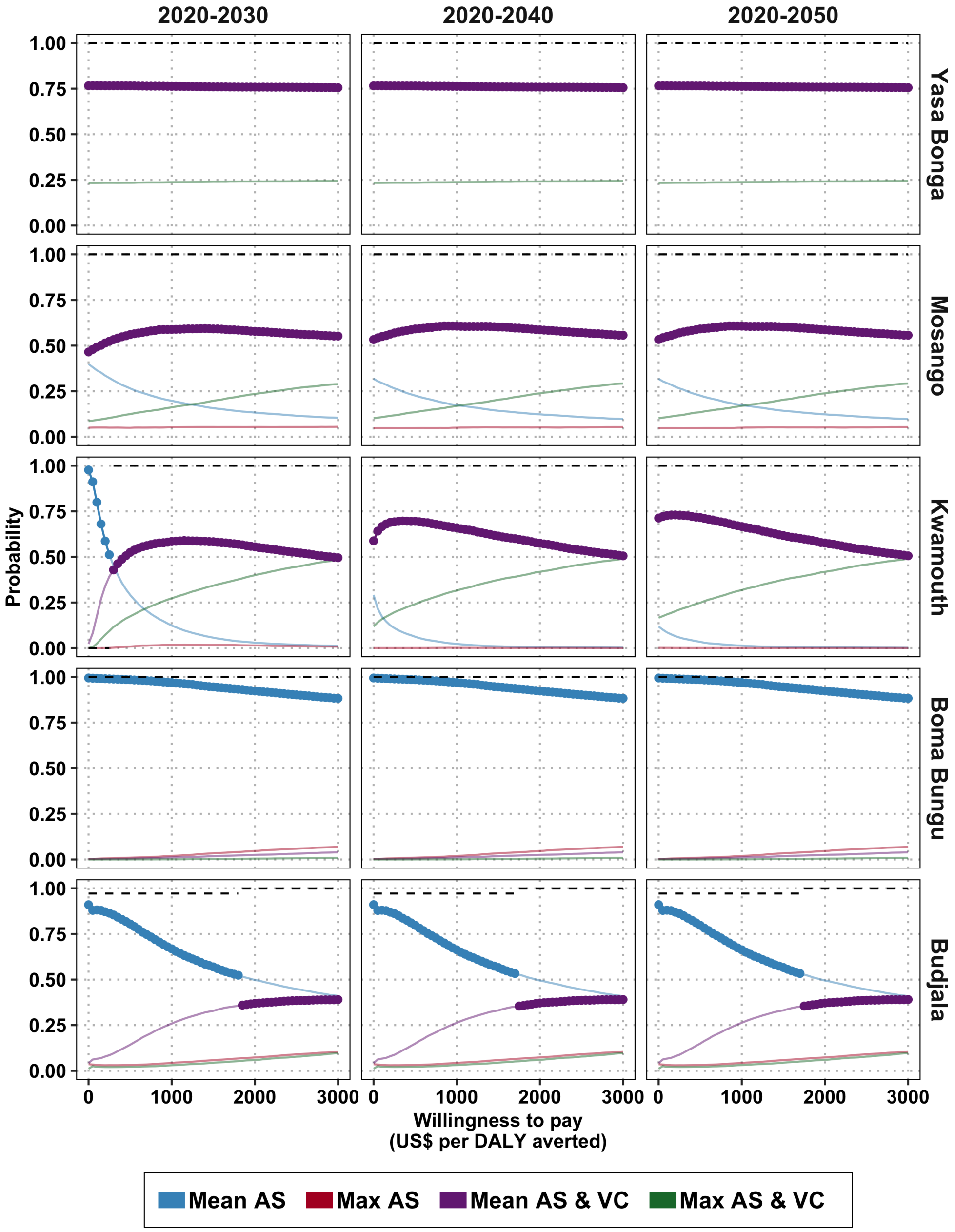

Supplementary Figure 8: Cost-effectiveness acceptability curves with no cost or health benefit discounts. The probability of cost-effectiveness is expressed by the solid lines. The preferred intervention (in terms of mean net monetary benefit) is expressed by the dotted dark line. The black dashed line shows probability that the preferred intervention reaches elimination of transmission (EoT) by 2030. For example, in Kwamouth, if one considers a 10-year horizon for investments, the strategy 'Mean AS' is cost effective at willingness-to-pay values below $500 per DALY averted, as shown by the dark blue dotted line, but that strategy has almost zero probability of reaching EoT by 2030, as shown by the black dashed line. If a decision-maker is willing to pay more than $500 per DALY averted, then the cost-effective strategy is 'Mean AS & VC', as displayed by the dark purple dotted line, and that strategy has an almost 100% probability of reaching EoT by 2030.

B.5 Sensitivity analysis: time-horizon and time discounting

	Cost-effectiveness analysis without uncertainty			Net benefit (uncertainty) analysis: Prob. that a strategy is optimal, (conditional on willingness-to-pay)				
	Cost difference vs comparator	DALYs averted vs comparator	ICER	$0 USD per DALY averted	$250 per DALY averted	$500 per DALY averted	$1,000 per DALY averted	Prob. EOT by 2030
**Yasa Bonga**								
Mean AS & VC	0	0	Min Cost	0.78	0.78	0.78	0.78	>0.99
Max AS & VC	670,123	0	2,156,689	0.22	0.22	0.22	0.22	>0.99
**Mosango**								
Mean AS	0	0	Min Cost	0.46	0.4	0.35	0.28	0.79
Max AS	390,631	71	Dominated	0.05	0.05	0.05	0.05	0.92
Mean AS & VC	15,695	127	123	0.43	0.48	0.51	0.56	>0.99
Max AS & VC	301,004	150	12,196	0.07	0.08	0.09	0.12	>0.99
**Kwamouth**								
Mean AS	0	0	Min Cost	0.98	0.67	0.44	0.23	<0.01
Max AS	574,743	440	Weakly Dominated	0	0	0	0.02	<0.01
Mean AS & VC	816,312	2,049	398	0.02	0.28	0.45	0.56	>0.99
Max AS & VC	1,276,239	2,156	4,263	0	0.05	0.11	0.2	>0.99
**Boma Bungu**								
Mean AS	0	0	Min Cost	1	0.99	0.99	0.99	>0.99
Max AS	101,573	3	40,380	0	0	0	0.01	>0.99
Mean AS & VC	127,904	2	Dominated	0	0	0	0.01	>0.99
Max AS & VC	223,244	4	93,437	0	0	0	0	>0.99
**Budjala**								
Mean AS	0	0	Min Cost	0.91	0.88	0.85	0.77	0.97
Max AS	335,514	45	Weakly Dominated	0.04	0.03	0.03	0.03	>0.99
Mean AS & VC	131,875	43	3,049	0.04	0.07	0.1	0.17	>0.99
Max AS & VC	423,315	60	17,581	0.01	0.02	0.02	0.03	>0.99

Supplementary Table 23: Summary of cost-effectiveness, assuming a time horizon of 2020-2030. Cost differences and differences in disability-adjusted life-years (DALYs) averted are relative to the comparator–first strategy listed for each location. Mean DALYs averted and mean cost differences are shown; these estimates are discounted at 3 percent per year in accordance with guidelines. The uncertainty analysis (columns 5-8) shows the probability that a strategy is cost-effective. Strategies highlighted in pink are optimal strategies: the strategies for which the mean net monetary benefit (NMB) is highest, equivalent to the information found in cost-effectiveness acceptability frontiers (CEAFs), which are shown Figures 7- 8. ICER: incremental cost-effectiveness ratio. For an extended discussion of these terms, see the Supplementary Note 1: Glossary of Technical Terms. For a full explanation of the concept of strong and weak dominance, see the Supplementary Discussion.

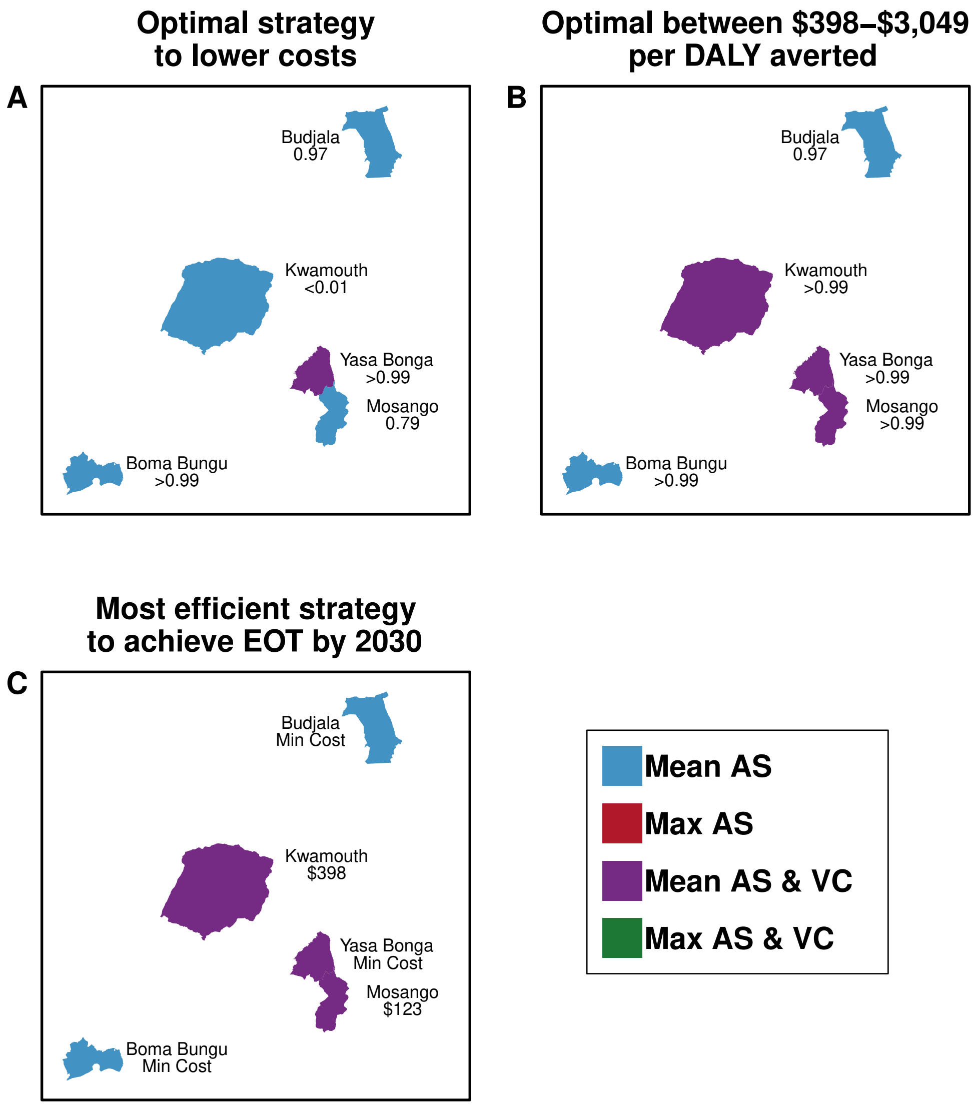

Supplementary Figure 9: Maps of preferred strategies according to economic or budgetary goals for 2020–2030. Maps A & B show the optimal strategies depending on WTP. The text indicates the probability that the optimal strategy will lead to EOT by 2030. Map C shows the optimal strategy that has at >90% probability of EOT by 2030 and shows the ICER of the indicated strategy (Mean AS for all locations except Yasa Bonga, where it is Mean AS & VC). Maps are not drawn to scale.

	Cost-effectiveness analysis without uncertainty			Net benefit (uncertainty) analysis: Prob. that a strategy is optimal, (conditional on willingness-to-pay)				
	Cost difference vs comparator	DALYs averted vs comparator	ICER	$0 USD per DALY averted	$250 per DALY averted	$500 per DALY averted	$1,000 per DALY averted	Prob. EOT by 2030
**Yasa Bonga**								
Mean AS & VC	0	0	Min Cost	0.78	0.78	0.78	0.78	>0.99
Max AS & VC	671,462	0	2,209,891	0.22	0.22	0.22	0.22	>0.99
**Mosango**								
Mean AS	0	0	Dominated	0.38	0.33	0.29	0.24	0.79
Max AS	376,544	80	Dominated	0.04	0.04	0.04	0.05	0.92
Mean AS & VC	-49,378	142	Min Cost	0.49	0.53	0.56	0.59	>0.99
Max AS & VC	236,263	166	12,216	0.08	0.09	0.1	0.13	>0.99
**Kwamouth**								
Mean AS	0	0	Dominated	0.24	0.14	0.09	0.05	<0.01
Max AS	1,052,454	667	Dominated	0	0	0	0	<0.01
Mean AS & VC	-451,240	2,976	Min Cost	0.64	0.71	0.72	0.7	>0.99
Max AS & VC	26,747	3,084	4,426	0.11	0.15	0.19	0.25	>0.99
**Boma Bungu**								
Mean AS	0	0	Min Cost	1	0.99	0.99	0.99	>0.99
Max AS	101,606	3	40,288	0	0	0	0.01	>0.99
Mean AS & VC	127,894	2	Dominated	0	0	0	0.01	>0.99
Max AS & VC	223,232	4	93,059	0	0	0	0	>0.99
**Budjala**								
Mean AS	0	0	Min Cost	0.91	0.87	0.85	0.76	0.97
Max AS	335,786	47	Weakly Dominated	0.04	0.03	0.03	0.03	>0.99
Mean AS & VC	131,746	45	2,921	0.04	0.07	0.1	0.18	>0.99
Max AS & VC	423,279	62	17,515	0.01	0.02	0.02	0.03	>0.99

Supplementary Table 24: Summary of cost-effectiveness, assuming a time horizon of 2020-2050. Cost differences and differences in disability-adjusted life-years (DALYs) averted are relative to the comparator–first strategy listed for each location. Mean DALYs averted and mean cost differences are shown; these estimates are discounted at 3 percent per year in accordance with guidelines. The uncertainty analysis (columns 5-8) shows the probability that a strategy is cost-effective. Strategies highlighted in pink are optimal strategies: the strategies for which the mean net monetary benefit (NMB) is highest, equivalent to the information found in cost-effectiveness acceptability frontiers (CEAFs), which are shown Figures 7- 8. ICER: incremental cost-effectiveness ratio. For an extended discussion of these terms, see the Supplementary Note 1: Glossary of Technical Terms. For a full explanation of the concept of strong and weak dominance, see the Supplementary Discussion.

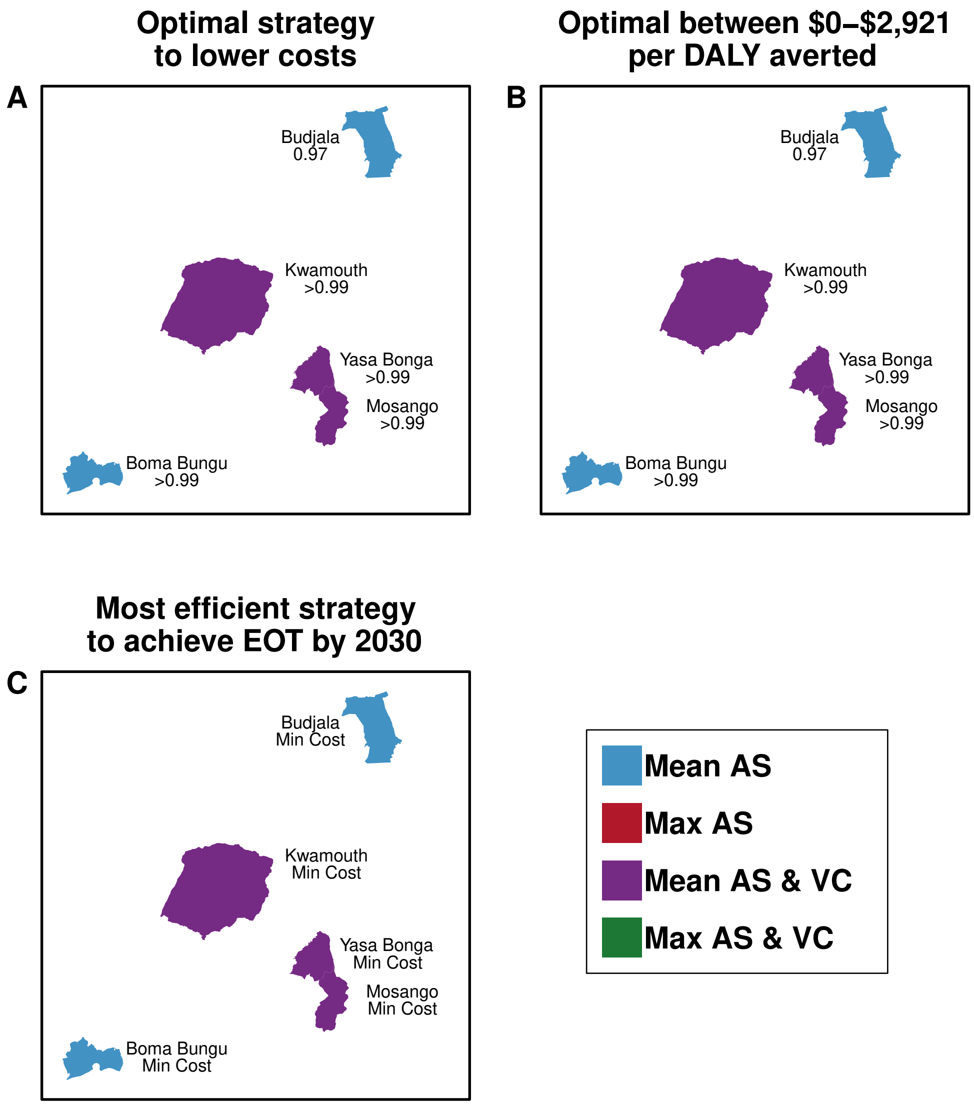

Supplementary Figure 10: Maps of preferred strategies according to economic or budgetary goals for 2020–2050. Maps A & B show the optimal strategies depending on WTP. The text indicates the probability that the optimal strategy will lead to EOT by 2030. Map C shows the optimal strategy that has at >90% probability of EOT by 2030 and shows the ICER of the indicated strategy (Mean AS for all locations except Yasa Bonga, where it is Mean AS & VC). Maps are not drawn to scale.

B.6 Sensitivity analysis: vector control

Strategy	VC effectiveness: 60% tsetse annual decline		VC effectiveness: 80% tsetse annual decline		VC effectiveness: 90% tsetse annual decline	
	ICER	Pr EOT by 2030	ICER	Pr EOT by 2030	ICER	Pr EOT by 2030
**100 kilometers; 20 targets per kilometer**						
Mean AS	Dominated	0.79	Dominated	0.79	Dominated	0.79
Max AS	Dominated	0.92	Dominated	0.92	Dominated	0.92
Mean AS & VC	Min Cost	>0.99	Min Cost	>0.99	Min Cost	>0.99
Max AS & VC	11,245	>0.99	12,444	>0.99	14,224	>0.99
**210 kilometers; 20 targets per kilometer**						
Mean AS	Min Cost	0.79	Min Cost	0.79	Min Cost	0.79
Max AS	Dominated	0.92	Dominated	0.92	Dominated	0.92
Mean AS & VC	480	>0.99	218	>0.99	142	>0.99
Max AS & VC	10,815	>0.99	12,017	>0.99	13,720	>0.99
**100 kilometers; 40 targets per kilometer**						
Mean AS	Dominated	0.79	Dominated	0.79	Dominated	0.79
Max AS	Dominated	0.92	Dominated	0.92	Dominated	0.92
Mean AS & VC	Min Cost	>0.99	Min Cost	>0.99	Min Cost	>0.99
Max AS & VC	11,009	>0.99	12,215	>0.99	13,940	>0.99
**210 kilometers; 40 targets per kilometer**						
Mean AS	Min Cost	0.79	Min Cost	0.79	Min Cost	0.79
Max AS	Dominated	0.92	Dominated	0.92	Dominated	0.92
Mean AS & VC	2,051	>0.99	1,626	>0.99	1,488	>0.99
Max AS & VC	10,320	>0.99	11,535	>0.99	13,124	>0.99

Supplementary Table 25: Incremental cost-effectiveness ratios (ICERs) under different assumptions for vector control in Mosango. Costs and effects were discounted, and the investment horizon is 2020-2040.

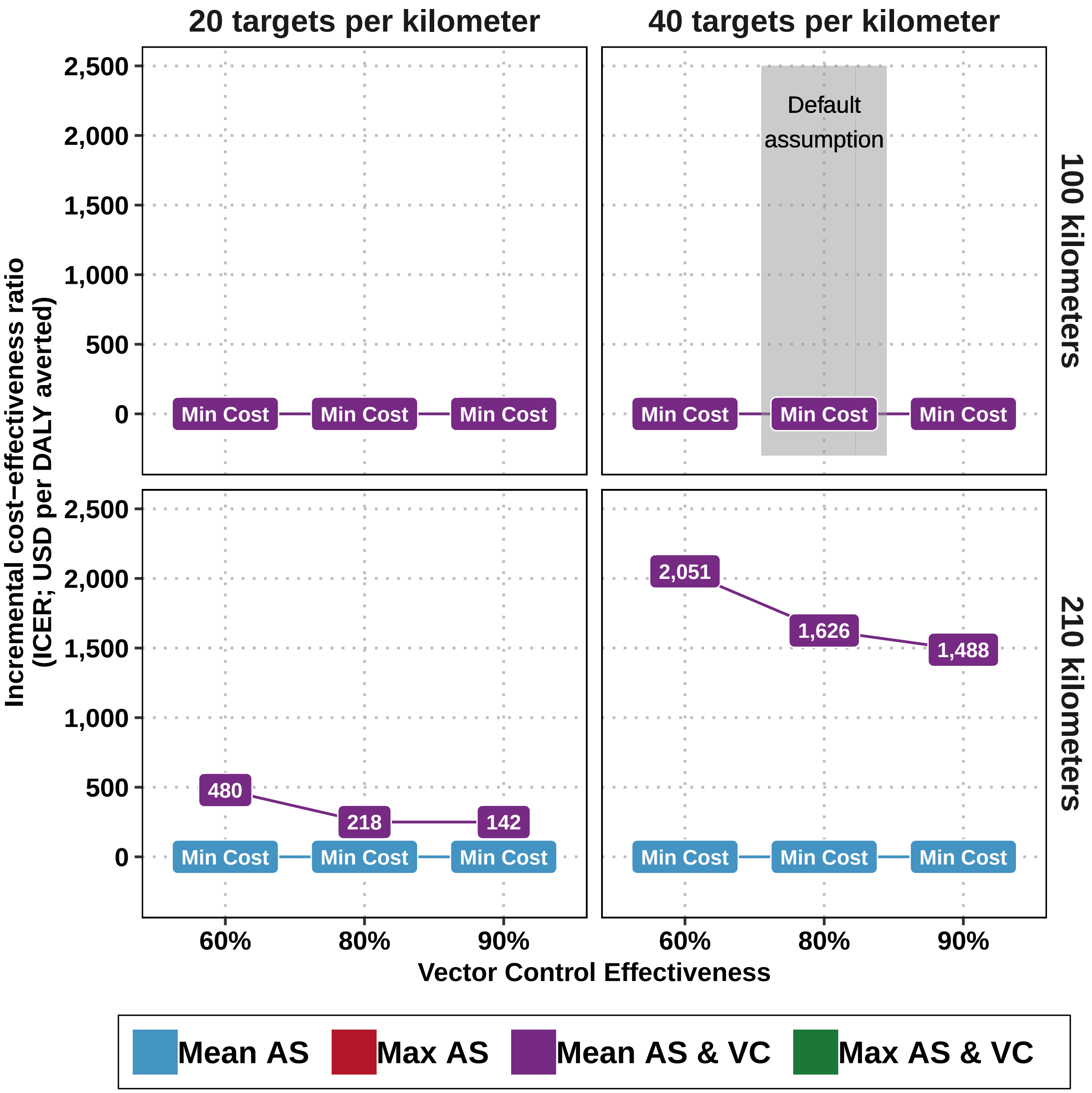

Supplementary Figure 11: Sensitivity analyses of incremental cost-effectiveness ratios (ICERs) with alternative assumptions of the effectiveness of vector control, the extent to which the health zone must have vector control deployed, and the intensity of targets deployed (targets per kilometer) in Mosango. Costs and effects are discounted, and the investment horizon is 2020-2040. Dominated or weakly dominated strategies are not included in this figure.

Strategy	VC effectiveness: 60% annual decline of tsetse populations ICER	Pr EOT by 2030	VC effectiveness: 80% annual decline of tsetse populations ICER	Pr EOT by 2030	VC effectiveness: 90% annual decline of tsetse populations ICER	Pr EOT by 2030
**20 targets per kilometer**						
Mean AS	Min Cost	<0.01	Min Cost	<0.01	Dominated	<0.01
Max AS	Dominated	<0.01	Dominated	<0.01	Dominated	<0.01
Mean AS & VC	59	>0.99	4	>0.99	Min Cost	>0.99
Max AS & VC	3,937	>0.99	4,421	>0.99	4,606	>0.99
**40 targets per kilometer**						
Mean AS	Min Cost	<0.01	Min Cost	<0.01	Min Cost	<0.01
Max AS	Dominated	<0.01	Dominated	<0.01	Dominated	<0.01
Mean AS & VC	343	>0.99	258	>0.99	236	>0.99
Max AS & VC	3,873	>0.99	4,373	>0.99	4,548	>0.99

Supplementary Table 26: Incremental cost-effectiveness ratios (ICERs) under distinct assumptions for vector control

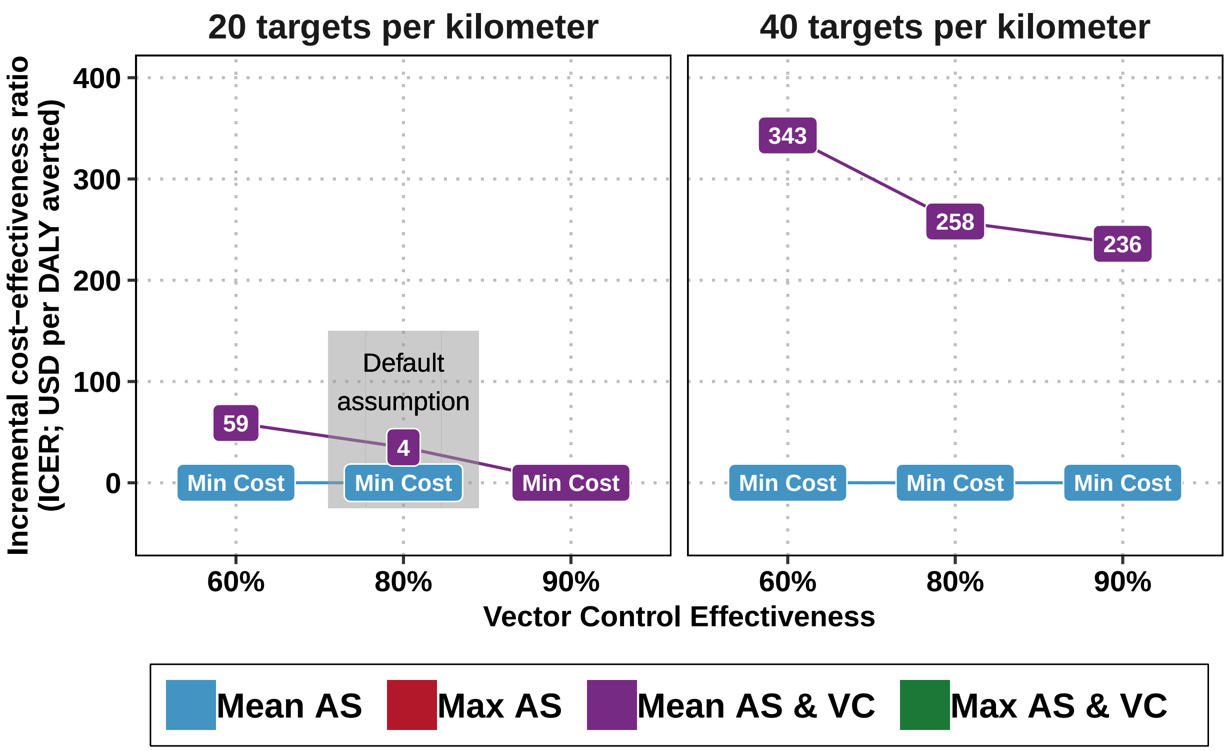

Supplementary Figure 12: Sensitivity analyses of incremental cost-effectiveness ratios (ICERs) with alternative assumptions of the effectiveness of vector control and the intensity of targets deployed (targets per kilometer) in Kwamouth. In Kwamouth, it is estimated that vector control will be need to be deployed along 390 km of the river Kwa and 42 km of river in Kwamouth East (for a total of 432 km of river). Costs and effects are discounted, and the investment horizon is 2020-2040. Dominated or weakly dominated strategies are not depicted (see Supplementary Discussion for a discussion on weak and strong dominance).

C Supplementary Discussion

C.1 Incremental cost-effectiveness ratios, dominance, and weak dominance

We give here a step-by-step calculation of incremental cost-effectiveness ratios (ICERs) and how their calculation could lead to the conclusion that a strategy is under strong or weak dominance.

Below is the calculation for Kwamouth, assuming an investment horizon of ten years (2020–2030). Preliminary

Strategy	Cost Difference	DALYs averted	Preliminary ICER	Final ICER
**2020-2030**				
Mean AS	0	0	Min Cost	Min Cost
Max AS	574,743	440	1,307	Weakly Dominated
Mean AS & VC	816,312	2,049	150	398
Max AS & VC	1,276,239	2,156	4,263	4,263

Supplementary Table 27: Computing incremental cost-effectiveness ratios (ICERs) for Kwamouth. DALYs: disability-adjusted life-years.

ICERs display the difference in costs and effects between a given strategy and the next strategy of lesser value (in Kwamouth, it would be the intervention in the previous line, but Budjala the progression to more expensive interventions is more complicated). Due to diminishing returns, ICERs increase with more ambitious interventions. Some ICERs (shown in red) show that the strategy delivers a worse-value-for-money the next best intervention (in the following line). The strategies with red ICERs are 'weakly dominated' and should not be adopted. The final ICERs show the ICERs once weakly dominated strategies have been removed. The ICERs highlighted in yellow are ICERs that have changed with the removal of weakly dominated strategies.

Strategy	Cost Difference	DALYs averted	Preliminary ICER	Final ICER
**2020-2040**				
Mean AS	0	0	Min Cost	Min Cost
Max AS	335,786	47	91,025	Weakly Dominated
Mean AS & VC	131,747	45	2,922	2,922
Max AS & VC	423,280	62	6,075	17,515

Supplementary Table 28: Computing incremental cost-effectiveness ratios (ICERs) for Budjala. DALYs: disability-adjusted life-years. AS: active screening. VC: vector control.

D Supplementary Note 1: Glossary of Technical Terms

Box 1: Glossary

EPIDEMIOLOGY TERMS

Intervention Interventions are separate activities to address a health need (e.g. active screening (AS) or vector control (VC)).

Strategy A strategy is a combination of interventions, carried out with a specific coverage, and in parallel. In this paper, we simulate strategies with and without an improvement in AS and with and without VC (e.g. strategy 1 is passive surveillance (PS) and mean AS, and strategy 4 is PS, maximum AS and VC).

Elimination of transmission (EOT) Globally this is the 2030 goal for gHAT; here we also consider local EOT for health zones. The feasibility of EOT is expressed as a probability equal to the proportion of our simulations in which new infections is zero before a given year (usually 2030).

Disability-adjusted life-year (DALY) In order to present the burden of disease in one common metric across diseases, DALYs are calculated in cost-effectiveness analyses. This is the sum of the years lived with disability due to the disease and the years of life lost by fatal cases. See section A.3.

HEALTH ECONOMICS TERMS

Parameter uncertainty Uncertainty in the level of transmission or in the costs of interventions and treatment due to unknown underlying parameters (see supplementary section G for an explanation of our parameterization of the health outcomes and cost model).

Willingness-to-pay (WTP) or cost-effectiveness threshold The amount of money that payers would pay to avert one DALY arising from the disease in the analysis (gHAT). No specific threshold is recommended, but a recent analysis shows that the WTP in DRC is between 5 - 230 USD per DALY averted [31–33].

Incremental cost-effectiveness ratio A ratio of marginal cost for a marginal benefit, calculated as follows:

$$\text{ICER} = \frac{\Delta\text{Costs}}{\Delta\text{DALYs}} = \frac{\text{Costs}_{\text{strategy}} - \text{Costs}_{\text{next best}}}{\text{Effects}_{\text{strategy}} - \text{Effects}_{\text{next best}}}$$

For an example on how interventions are ranked and ICERs are calculated, see Section C.1.

Cost-effective strategy The strategy where the ICER is less than the WTP (or cost-effectiveness threshold). We say that the cost-effective strategy is “conditional" on the WTP.

Dominated strategy A strategy that costs more than the minimum cost intervention while reducing the burden by a smaller degree. This strategy ought not be implemented.

Weakly dominated strategy (or strategies under extended dominance) A strategy in which the ICER is higher that the next more expensive strategy. See section C.1 for a discussion on this matter with respect to the strategies presented in this analysis.

Net monetary benefit The net benefits (NMB) framework is derived from ICERs, but also takes uncertainty into account.

$$\text{NMB}|\text{WTP} : \text{WTP} \times \Delta\text{DALYs} - \Delta\text{Costs}$$

The optimal strategy at a given WTP is the strategy with the highest mean NMB at that value of WTP.

Optimal strategy Analogous to the cost-effective strategy when no uncertainty is assumed, this is the strategy that is recommended by the NMB framework.

E Supplementary Note 2: PRIME Criteria

Principle and what has been done to satisfy the principle?	Where in the manuscript is this described?
**1. Stakeholder engagement**<br>Strategy components were determined along with the country director of PNLTHA, Erick Miaka (co-author). Implementation of simulations of AS and PS costs were aided by Rian Snijders, who has helped run field operations in former Bandundu, and implementation of costs of VC were aided by Rian Snijders and collaborators at the Liverpool School of Tropical Medicine, who have run field operations in DRC since 2015.	Authorship list and acknowledgements
**2. Complete model documentation**<br>Full model (including the fitting code) and documentation are available through OpenScienceFramework (OSF). The epidemiological model is fully described in the fitting study of Crump *et al* [1] and cost model in this Supplementary Methods.	Description in Supplementary Methods, section A.2 and access the code via `5HZCEAOSF`[1] and `ProjOSF`[2].
**3. Complete description of data used**<br>Information about the data used for fitting is described in Crump *et al* [1]. The data used for clinical outcomes and costs were estimates from the literature. No data from intervention operations was used. Assumptions and estimates were parameterized according to conventions in the economic evaluation literature [35].	For assumptions around intervention, treatment effects and costs, see Supplementary Methods, sections A.3.1 and A.4, as well as Supplementary Note 4: Parameter Glossary.
**4. Communicating uncertainty**<br>*Structural uncertainty*:<br>Uncertainty arising from the choice of vector control operation inputs, discounting, time horizon are shown by re-running the entire analysis with alternative assumptions.	Main text results and discussion. A three-way sensitivity analysis for VC operation is in Supplementary Tables and Figures, section B.6, and time horizons and discounting two-way sensitivity analysis is in Supplementary Tables and Figures, section B.5.
Parameter uncertainty:<br>The epidemiological parameters are the posterior distributions of a model fitted to time-series data, and full details are available in another publication [1]. For the parameters to model health outcomes and costs, assumptions and estimates were parameterized according to conventions in the economic evaluation literature [35], taking care to sample from large distributions for aspects for which we knew very little.	Epidemiological parameters are available on OSF `FittingOSF` [3]. Health outcome and cost-effectiveness parameters: A.3.1, A.4, Supplementary Note 4: Parameter Glossary. Sensitivity analysis for VC in are described in Supplemenetary Methods, section A.6 and the results are found in Supplementary Tables and Figures, section B.6 and in the `GUI`[4].
Prediction uncertainty:<br>Observational uncertainty in epidemiological model predictions. Tables 2 and 3 present both means and 95% prediction intervals. The `GUI`[4] includes box and whisker plots to show uncertainty in cases, deaths and DALYs. We include the probability of meeting EOT by 2030 as well as the expected year of EOT. For cost-effectiveness results, we present the optimal decisions (the probability of each of being cost-effective) at different willingness-to-pay thresholds rather than solely providing ICERs using the net benefits framework (see Table 4).	Tables 2-4 of the manuscript and the `GUI`[4].
**5. Testable model outcomes**<br>Epidemiological model outputs are routinely reported metrics of the disease course: detected active and passive case detections. Therefore, these predictions can be compared to future data as long as the data is put into context alongside measures of active screening coverage and the number of fixed health posts equipped for passive surveillance. Some components of costs predictions can be validated against expenditures, but it must be noted that these are economic costs, and so resource use for which there is no explicit invoice is taken into account as well.	Epidemiological projections for the period 2020-2040 are shown in Table 3, and for the period ending in 2030 in Supplementary Table 16 and for the period ending in 2050 in Supplementary Table 17. Epidemiological outcomes for each year until 2050 can be viewed in the `GUI`[4]. All the ingredients to the economic costs were shown in detail in the Supplementary Methods, section A.4.

[1] `5HZCEAOSF` with full address: `https://osf.io/xbwte/`.
[2] `ProjOSF` with full address: `https://osf.io/jza27/`.
[3] `FittingOSF` with full address: `https://osf.io/ck3tr/`.
[4] `GUI` with full address: `https://hatmepp.warwick.ac.uk/5HZCEA/v2/`

Supplementary Table 29: PRIME-NTD criteria fulfilment. We summarise how the NTD Modelling Consortium's "5 key principles of good modelling practice" have been met in the present study [34].

F Supplementary Note 3: CHEERS Checklist

Supplementary Table 30: Consolidated Health Economic Evaluation Reporting Standards (CHEERS) checklist.

Section/item	Item No	Recommendation	Section Reported
Title	1	Identify the study as an economic evaluation or use more specific terms such as “cost-effectiveness analysis”, and describe the interventions compared.	Cover page
Abstract	2	Provide a structured summary of objectives, perspective, setting, methods (including study design and inputs), results (including base case and uncertainty analyses), and conclusions.	Abstract
Background and Objectives	3	Provide an explicit statement of the broader context for the study. Present the study question and its relevance for health policy or practice decisions.	Introduction section, last paragraph in particular.
Target population and subgroups	4	Describe characteristics of the base case population and subgroups analysed, including why they were chosen.	First paragraph of the results. Ordinarily we would put this in the methods, but the occurrence of the results before the methods made the results a more useful place to put this portion.
Settings and location	5	State relevant aspects of the system(s) in which the decision(s) need(s) to be made.	Second subsection of the methods, "Strategies", illustrated in Figure 1. Locations are illustrated in Supplementary Methods, section A.1.
Study perspective	6	Describe the perspective of the study and relate this to the costs being evaluated.	Fifth subsection of the methods, "Costs".
Comparators	7	Describe the interventions or strategies being compared and state why they were chosen.	Second subsection of the methods, "Strategies", and Figure 1. Supplementary Methods, section A.2.2.
Time horizon	8	State the time horizon(s) over which costs and consequences are being evaluated and say why appropriate.	First paragraph of the ’Cost-effectiveness’ subsection of the results. Ordinarily we would put this in the methods, but the occurrence of the results before the methods made the results a more useful place to put this portion.
Discount rate	9	Report the choice of discount rate(s) used for costs and outcomes and say why appropriate.	First paragraph of the ’Cost-effectiveness’ subsection of the results. 3% is the recommended rate by WHO-CHOICE and the Gates Reference Case.
Choice of health outcomes	10	Describe what outcomes were used as the measure(s) of benefit in the evaluation and their relevance for the type of analysis performed.	Fourth subsection of the methods, "Cases, deaths, and treatment outcome model". Supplementary Methods, sections A.2.3 and A.3 contain detailed explanations of how DALYs are calculated and how the natural history of HAT was considered.
	11a	*Single study-based estimates:* Describe fully the design features of the single effectiveness study and why the single study was a sufficient source of clinical effectiveness data.	NA

CHEERS checklist (continued)			
**Section/item**	**Item No**	**Recommendation**	**Reported on page no, line no**
of effectiveness	11b	*Synthesis-based estimates:* Describe fully the methods used for identification of included studies and synthesis of clinical effectiveness data.	Effectiveness of AS strategies and PS strategies: model-based, of VC: sensitivity analysis, of screening tests and treatment: the literature. Described in detail in the first section of the Methods, "Transmission model", and the fourth section "Cases, deaths, and treatment outcome model", as well as Supplementary Methods A.2-A.3, and Supplementary Note 4: Parameter Glossary.
Measurement and valuation of preference based outcomes	12	If applicable, describe the population and methods used to elicit preferences for outcomes.	NA
Estimating resources and costs	13a	*Single study-based economic evaluation:* Describe approaches used to estimate resource use associated with the alternative interventions. Describe primary or secondary research methods for valuing each resource item in terms of its unit cost. Describe any adjustments made to approximate to opportunity costs.	NA
	13b	*Model-based economic evaluation:* Describe approaches and data sources used to estimate resource use associated with model health states. Describe primary or secondary research methods for valuing each resource item in terms of its unit cost. Describe any adjustments made to approximate to opportunity costs.	The construction of program costs are detailed in Supplementary Methods, section A.4. As can be seen, in a publication of this scope, detailing the cost inputs in the main body would be unfeasible. However, the resulting expected costs are in Table 3 of the main body of the paper.
Currency, price date, and conversion	14	Report the dates of the estimated resource quantities and unit costs. Describe methods for adjusting estimated unit costs to the year of reported costs if necessary. Describe methods for converting costs into a common currency base and the exchange rate.	Our general approach for this is in the section "Principles for parameterization" Supplemental Note 4: Parameter Glossary, Section G.1 and the specific choices for each parameter follow in Supplementary Note 4: Parameter Glossary.
Choice of model	15	Describe and give reasons for the specific type of decision- analytical model used. Providing a figure to show model structure is strongly recommended.	The decision analytic model is discussed in Supplementary Methods, sections A.5 and illustrated in Supplementary Figure 4, but the components models feeding into the decision analytic model are described as follows: the dynamic transmission (SEIRS) model is described briefly in the Supplementary Methods, section A.2.1, and the treatment model is shown in Figure 2 and described in Supplementary Methods, section A.2.3.
Assumptions	16	Describe all structural or other assumptions underpinning the decision-analytical model.	The second and third sections of the Methods and all of the Supplementary Methods.
Analytical methods	17	Describe all analytical methods supporting the evaluation. This could include methods for dealing with skewed, missing, or censored data; extrapolation methods; methods for pooling data; approaches to validate or make adjustments (such as half cycle corrections) to a model; and methods for handling population heterogeneity and uncertainty.	Section A.5 and the parameter glossary, Section G.

CHEERS checklist (continued)			
**Section/item**	**Item No**	**Recommendation**	**Reported on page no, line no**
Study parameters	18	Report the values, ranges, references, and, if used, probability distributions for all parameters. Report reasons or sources for distributions used to represent uncertainty where appropriate. Providing a table to show the input values is strongly recommended.	Section G.
Incremental costs and outcomes	19	For each intervention, report mean values for the main categories of estimated costs and outcomes of interest, as well as mean differences between the comparator groups. If applicable, report incremental cost-effectiveness ratios.	Table 3 in the main body, and Supplementary Tables 16-17 for alternative time horizons.
Characterising uncertainty	20a	Single study-based economic evaluation: Describe the effects of sampling uncertainty for the estimated incremental cost and incremental effectiveness parameters, together with the impact of methodological assumptions (such as discount rate, study perspective).	NA
	20b	Model-based economic evaluation: Describe the effects on the results of uncertainty for all input parameters, and uncertainty related to the structure of the model and assumptions.	Table 4 in the main body, and Suppplementary Tables 23- 24 for alternative time horizons. In addition, cost-effectiveness acceptability curves are depicted in Supplementary Figures 7-8. The interpretation is in section of the results entitled "Cost-effectiveness in the presence of probabilitic uncertainty" and in the sixth paragraph of the discussion.
Characterising heterogeneity	21	If applicable, report differences in costs, outcomes, or cost-effectiveness that can be explained by variations between subgroups of patients with different baseline characteristics or other observed variability in effects that are not reducible by more information.	Results section. The analysis is framed as five case studies to show the impacts of heterogeneity.
Study findings, limitations, generalisability, and current knowledge	22	Summarise key study findings and describe how they support the conclusions reached. Discuss limitations and the generalisability of the findings and how the findings fit with current knowledge.	Discussion.
Source of funding	23	Describe how the study was funded and the role of the funder in the identification, design, conduct, and reporting of the analysis. Describe other non-monetary sources of support.	Last section of the methods and the Acknowledgements section.
Conflicts of interest	24	Describe any potential for conflict of interest of study contributors in accordance with journal policy. In the absence of a journal policy, we recommend authors comply with International Committee of Medical Journal Editors recommendations.	Conflict of interest statement.

G Supplementary Note 4: Parameter Glossary

G.1 Principles for parameterization . . . 54
G.2 Organization of parameters . . . 54
G.3 Summary of health outcome parameters . . . 56
G.4 Summary of cost parameters . . . 57
G.5 Screening parameters . . . 58
G.5.1 Population . . . 58
G.5.2 PS: coverage of the population per facility . . . 58
G.5.3 PS: number of facilities . . . 58
G.5.4 AS: coverage . . . 59
G.5.5 AS: coverage, enhanced . . . 59
G.5.6 AS: maximum capacity by each team per year . . . 59
G.5.7 CATT algorithm: diagnostic specificity . . . 59
G.5.8 RDT algorithm: diagnostic sensitivity . . . 60
G.5.9 RDT algorithm: diagnostic specificity . . . 60
G.5.10 CATT algorithm: wastage during AS . . . 60
G.5.11 RDT algorithm: wastage during PS . . . 60
G.6 Treatment parameters . . . 61
G.6.1 Proportion of cases age<6 . . . 61
G.6.2 Proportion of cases weight<35 kg among age>6 . . . 61
G.6.3 Proportion of S2 cases that are severe . . . 63
G.6.4 Age of death from infection . . . 63
G.6.5 Length of hospital stay: NECT treatment . . . 64
G.6.6 Length of hospital stay: fexinidazole treatment . . . 65
G.6.7 Pr. of relapse: pentamidine . . . 65
G.6.8 Pr. of relapse (treatment failure): NECT . . . 65
G.6.9 Pr. of relapse: fexinidazole . . . 66
G.6.10 SAE: pentamidine treatment . . . 67
G.6.11 SAE: NECT treatment . . . 67
G.6.12 SAE: fexinidazole treatment . . . 68
G.6.13 Days lost to disability: due to S1 disease . . . 68
G.6.14 Days lost to disability: due to S2 disease . . . 69
G.6.15 Days lost to disability: due to SAE . . . 69
G.7 Life-years lost (DALY) parameters . . . 69
G.7.1 Life expectancy . . . 69
G.7.2 Disability weights: S1 disease . . . 69
G.7.3 Disability weights: S2 disease . . . 70
G.7.4 Disability weights: SAE . . . 70
G.8 Vector control parameters . . . 71
G.8.1 Linear km of targets . . . 71
G.8.2 Target per km . . . 71
G.8.3 Replacement rate of targets per year . . . 71
G.9 Screening cost parameters . . . 71
G.9.1 AS: capital costs of a team . . . 71
G.9.2 AS: fixed management costs of a team . . . 72
G.9.3 CATT algorithm: cost per test used . . . 72
G.9.4 Staging: lumbar puncture & lab exam . . . 73
G.9.5 Confirmation: microscopy . . . 73
G.9.6 RDT algorithm: costs per test used . . . 73
G.9.7 Variable management costs (PNLTHA mark-up) . . . 74
G.9.8 PS: capital costs of a facility . . . 74
G.9.9 PS: fixed recurrent management costs . . . 74
G.10 Treatment cost parameters . . . 75

G.10.1 Hospital stay: cost per day 75
G.10.2 Outpatient consultation: cost 75
G.10.3 Course of pentamidine: cost 76
G.10.4 Course of NECT: cost 76
G.10.5 Course of fexinidazole: cost 76
G.10.6 Drug delivery mark-up 76
G.11 Vector control cost parameters 77
G.11.1 Operational cost per kilometer of riverbank covered 77
G.11.2 Deployment cost per target 77

G.1 Principles for parameterization

These are the rules I have for myself to parameterize my models. The rationale behind some of the items in my tables are easier to understand with these rules.

- **Transferability of costs across time** Costs have been updated to 2018 USD values. All costs are converted to local currency units in the year of the study, inflated to 2018 values using the consumer price index (CPI) of the country, and then converted to USD using the exchange rate in 2018. It should be noted that the 2003 WHO Guide to Cost-effectiveness recommends that the GDP inflator be used (see 3.2.6 Transferability of costs across time, page 43) but we found that the data on this measure (from the World Bank) were sometimes sparse so we relied on the consumer price index instead [19].

- **Transferability of costs across settings** To 'borrow' data from other countries, we follow the 2003 WHO Guide to Cost-effectiveness recommendations in section 3.2.7 Transferability of costs across settings) [19]. For non-traded items (nurse and doctor time) we convert USD or LCU prices into PPP (international dollars) values in the year of the cost study and then turn the value in international dollars to local currency (still in the year of the study) of the country where a cost estimate is needed. Then, we use the CPI to inflate costs to 2018 levels and then use the exchange rate with USD to get 2018 USD values.

- **Combining multiple sources of information** Values from different publications are combined using meta-analytic methods.

- **Choice of probability distributions** Costs and ratios were modelled via gamma distributions and proportions or probability were modelled with beta distributions. These distributions were parameterized using the method of moments (see Briggs 2006 [26], Chapter 4).

- **Missing information on uncertainty: Gamma distributions.**

1. Option A: Whenever uncertainty was missing for a cost or ratio, we assigned the parameter a gamma distribution that would yield credible intervals between half and double the estimate available in the literature.
2. Option B: If at least 2 studies listed a cost, then we take the range of the costs to parameterize a gamma distribution for which the the 95 percent confidence interval (the 2.5th and 97.5th percentile) matches the range of estimates from the literature. For these occasions, we use a method to parameterize gamma distributions using quantiles rather than using the mean and standard error of a sample (method of moments) [36].

- **Missing information on uncertainty: Beta distributions.**

1. Option A: Usually modeled assuming that 100 trials were observed with the **proportion_estimate x 100** as the alpha parameter and **(1-proportion_estimate) x 100** as beta parameter.
2. Option B: If at least 2 studies listed a probability or a proportion, then we take the range of the costs to parameterize a beta distribution by assuming the range matches the 95 percent confidence interval (the 2.5th and 97.5th percentile). We use a method to parameterize Beta distributions using quantiles rather than the mean and standard error of a sample (method of moments) [37].

G.2 Organization of parameters

In the code repository, found in `https://osf.io/xbwte/`, the code makes use of five r lists: surv_cost, surv_effect, treat_effect, treat_cost, vector_effect, vector_cost, which are read in from an excel sheet "parameters.xlsx". Within those lists sit a set of variables described in the tables later in this document.

- `surv_effect`: these are the parameters related to the consumption (units) and effectiveness of screening and passive screening.
- `surv_cost`: these are the costs of active screening and passive screening. The units are either general demographic units (cases or population) or parameters found in surv_effect.
- `treat_effect`: these are the parameters related to the consumption (units) and effectiveness of treatment.
- `treat_cost`: these are the costs of treatment. The units are either general demographic units (cases or population) or parameters found in treat_effect.

- `vector_effect`: these are the parameters related to the consumption (units) and effectiveness of treatment.
- `vector_cost`: these are the costs of treatment. The units are either general demographic units (cases or population) or parameters found in treat_effect.

Additionally, a list named `epi_output` (read in from Matlab output) holds the output from the dynamic model: stage 1 and stage 2 cases detected by passive and active screening.

G.3 Summary of health outcome parameters

Below are all the parameters that model health outcomes, as well as a summary of their characteristics. An extended discussion of our choices is featured in the sections annotated in the table.

Variable description	Variable name	Statistical Distribution	Descriptive Summary	Notes
**Screening**				
Population	surv_effect[["pop"]]	Fixed value	Varies by health zone	See section G.5.1
PS: coverage of the population per facility	surv_effect[["ps_coverage"]]	Beta(14, 2094)	0.007 (0.004, 0.010)	See section G.5.2
PS: number of facilities	surv_effect[["ps_facilities"]]	Fixed value	Varies by health zone	See section G.5.3
AS: coverage	surv_effect[["as_traditional"]]	Fixed value	Varies by health zone	See section G.5.4
AS: coverage, enhanced	surv_effect[["as_traditional_max"]]	Fixed value	Varies by health zone	See section G.5.5
AS: maximum capacity by each team per year	surv_effect[["as_traditional_capacity"]]	Normal(60000, 10000)	60,055 (40,448, 79,471)	See section G.5.6
CATT algorithm: diagnostic specificity	surv_effect[["dx_spec_catt"]]	Beta(31, 2)	0.94 (0.84, 0.99)	See section G.5.7
RDT algorithm: diagnostic sensitivity	surv_effect[["dx_sens_rdt"]]	Beta(230, 1)	1.00 (0.98, 1.00)	See section G.5.8
RDT algorithm: diagnostic specificity	surv_effect[["dx_spec_rdt"]]	Beta(226, 31)	0.88 (0.84, 0.92)	See section G.5.9
CATT algorithm: wastage during AS	surv_effect[["dx_wastage_catt_as"]]	Beta(8, 92)	0.08 (0.03, 0.14)	See section G.5.10
RDT algorithm: wastage during PS	surv_effect[["dx_wastage_rdt_ps"]]	Beta(1, 99)	0.01 (<0.01, 0.04)	See section G.5.11
**Treatment**				
Proportion of cases age<6	treat_effect[["patient_char"]] [["pc_s1s2_under6]]	Beta(152.53, 2427.9)	0.06 (0.05, 0.07)	See section G.6.1
Proportion of cases weight<35 kg among age>6	treat_effect[["patient_char"]] [["pc_s1s2_under35kg]]	Beta(8.3, 359.6)	0.02 (<0.01, 0.04)	See section G.6.2
Proportion of S2 cases that are severe	treat_effect[["patient_char"]] [["pc_s2_late]]	Beta(76.93, 44.87)	0.63 (0.54, 0.72)	See section G.6.3
Age of death from infection	treat_effect[["yld_yll"]] [["aoi_s1s2_years]]	Gamma(148, 0.18)	26.63 (22.41, 31.08)	See section G.6.4
Length of hospital stay: NECT treatment	treat_effect[["length_hosp_stay"]] [["lhs_s2_nect_days]]	Fixed value	10	See section G.6.5
Length of hospital stay: fexinidazole treatment	treat_effect[["length_hosp_stay"]] [["lhs_s1s2_fexinidazole_days]]	Fixed value	10	See section G.6.6
Pr. of relapse: pentamidine	treat_effect[["treatment_failure"]] [["tf_prob_s1_pentamidine]]	Beta(50.3, 665.48)	0.07 (0.05, 0.09)	See section G.6.7
Pr. of relapse (treatment failure): NECT	treat_effect[["treatment_failure"]] [["tf_prob_s2_nect]]	Beta(15.87, 378.55)	0.05 (0.02, 0.08)	See section G.6.8
Pr. of relapse: fexinidazole	treat_effect[["treatment_failure"]] [["tf_prob_s1s2_fexinidazole]]	Beta(9.49, 496.54)	0.02 (<0.01, 0.03)	See section G.6.9
SAE: pentamidine treatment	treat_effect[["sae"]] [["sae_prob_s1_pentamidine]]	Beta(1.43, 551.42)	0.002 (<0.001, 0.008)	See section G.6.10
SAE: NECT treatment	treat_effect[["sae"]] [["sae_prob_s2_nect]]	Beta(40.88, 367.8)	0.05 (0.03, 0.08)	See section G.6.11
SAE: fexinidazole treatment	treat_effect[["sae"]] [["sae_prob_s1s2_fexinidazole]]	Beta(3, 261)	0.01 (<0.01, 0.03)	See section G.6.12
Days lost to disability: due to S1 disease	treat_effect[["yld_yll"]] [["yld_s1_days]]	Gamma(21.89, 26.07)	569.21 (355.50, 831.29)	See section G.6.13
Days lost to disability: due to S2 disease	treat_effect[["yld_yll"]] [["yld_s2_days]]	Gamma(22.18, 12.38)	275.57 (172.46, 401.20)	See section G.6.14
Days lost to disability: due to SAE	treat_effect[["yld_yll"]] [["sae_yld_s1s2_days]]	Gamma(1.22, 2.38)	2.96 (0.14, 9.99)	See section G.6.15
**Life-years lost (DALY)**				
Life expectancy	disability[["le_years"]]	Fixed value	60.02	See section G.7.1
Disability weights: S1 disease	disability[["dw_s1"]]	Beta(22.96, 147.21)	0.14 (0.09, 0.19)	See section G.7.2
Disability weights: S2 disease	disability[["dw_s2"]]	Beta(18.37, 15.63)	0.54 (0.37, 0.70)	See section G.7.3
Disability weights: SAE	disability[["sae_dw_s1s2"]]	Uniform(0.04, 0.11)	0.08 (0.04, 0.11)	See section G.7.4
**Vector control**				
Linear km of targets	vector_effect[["vc_km"]]	Fixed value	Varies by health zone	See section G.8.1
Target per km	vector_effect[["vc_units"]]	Fixed value	Varies by health zone	See section G.8.2
Replacement rate of targets per year	vector_effect[["vc_deployments_yr"]]	Fixed value	2	See section G.8.3

Supplementary Table 31: Health outcome parameters

G.4 Summary of cost parameters

Below are all the cost parameters and a summary of their characteristics in three tables for screening, treatment, and vector control costs. An extended discussion of our choices is featured in the sections annotated in the tables.

Variable description	Variable name	Statistical Distribution	Descriptive Summary	Notes
**Screening**				
AS: capital costs of a team	surv_cost[["as_capital"]]	Gamma(81.02, 114.54)	9,276 (7,378, 11,375)	See section G.9.1
AS: fixed management costs of a team	surv_effect[["as_recurrent"]]	Gamma(63.31, 630.94)	39,955 (30,845, 50,435)	See section G.9.2
CATT algorithm: cost per test used	surv_cost[["dx_catt"]]	Gamma(25.19, 0.02)	0.52 (0.34, 0.75)	See section G.9.3
Staging: lumbar puncture \& lab exam	surv_cost[["dx_lp_lab"]]	Gamma(2.42, 3.66)	8.90 (1.45, 23.20)	See section G.9.4
Confirmation: microscopy	surv_cost[["dx_microscopy1"]]	Gamma(8.47, 1.27)	10.68 (4.70, 18.84)	See section G.9.5
RDT algorithm: costs per test used	surv_cost[["dx_rdt"]]	Gamma(8.47, 0.19)	1.60 (0.71, 2.83)	See section G.9.6
Variable management costs (PNLTHA mark-up)	surv_cost[["pnltha_mu"]]	Uniform(0.1, 0.2)	0.15 (0.10, 0.20)	See section G.9.7
PS: capital costs of a facility	surv_effect[["ps_capital"]]	Gamma(8.47, 209.8)	1,777 (778, 3,157)	See section G.9.8
PS: fixed recurrent management costs	surv_effect[["ps_recurrent"]]	Gamma(8.47, 985.55)	8,368 (3,743, 14,965)	See section G.9.9
**Treatment**				
Hospital stay: cost per day	treat_cost[["ip_day"]]	Gamma(5.81, 0.24)	1.39 (0.50, 2.71)	See section G.10.1
Outpatient consultation: cost	treat_cost[["op"]]	Uniform(1.37, 3.33)	2.34 (1.42, 3.28)	See section G.10.2
Course of pentamidine: cost	treat_cost[["rx_pentamidine"]]	Fixed value	54	See section G.10.3
Course of NECT: cost	treat_cost[["rx_nect"]]	Fixed value	460	See section G.10.4
Course of fexinidazole: cost	treat_cost[["rx_fexinidazole"]]	Fixed value	50	See section G.10.5
Drug delivery mark-up	treat_cost[["rx_delivery"]]	Beta(45, 55)	0.45 (0.35, 0.55)	See section G.10.6
**Vector control**				
Operational cost per kilometer of riverbank covered	vector_cost[["vc_operation"]]	Gamma(8.47, 14.17)	120.28 (53.33, 212.26)	See section G.11.1
Deployment cost per target	vector_cost[["vc_deploy"]]	Gamma(8.47, 0.54)	4.57 (2.02, 8.26)	See section G.11.2

Supplementary Table 32: Cost parameters

G.5 Screening parameters

G.5.1 Population

- Name in the code: `surv_effect[["pop"]]`
- Source: [38]
- Country of estimate: DRC
- Statistical distribution and parameters: Fixed value
- Summary statistics (mean and 95% CI or fixed value): Varies by health zone

Notes
Population of each health zone. See table 1.

G.5.2 PS: coverage of the population per facility

- Name in the code: `surv_effect[["ps_coverage"]]`
- Source: Personal communication with Rian Snijders, ITM Antwerp
- Country of estimate: DRC
- Statistical distribution and parameters: Beta(14, 2094)
- Summary statistics (mean and 95% CI or fixed value): 0.007 (0.004, 0.010)

Notes
In personal communications with Rian Snijders (ITM-Antwerp) we determined that each facility would screen about 3-10 people per thousand in a health zone, so health zones with more facilities screen more people than health zones with fewer facilities, and health zones that are more densely populated screen more persons per facility than health zones that are more sparsely populated. Using the method of moments, we assigned a beta distribution with mean equal to 0.0065 (rounded to 0.007 in the summary) and with standard error equal to 0.00175: Beta(14, 2094). The sample therefore has mean and confidence interval: 0.0065 (0.0035, 0.0103).

In the health zones in this study, this would yield the following results:

Health zone	Population	Number of sites	People screening per site per year (mean)	People screened per health zone per year (mean)
Yasa Bonga	221,917	4	1,442	5,770
Mosango	125,076	1	813	813
Kwamouth	131,022	5	852	4,258
Boma Bungu	85,960	2	559	1,117
Budjala	133,425	2	867	1,735

As a form of crude validation, we note that the Report of the third WHO stakeholder's meeting on gHAT elimination, replicated below, indicated 851 patients screened per center in 2017. We do not know the source population in the areas where the population was screened, but it provides a sense of the number of individuals screened per site per year.

Year	Number of functioning sites	Total people screened	People screening per site
2013	317	275,369	869
2014	317	271,436	856
2015	242	277,538	1147
2016	198	410,035	2071
2017	514	437,402	851

G.5.3 PS: number of facilities

- Name in the code: `surv_effect[["ps_facilities"]]`

- Source: [11]; see table 1
- Country of estimate: DRC
- Statistical distribution and parameters: Fixed value
- Summary statistics (mean and 95% CI or fixed value): Varies by health zone

Notes

These values were retrieved from Simarro et. al. (see the supplement) [11]. These are facilities that can perform serological tests (CATT or RDT), microbiological confirmation, and/or treatment.

G.5.4 AS: coverage

- Name in the code: `surv_effect[["as_traditional"]]`
- Source: HAT Atlas data, see table 1
- Country of estimate: DRC
- Statistical distribution and parameters: Fixed value
- Summary statistics (mean and 95% CI or fixed value): Varies by health zone

Notes

The percent of the population that is screened by mobile teams in their villages each year. The estimates are available in Table 1. These were determined by the average percent of the population in each health zone that was screened over the years 2014-2018.

G.5.5 AS: coverage, enhanced

- Name in the code: `surv_effect[["as_traditional_max"]]`
- Source: HAT Atlas data, see table 1
- Country of estimate: DRC
- Statistical distribution and parameters: Fixed value
- Summary statistics (mean and 95% CI or fixed value): Varies by health zone

Notes

The percent of the population that is screened by mobile teams in their villages each year assuming that active screening is bolstered to "maximum levels". The estimates are available in Table 1. These were determined by the maximum percent of the population in each health zone that was screened over the years 2000-2018.

G.5.6 AS: maximum capacity by each team per year

- Name in the code: `surv_effect[["as_traditional_capacity"]]`
- Source: [8–10]
- Country of estimate: DRC
- Statistical distribution and parameters: Normal(60000, 10000)
- Summary statistics (mean and 95% CI or fixed value): 60,055 (40,448, 79,471)

Notes

The capacity of an active screening team in DRC has a mean of 60,000 and a lower bound of 40,000 [8] and an upper bound of 80,000 with a work year of 220 days [10]. Teams are managed by the coordination and serve a span of multiple health zones and dozens of villages.

Capacity can vary by location, so to parameterize the model, we chose a normal distribution with upper and lower bounds of 40-80 thousand people, therefore the parameters are Normal(60,000, 10,000).

G.5.7 CATT algorithm: diagnostic specificity

- Name in the code: `surv_effect[["dx_spec_catt"]]`
- Source: [39]
- Country of estimate: Various
- Statistical distribution and parameters: Beta(31, 2)

- Summary statistics (mean and 95% CI or fixed value): 0.94 (0.84, 0.99)

Notes

Based on a systematic review by Mitashi and colleagues [39], who reported ranges of 83.5-99.3%. The beta distribution that corresponds with that range as 95% confidence intervals is Beta(31, 2).

G.5.8 RDT algorithm: diagnostic sensitivity

- Name in the code: `surv_effect[["dx_sens_rdt"]]`
- Source: [40]
- Country of estimate: Guinea and Cote d'Ivoire
- Statistical distribution and parameters: Beta(230, 1)
- Summary statistics (mean and 95% CI or fixed value): 1.00 (0.98, 1.00)

Notes

Based on a study in Guinea and Cote d'Ivoire [40], there was 1 sample from a gHAT patient that tested negative out of 231. Therefore, the parameter distribution for specifity is Beta(230, 1).

G.5.9 RDT algorithm: diagnostic specificity

- Name in the code: `surv_effect[["dx_spec_rdt"]]`
- Source: [40]
- Country of estimate: Guinea and Cote d'Ivoire
- Statistical distribution and parameters: Beta(226, 31)
- Summary statistics (mean and 95% CI or fixed value): 0.88 (0.84, 0.92)

Notes

Based on a study in Guinea and Cote d'Ivoire [40], there where 31 samples from non-HAT patients that tested positive out of 257. Therefore, the parameter distribution for specifity is Beta(226, 31).

G.5.10 CATT algorithm: wastage during AS

- Name in the code: `surv_effect[["dx_wastage_catt_as"]]`
- Source: [10]
- Country of estimate: DRC
- Statistical distribution and parameters: Beta(8, 92)
- Summary statistics (mean and 95% CI or fixed value): 0.08 (0.03, 0.14)

Notes

CATT tests come in packs of 50, and the list cost is assumed to consider that a pack is used on 50 patients. Once a pack is opened, one test is used as a positive control and one test is used as a negative control, so wastage is at least 4 percent. Shelf life of the test is one week in refrigeration and wastage in active screening activities is relatively low; generally, wastage of CATT tests in the context of active screening occurs at the end of the day when there are tests remaining in an open pack. To be conservative, we doubled the 4-percent lower bound for wastage and assigned the parameter a distribution of Beta(8, 92).

G.5.11 RDT algorithm: wastage during PS

- Name in the code: `surv_effect[["dx_wastage_rdt_ps"]]`
- Source: [12]
- Country of estimate: DRC
- Statistical distribution and parameters: Beta(1, 99)
- Summary statistics (mean and 95% CI or fixed value): 0.01 (<0.01, 0.04)

Notes

We followed the same assumption as Snijders and colleagues that less than 1 percent of RDT tests would not be used [12]. Because there was no sense of uncertainty in this parameter, we assumed a Beta (1,99) distribution.

G.6 Treatment parameters

G.6.1 Proportion of cases age<6

- Name in the code: `treat_effect[["patient_char"]][["pc_s1s2_under6]]`
- Source: [41, 42]
- Country of estimate: South Sudan
- Statistical distribution and parameters: Beta(152.53, 2427.9)
- Summary statistics (mean and 95% CI or fixed value): 0.06 (0.05, 0.07)

Notes

There were only two studies where the number of children under 5 or 6 years of age was stated explicitly [41, 42].

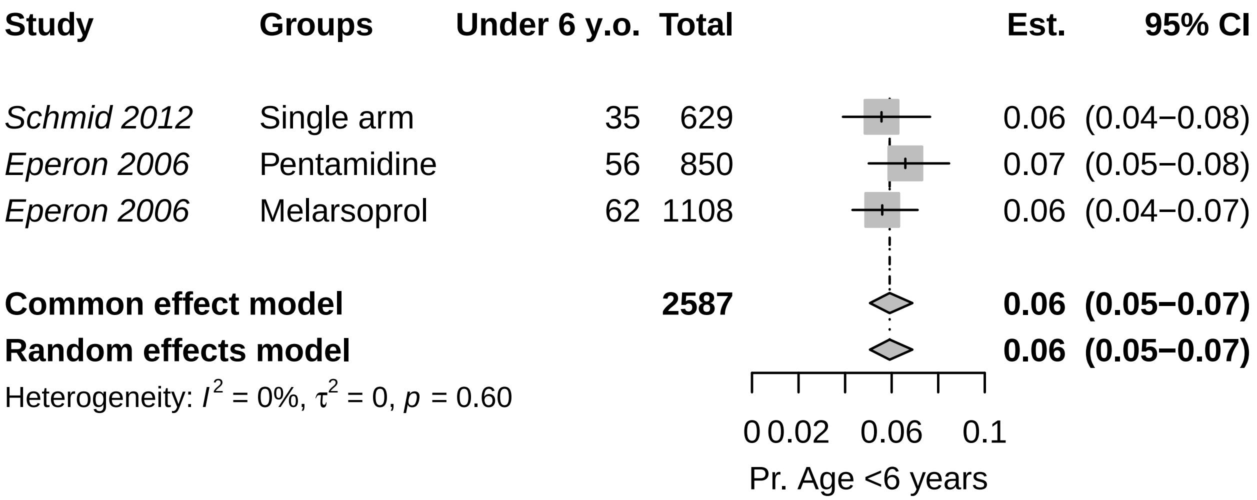

Because the data showed non-significant heterogeneity according to the tau-squared test for heterogeneity, we have chosen to use the fixed-effects combined estimate: 0.059 (0.051, 0.069). The beta parameters of the random-effects estimate Beta(153.53, 2427.90) for the probability of that a patient is under 6 years old.

G.6.2 Proportion of cases weight<35 kg among age>6

- Name in the code: `treat_effect[["patient_char"]][["pc_s1s2_under35kg]]`
- Source: [42–51]
- Country of estimate: Various
- Statistical distribution and parameters: Beta(8.3, 359.6)
- Summary statistics (mean and 95% CI or fixed value): 0.02 (<0.01, 0.04)

Notes

To determine whether a patient is eligible for fexinidazole treatment, we could not find any studies that would tell us the number of HAT patients who weighed less than 35 kg, but we have estimated the number of people that might weigh less than 35 kg by examining the distribution of weight among patients in the trials in the literature. Furthermore, we have examined how this variable is related to potential selection by age if the study population. We are interested in the proportion of older children and adults that might weigh less than 35 kg, as age under 6 is a contraindication for fexinidazole.

We fit a gamma distribution by the method of moments to the reported mean and standard deviations of each of the studies. For Priotto 2012, no SD was reported, but an inter-quartile range was reported, so we fit a gamma distribution by the method in Cook [36].

We then took the expected number of people under 35 kg, and then performed a single-proportion meta-analysis with the expected number of people in each study under and over the 35 kg-threshold.

Citation	Group	Age Group	Mean weight	Measure of spread	No. of obser-vations	Gamma distr. alpha par.	Gamma distr. beta par.	Prop. <35kg	Simulated No. <35kg
Priotto 2006	Melarsoprol and Nifurtimox	All ages	49.20	SD = 14.4	18	11.67	4.21	0.16	3
Priotto 2006	Melarsoprol and Eflornithine	All ages	50.00	SD = 10.3	19	23.56	2.12	0.06	1
Priotto 2006	NECT	All ages	51.40	SD = 8.4	17	37.44	1.37	0.02	0
Priotto 2007	NECT	Over 15 years old	51.70	SD = 7.4	52	48.81	1.06	0.01	0
Priotto 2007	Eflornithine	Over 15 years old	53.10	SD = 7.2	51	54.39	0.98	0.00	0
Checchi 2007	NECT	All ages	44.80	SD = 15.1	31	8.80	5.09	0.28	9
Priotto 2009	NECT	Over 15 years old	53.00	SD = 8.7	143	37.11	1.43	0.01	2
Priotto 2009	Eflornithine	Over 15 years old	53.90	SD = 8.3	143	42.17	1.28	0.01	1
Ngoyi 2010	Pentamidine and Melarsoprol	Over 12 years old	56.00	SD = 10.0	360	31.36	1.79	0.01	3
Priotto 2012	Single arm	All ages	49.00	IQR: 40-56	2190	16.37	2.96	0.12	265
Schmid 2012	Single arm	All ages	45.00	SD = 16.0	629	7.91	5.69	0.29	182
Burri 2016	Pentamidine	Over 15 years old and >35 kg	48.50	SD = 7.6	40	40.83	1.19	0.03	1
Pohlig 2016	Pentamidine	Over 12 years old and >30 kg	45.70	SD = 7.8	137	34.15	1.34	0.08	10
Pohlig 2016	Pafuramidine	Over 12 years old and >30 kg	44.70	SD = 7.9	136	32.02	1.40	0.10	14
Kansiime 2018	All	Over 15 years old	51.69	SD = 9.7	109	28.22	1.83	0.03	3
Mesu 2018	NECT	Over 15 years old	50.70	SD = 9.6	130	27.89	1.82	0.04	5
Mesu 2018	Fexinidazole	Over 15 years old	50.50	SD = 8.2	264	37.93	1.33	0.02	5

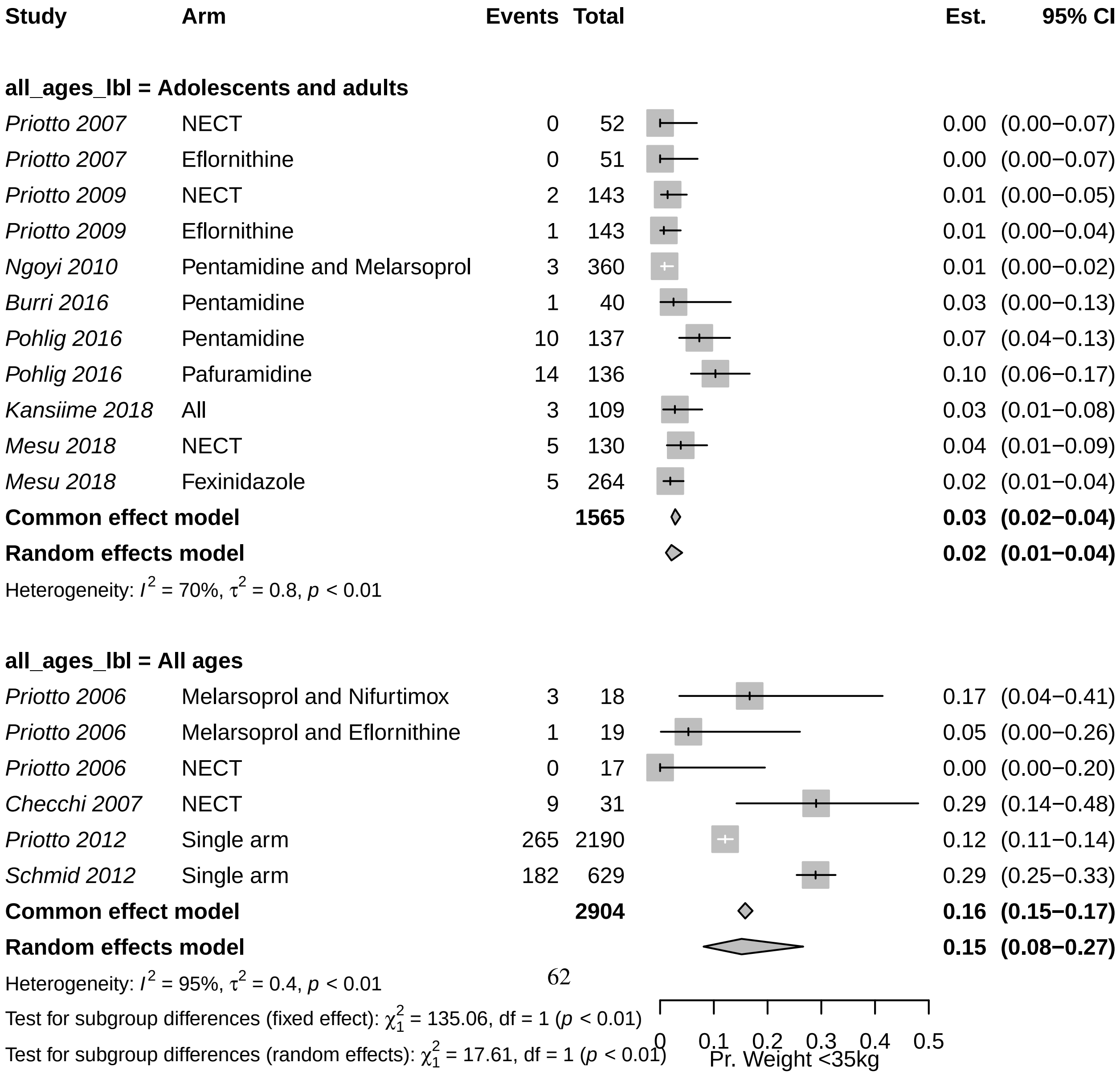

Because the data showed significant heterogeneity according to the tau-squared test for heterogeneity, we have chosen to use the random-effects estimate: 0.02 (0.01-0.04), represented by probability distribution: Beta(8.30, 359.61).

G.6.3 Proportion of S2 cases that are severe

- Name in the code: `treat_effect[["patient_char"]][["pc_s2_late]]`
- Source: [16, 41–47]
- Country of estimate: Various
- Statistical distribution and parameters: Beta(76.93, 44.87)
- Summary statistics (mean and 95% CI or fixed value): 0.63 (0.54, 0.72)

Notes

The definition of severe stage 2 gHAT disease by the WHO is when there are more than 100 white blood cells (WBC, leukocytes) per micro-liter in the cerebro-spinal fluid. We have searched the clinical trials for the proportion of stage 2 patients that have high concentrations of leukocytes upon admission to treatment.

Study	Arm	Events	Total	Est.	95% CI
Priotto 2006	Melarsoprol and Nifurtimox	9	18	0.50	(0.26−0.74)
Priotto 2006	Melarsoprol and Eflornithine	12	19	0.63	(0.38−0.84)
Priotto 2006	NECT	6	17	0.35	(0.14−0.62)
Eperon 2006	Melarsoprol	407	1108	0.37	(0.34−0.40)
Priotto 2007	NECT	31	52	0.60	(0.45−0.73)
Priotto 2007	Eflornithine	39	51	0.76	(0.63−0.87)
Checchi 2007	NECT	18	31	0.58	(0.39−0.75)
Priotto 2009	NECT	107	143	0.75	(0.67−0.82)
Priotto 2009	Eflornithine	115	143	0.80	(0.73−0.87)
Ngoyi 2010	Pentamidine and Melarsoprol	209	272	0.77	(0.71−0.82)
Priotto 2012	Single arm	1373	2190	0.63	(0.61−0.65)
Schmid 2012	Single arm	397	629	0.63	(0.59−0.67)
**Common effect model**			**4673**	**0.58**	**(0.57−0.60)**
**Random effects model**				**0.63**	**(0.55−0.71)**

Heterogeneity: $I^2 = 96\%$, $\tau^2 = 0.3$, $p < 0.01$

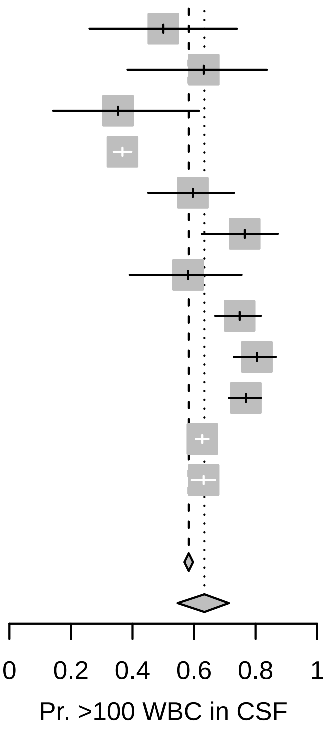

Because the data showed significant heterogeneity according to the tau-squared test for heterogeneity, we have chosen to use the random-effects combined estimate: 0.634 (0.546, 0.713), represented by the probability distribution Beta(76.93, 44.87).

G.6.4 Age of death from infection

- Name in the code: `treat_effect[["yld_yll"]][["aoi_s1s2_years]]`
- Source: [41–54]
- Country of estimate: DRC and South Sudan
- Statistical distribution and parameters: Gamma(148, 0.18)
- Summary statistics (mean and 95% CI or fixed value): 26.63 (22.41, 31.08)

Notes

Citation	Group	Age Group	Summary
Priotto 2006	Melarsoprol and Nifurtimox	All ages	Mean: 29.1 range: 5-56
Priotto 2006	Melarsoprol and Eflornithine	All ages	Mean: 28.1 range: 11-61
Priotto 2006	NECT	All ages	Mean: 29.1 range: 9-62
Balasegaram 2006	Pentamidine	All ages	148 under 15 and 504 over 15
Eperon 2006	Pentamidine	All ages	56 patients 0-5, 226 patients 6-15, 568 patients 15+
Eperon 2006	Melarsoprol	All ages	63 patients 0-5, 249 patients 6-15, 796 patients 15+
Priotto 2007	NECT	Over 15 years old	Mean: 33.1 range: 15-69
Priotto 2007	Eflornithine	Over 15 years old	Mean: 36.1 range: 15-70
Checchi 2007	NECT	All ages	Mean: 23.9 range: 4-45
Priotto 2009	NECT	Over 15 years old	Mean: 32.8 SD: 12.5
Priotto 2009	Eflornithine	Over 15 years old	Mean: 34.6 SD: 13.5
Ngoyi 2010	Pentamidine	Over 12 years old	Mean: 35 SD: 13
Ngoyi 2010	Pentamidine and Melarsoprol	Over 12 years old	Mean: 34 SD: 12
Priotto 2012	Single arm	All ages	Median: 24 IQR: 15-35
Schmid 2012	Single arm	All ages	35 patients 0-4 yo, 65 patients 5-11 yo, and 529 patients 12 yo or more.
Hasker 2012	All	All ages	Median: 27 IQR: 16-40
Alirol 2013	Single arm	All ages	Median: 36 IQR: 20-50
Burri 2016	Pentamidine	Over 15 years old and >35 kg	Median: 31 range: 15-50
Pohlig 2016	Pentamidine	Over 12 years old and >30 kg	Median: 31 range: 13-75
Pohlig 2016	Pafuramidine	Over 12 years old and >30 kg	Median: 30 range: 12-64
Kansiime 2018	NECT	Over 15 years old	Mean: 27.23 SD: 12.07
Kansiime 2018	Eflornithine	Over 15 years old	Mean: 27.33 SD: 8.59
Mesu 2018	NECT	Over 15 years old	Mean: 35.2 SD: 13.2
Mesu 2018	Fexinidazole	Over 15 years old	Mean: 34.5 SD: 12.6

No good registry of age of infection exists, so we have searched through the literature that we have used to parameterize the model for the average age of HAT patients. Among the studies that we have used to inform other parameters, eight studies reported age information in a sample of patients of all ages, and nine studies have reported the age information in a sample of older children or adults (12-15 years and older).

However, the data exists in a state that is difficult to synthesize:

Therefore, we have fit a gamma distribution to the means and medians of the studies that included patients of all ages. We have omitted the median from Alirol 2013 as this median seems unusually high – even higher than the mean age of patients in studies were only adults (over the age of 15) were recruited. Our distribution is therefore Gamma(147.93, 0.18) with a mean and 95% confidence interval of 27 (23, 31), which provides a sufficiently large bound of uncertainty in lieu of the information we have.

G.6.5 Length of hospital stay: NECT treatment

- Name in the code: `treat_effect[["length_hosp_stay"]][["lhs_s2_nect_days]]`
- Source: [51]
- Country of estimate: Global recommendations
- Statistical distribution and parameters: Fixed value
- Summary statistics (mean and 95% CI or fixed value): 10

Notes

For NECT treatment, patients must stay in inpatient care for a minimum of 7 days for the eflornithine infusions, and whether they stay for a total of 10 days for nifurtimox administration is unclear. For the most recent clinical trial [51], NECT patients were released on days 13-18 after admission, but we have assumed that for the most recent trial the average patient can be released from care after 10 days in the hospital.

G.6.6 Length of hospital stay: fexinidazole treatment

- Name in the code: `treat_effect[["length_hosp_stay"]][["lhs_s1s2_fexinidazole_days]]`
- Source: [51]
- Country of estimate: Global recommendations
- Statistical distribution and parameters: Fixed value
- Summary statistics (mean and 95% CI or fixed value): 10

Notes

For the only trial that is published [51], patients were released on days 13-18 after the initiation of treatment, although the treatment only took 10 days, so we have assumed that in routine care that the average patient will be in inpatient care for 10 days.

G.6.7 Pr. of relapse: pentamidine

- Name in the code: `treat_effect[["treatment_failure"]][["tf_prob_s1_pentamidine]]`
- Source: [41, 47–49, 52, 55]
- Country of estimate: Various
- Statistical distribution and parameters: Beta(50.3, 665.48)
- Summary statistics (mean and 95% CI or fixed value): 0.07 (0.05, 0.09)

Notes

The WHO guidelines for treatment of HAT in 2019 [16] presented existing data on treatment failure of pentamidine treatment. To produce one comprehensive estimate of treatment failure, we performed a meta-analysis on proportions within a single group.

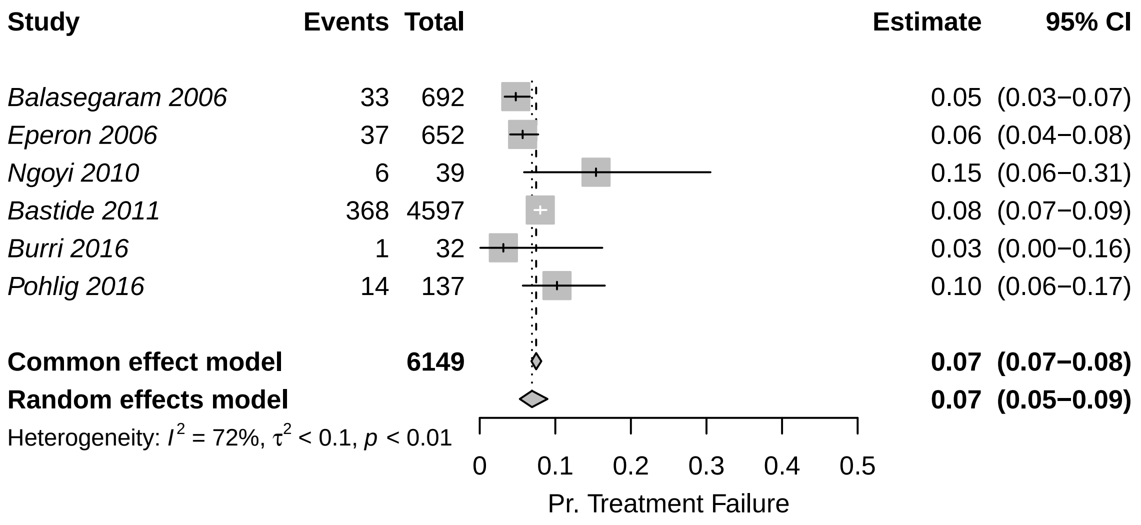

Because the data showed significant heterogeneity according to the tau-squared test for heterogeneity, we have chosen to use the random-effects estimate, to which we assigned a distribution of Beta(50.30, 665.47).

G.6.8 Pr. of relapse (treatment failure): NECT

- Name in the code: `treat_effect[["treatment_failure"]][["tf_prob_s2_nect]]`

- Source: [43–46, 50, 51]
- Country of estimate: Various
- Statistical distribution and parameters: Beta(15.87, 378.55)
- Summary statistics (mean and 95% CI or fixed value): 0.05 (0.02, 0.08)

Notes

The WHO guidelines for treatment of HAT in 2019 [16] presented existing data on treatment failure of NECT. Kansiime and colleagues [50] also performed a systematic review of studies estimating the outcomes of NECT treatment. To produce one comprehensive estimate of treatment failure, we performed a meta-analysis on proportions within single groups.

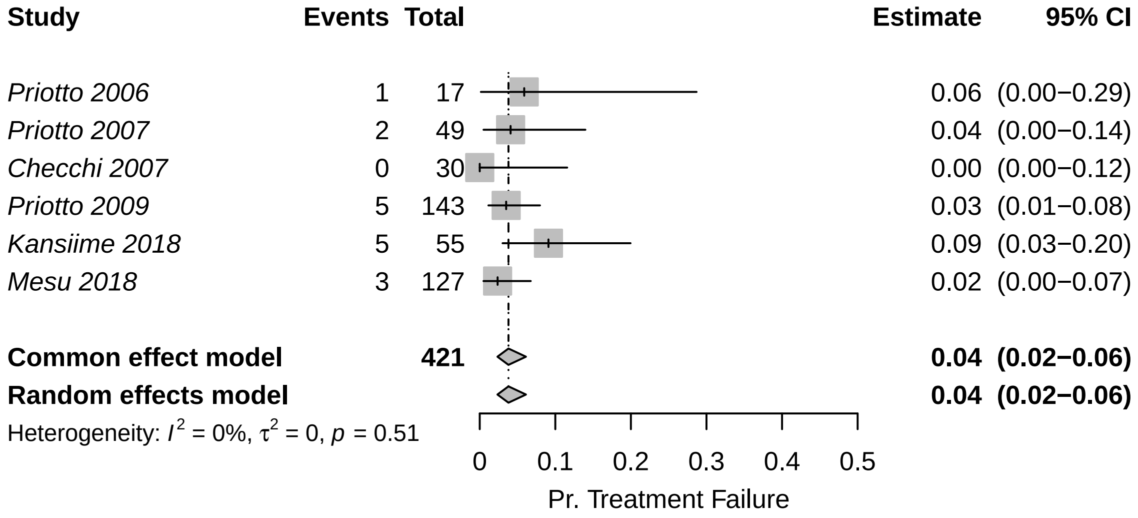

Because the data showed non-significant heterogeneity according to the tau-squared test for heterogeneity, we have chosen to use the fixed-effects estimate, to which we assigned a distribution of Beta(15.87, 378.55)

G.6.9 Pr. of relapse: fexinidazole

- Name in the code: `treat_effect[["treatment_failure"]][["tf_prob_s1s2_fexinidazole]]`
- Source: [16]
- Country of estimate: DRC
- Statistical distribution and parameters: Beta(9.49, 496.54)
- Summary statistics (mean and 95% CI or fixed value): 0.02 (<0.01, 0.03)

Notes

Mesu and colleagues [51] have published the only study on fexinidazole treatment effectiveness on late-stage 2 cases. Moreover, the accompanying meta-analysis for the WHO treatment guidelines released in 2019 show the outcomes an additional extension study on stage 1, both early and late-stage 2 disease as well we for the data from Mesu et al stratified by the concentration of WBC in the CSF [16].

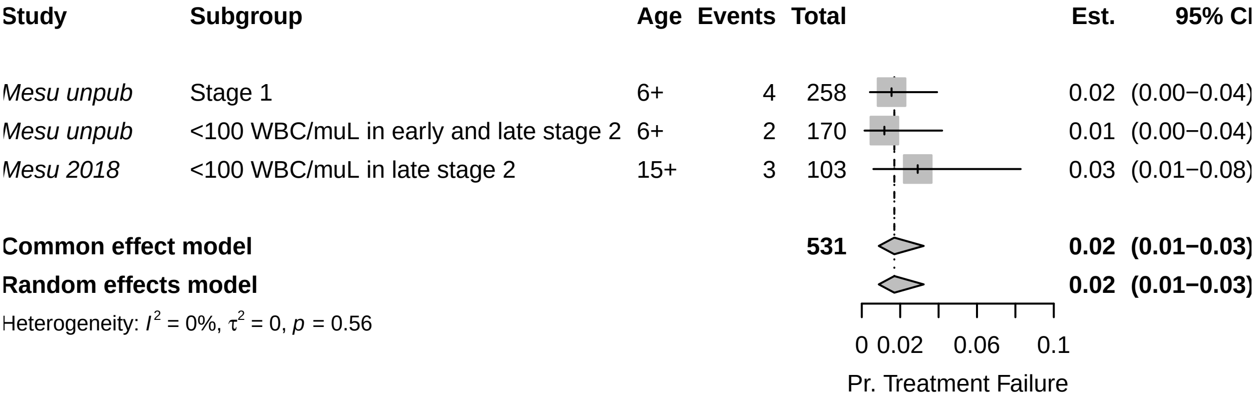

Because the data showed non-significant heterogeneity according to the tau-squared test for heterogeneity, we have chosen to use the fixed-effects combined estimate: 0.017 (0.009, 0.032), for which we assigned a distribution of Beta(9.49, 496.54).

G.6.10 SAE: pentamidine treatment

- Name in the code: `treat_effect[["sae"]][["sae_prob_s1_pentamidine]]`
- Source: [41, 48, 49]
- Country of estimate: DRC and South Sudan
- Statistical distribution and parameters: Beta(1.43, 551.42)
- Summary statistics (mean and 95% CI or fixed value): 0.002 (<0.001, 0.008)

Notes

As part of the WHO guidelines for treatment of HAT in 2019 ([16]), Cochrane performed a systematic review of studies that evaluated the efficacy of NECT compared to fexinidazole studies and presented the probability of serious adverse events. Severe or serious adverse events in studies for S1 treatment were defined as "significant hazard, contra-indication, side effect, or precaution" [41, 48, 49].

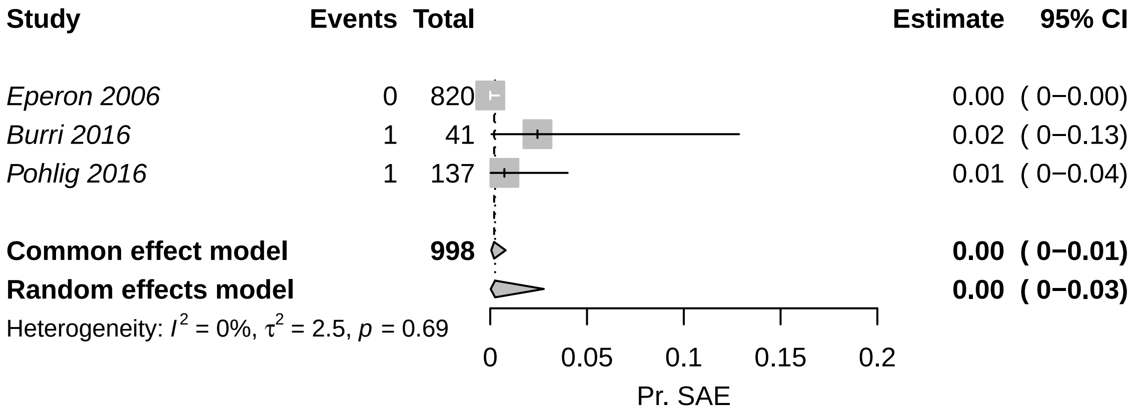

Because the results do not contain evidence of significant heterogeneity, we have chosen to use the fixed (pooled) estimate of 0 (0-0.01), which would result from a beta distribution of Beta(1.43, 551.42).

G.6.11 SAE: NECT treatment

- Name in the code: `treat_effect[["sae"]][["sae_prob_s2_nect]]`
- Source: [43–46, 50, 51]
- Country of estimate: DRC
- Statistical distribution and parameters: Beta(40.88, 367.8)
- Summary statistics (mean and 95% CI or fixed value): 0.05 (0.03, 0.08)

Notes

The WHO guidelines for treatment of gHAT in 2019 [16], presented all NECT studies to date, as did Kansiime and colleagues [50]. We searched through these studies for evidence of the probability of severe adverse vents (SAEs),descrived as events of Grade 3 or higher according to the National Cancer Institute Common Toxicity Criteria for Adverse Events (CTCAE).

To produce one comprehensive estimate of the probability of SAE, we performed a meta-analysis on proportions within single groups.

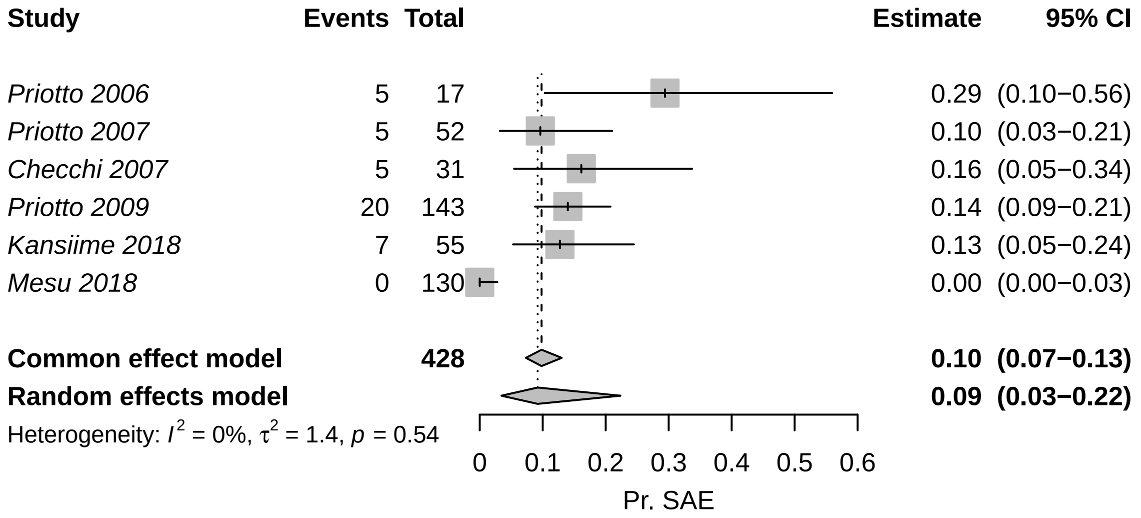

Because the data showed non-significant heterogeneity according to the tau-squared test for heterogeneity, we have chosen to use the fixed-effects estimate: 0.098 (0.073, 0.130), which would result from a beta distribution of Beta(40.88, 367.80).

G.6.12 SAE: fexinidazole treatment

- Name in the code: `treat_effect[["sae"]][["sae_prob_s1s2_fexinidazole]]`
- Source: [51]
- Country of estimate: DRC
- Statistical distribution and parameters: Beta(3, 261)
- Summary statistics (mean and 95% CI or fixed value): 0.01 (<0.01, 0.03)

Notes

There is only one published study on fexinidazole, so the probability of serious adverse events will be parameterized with the observations from that study: 4 adverse events attributable to fexinidazole in 3 people among 264 people, so we have assigned a distribution of Beta(3, 261).

There were additional data reported in the appendix to the WHO's interim guidelines ([16]) related to studies that are ongoing. However, since we do not know details about whether those SAEs were attributable to treatment, we have chosen to omit those data.

G.6.13 Days lost to disability: due to S1 disease

- Name in the code: `treat_effect[["yld_yll"]][["yld_s1_days]]`
- Source: [56, 57]
- Country of estimate: Uganda
- Statistical distribution and parameters: Gamma(21.89, 26.07)
- Summary statistics (mean and 95% CI or fixed value): 569.21 (355.50, 831.29)

Notes

Checchi and colleagues [58] reported a rate of progression from stage 1 to stage 2 disease of 0.0019 (0.0012, 0.0028), so we used the inverse of the confidence interval to come up with gamma parameters.

Our distribution is therefore Gamma(21.89, 26.07) with a mean and 95% confidence interval of 571.19 (353.13, 831.35), which provides a sufficiently large bound of uncertainty in light of the scarce information we have.

G.6.14 Days lost to disability: due to S2 disease

- Name in the code: `treat_effect[["yld_yll"]][["yld_s2_days]]`
- Source: [57]
- Country of estimate: Uganda
- Statistical distribution and parameters: Gamma(22.18, 12.38)
- Summary statistics (mean and 95% CI or fixed value): 275.57 (172.46, 401.20)

Notes

Checchi and colleagues [58] reported a rate of death from stage 2 disease of 0.0040 (0.0025, 0.0058), so used the inverse of the confidence interval to come up with gamma parameters.

Our distribution is therefore Gamma(22.18, 12.38) with a mean and 95% confidence interval of 273.73 (173.75, 396.68), which provides a sufficiently large bound of uncertainty in light of the scarce information we have.

G.6.15 Days lost to disability: due to SAE

- Name in the code: `treat_effect[["yld_yll"]][["sae_yld_s1s2_days]]`
- Source: [54]
- Country of estimate: DRC
- Statistical distribution and parameters: Gamma(1.22, 2.38)
- Summary statistics (mean and 95% CI or fixed value): 2.96 (0.14, 9.99)

Notes

Our only source of information for the duration of severe adverse events (SAEs) is Alirol 2013 [54], which lists the most common adverse events and the median duration of these events. Most envents last a median of 1-2 days (with interquartile ranges reaching up to 4 days).

For simplicity, we have fit a gamma distribution with interquartile range of 1-4 days. Our distribution is therefore Gamma(1.22, 2.38) with a mean and 95% confidence interval of 2.92 (0.12, 9.95), which provides a sufficiently large range of values in light of the scarce information we have.

G.7 Life-years lost (DALY) parameters

G.7.1 Life expectancy

- Name in the code: `disability[["le_years"]]`
- Source: [21]
- Country of estimate: DRC
- Statistical distribution and parameters: Fixed value
- Summary statistics (mean and 95% CI or fixed value): 60.02

Notes

We retrieved the life expectancy at birth from the World Bank's World Development Index [21]. As this should be quite accurate we have decided to enter a fixed estimate into the model.

G.7.2 Disability weights: S1 disease

- Name in the code: `disability[["dw_s1"]]`
- Source: [22]
- Country of estimate: GBD
- Statistical distribution and parameters: Beta(22.96, 147.21)
- Summary statistics (mean and 95% CI or fixed value): 0.14 (0.09, 0.19)

Notes

The Global Burden of Disease listed the impact of sleeping sickness as equivalent to the health state labeled "Motor plus cognitive impairments, severe" and their estimate for a disability weight is 0.542 (0.374-0.702), using the 2013 weight values. No distinction was made between stage 1 and 2 of the disease.

While this seems appropriate for the second stage of sleeping sickness, for stage 1 disability we chose to use the disability weights for equivalent to "infectious disease, acute episode, severe", which is described as "has a high fever and pain, and feels very weak, which causes great difficulty with daily activities" and has a much lower disability weight equivalent to 0.133 (0.088-0.190). The distribution for this parameter is therefore Beta(22.96, 147.21).

It should be noted that other cost-effectiveness analyses have used different values for disability weights [9, 59, 60]. These values arise from the 1994 Global Burden of Disease Study but we prefer to consider updated values. Since most of the disability is due to deaths rather than illness during life, we do not believe that this difference is cause for concern.

G.7.3 Disability weights: S2 disease

- Name in the code: `disability[["dw_s2"]]`
- Source: [22]
- Country of estimate: GBD
- Statistical distribution and parameters: Beta(18.37, 15.63)
- Summary statistics (mean and 95% CI or fixed value): 0.54 (0.37, 0.70)

Notes

The Global Burden of Disease listed the impact of sleeping sickness as equivalent to the health state labeled "Motor plus cognitive impairments, severe" and their estimate for a disability weight is 0.542 (0.374-0.702), using the 2013 weight values. No distinction was made between stage 1 and 2 of the disease. The distribution for the parameter is Beta(18.37, 15.63).

It should be noted that other cost-effectiveness analyses have used different values for disability weights [9, 59, 60]. These values arise from the 1994 Global Burden of Disease Study but we prefer to consider updated values. Since most of the disability is due to deaths rather than illness during life, we do not believe that this difference is cause for concern.

G.7.4 Disability weights: SAE

- Name in the code: `disability[["sae_dw_s1s2"]]`
- Source: [22]
- Country of estimate: GBD
- Statistical distribution and parameters: Uniform(0.04, 0.11)
- Summary statistics (mean and 95% CI or fixed value): 0.08 (0.04, 0.11)

Notes

As far as we are aware, no one has considered the disability due to severe adverse events attributable to gHAT treatment, but the most common adverse events are gastrointestinal problems and headaches.

We consulted the Global Burden of Disease for disability weights. The health state labeled "symptomatic tension-type headache" was described as "moderate headache that also affects the neck, which causes difficulty in daily activities" and was estimated to have a disability weight equal to 0.037 (0.022–0.057). The health state labeled "moderate symptomatic gastritis and duodenitis without anemia" was described as "abdominopelvic problem, moderate has pain in the belly and feels nauseous; the person has difficulties with daily activities" and and was estimated to have a disability weight equal to 0.114 (0.078–0.159).

Our distribution is therefore Uniform 0.037-0.114. Since most of the disability is due to deaths rather than illness during life, we do not believe that the uncertainty in this parameter is cause for concern for the purpose of the conclusions of this analysis.

G.8 Vector control parameters

G.8.1 Linear km of targets

- Name in the code: `vector_effect[["vc_km"]]`
- Source: [15]
- Country of estimate: DRC
- Statistical distribution and parameters: Fixed value
- Summary statistics (mean and 95% CI or fixed value): Varies by health zone

Notes

The parameter for the extent of riverbank covered by vector targets is a fixed value. The values for each health zone are detailed in the Supplementary Methods, section A.2.1 and sensitivity analysis were described in Supplementary Methods, section A.6.

G.8.2 Target per km

- Name in the code: `vector_effect[["vc_units"]]`
- Source: [15]
- Country of estimate: DRC and Uganda
- Statistical distribution and parameters: Fixed value
- Summary statistics (mean and 95% CI or fixed value): Varies by health zone

Notes

We used a fixed value for the parameter for the extent of riverbank covered by vector targets. The values for each health zone are detailed in the Supplementary Methods, section A.2.1 and sensitivity analysis were described in Supplementary Methods, section A.6.

G.8.3 Replacement rate of targets per year

- Name in the code: `vector_effect[["vc_deployments_yr"]]`
- Source: [14, 15]
- Country of estimate: DRC
- Statistical distribution and parameters: Fixed value
- Summary statistics (mean and 95% CI or fixed value): 2

Notes

We have set this parameter as a fixed number, as this is the number of times that one must replace a set of targets in order to provide continuous protection throughout the year [14, 15].

G.9 Screening cost parameters

G.9.1 AS: capital costs of a team

- Name in the code: `surv_cost[["as_capital"]]`
- Source: [8–10]
- Country of estimate: DRC: Mosango and Yasa Bonga
- Distribution and parameters: Gamma(81.02, 114.54)
- Summary statistics (mean and 95% CI or fixed value): 9,276 (7,378, 11,375)

Notes

Capital costs are denominated in 2018 USD.

To our knowledge, no active screening costs have been estimated via detailed costing study [61]. We took into account only the two studies that calculated costs using an ingredients approach [8, 9].

Lutumba and colleagues [8] calculated that the total cost of screening 40,000 patients in 2003 was 46,734.29 Euros (1 Euro = 0.86 USD in 2003) (see table 3 of [8]). Of that value, 21 percent of the costs were capital costs, or a cost of 14502.69 in 2018 USD. The publication did not indicate whether that value was annualized or not.

According to Bessel and colleagues [9] the cost for a mobile team that screens 250 patients per day for 220 days a year has capital investments (annualized for five years) of $12,000 for a team that administers CATT and $12,781 for a team that administers RDT (in 2013 USD) (see [9] Table S1). Adjusting for inflation, that would equal 13002.59 in 2018 USD.

According to Snijders et al [10], the capital cost of an active screening team was $11,406 in 2018 for a team that administer CATT.

Although no publication gave us a sense of the uncertainty in capital costs, but since we had three studies, we assumed that the range was equivalent to the 95% confidence interval. Therefore, our probability distribution is Gamma(199999.99, 0.07), which yields a distribution with mean and confidence intervals of 13,060 (13,002, 13,116).

G.9.2 AS: fixed management costs of a team

- Name in the code: `surv_effect[["as_recurrent"]]`
- Source: [8–10]
- Country of estimate: DRC: Mosango and Yasa Bonga
- Distribution and parameters: Gamma(63.31, 630.94)
- Summary statistics (mean and 95% CI or fixed value): 39,955 (30,845, 50,435)

Notes

Recurrent management costs are denominated in 2018 USD.

To our knowledge, no active screening costs have been estimated via detailed costing study [61]. We took into account only the two studies that calculated costs using an ingredients approach [8–10].

Lutumba and colleagues [8] calculated that the total cost of screening 40,000 patients in 2003 was 46,734.29 Euros (1 Euro = 0.86 USD in 2003, for an equivalent of $40,191) (see table 3 of [8]). Of that value, 79 percent of the costs were recurrent fixed costs ($31,751), or a cost of 54,557.73 in 2018 USD after adjusting for inflation and changes in the exchange rate.

According to Bessel and colleagues [9] the cost for a mobile team that screens 250 patients per day for 220 days a year has annual recurrent costs of $30,307 and daily recurrent costs of $97 in 2013 values (see [9] Table S1). Summing those costs ($51,647) and adjusting for inflation and changes in the exchange rate, that would equal 54,198.00 in 2018 USD.

According to Snijders and colleagues [10], the management and recurrent costs of an active screening team were $42,408 in 2018, and $7,961 for the management costs from the provincial and central level PNLTHA (including training and supervision) for a total of 50,369.

Although no publication gave us a sense of the uncertainty in recurrent costs, but since we had at least three studies, we assumed that the range of observations was equal to the 95% confidence interval (54,198- 50,359). Therefore, our probability distribution is Gamma(199999.99, 0.27), which yields a distribution with mean and confidence intervals of 54,438 (54,193, 54,679).

G.9.3 CATT algorithm: cost per test used

- Name in the code: `surv_cost[["dx_catt"]]`
- Source: [8–10, 61]
- Country of estimate: DRC
- Distribution and parameters: Gamma(25.19, 0.02)
- Summary statistics (mean and 95% CI or fixed value): 0.52 (0.34, 0.75)

Notes

To our knowledge, costs for CATT tests are only featured in four sources:

1) Keating et al [61] listed the cost of a CATT test at 0.73 in 1998 USD, equivalent to 0.59 in 2018 USD.
2) Lutumba et al [62] listed the cost of a CATT test at 0.52 in 2003 USD, equivalent to 0.66 in 2018 USD.
3) Bessel et al [9] listed the cost of CATT test at 0.70 for a mobile team and 0.89 for a fixed post in 2013 USD. The reason for the difference in price between screening at a mobile team vs screening at a fixed post was due to the differential wastage and was given in the supplement notes: “Costs at mobile teams and fixed units are different because once a bottle of CATT antigen is open repeat cases and controls must be performed. Materials are the

cost of test materials plus the cost of the lancet." In terms of 2018 USD, the costs are 0.73 for CATT tests in mobile teams 0.93 for CATT tests in fixed posts. We take into account wastage as separate parameter.

4) Snijders et al listed the cost of a CATT test at 0.74 in terms of 2018 USD [10].

Because we had more than two studies on this parameter, we chose gamma distributions where the 2.5th and the 97.5th percentiles would match the minimum and maximum values in the literature, so our parameter distribution is Gamma(75.18, 0.01), which has a mean and confidence interval of 0.75 (0.59, 0.93).

Considering delivery costs (described in Supplementary Note 4: Parameter Glossary, section G.10.6), the cost of CATT tests are 1.06 (0.83, 1.33) in 2018 USD. Considering both delivery costs and wastage, the cost of CATT tests are 1.18 (0.92, 1.48) in active screening teams and 1.37 (1.06, 1.73) in passive screening (fixed) posts.

G.9.4 Staging: lumbar puncture & lab exam

- Name in the code: `surv_cost[["dx_lp_lab"]]`
- Source: [10, 63]
- Country of estimate: DRC and Chad
- Distribution and parameters: Gamma(2.42, 3.66)
- Summary statistics (mean and 95% CI or fixed value): 8.90 (1.45, 23.20)

Notes

To our knowledge, costs for lumbar puncture tests were listed in detail only by Bessel and colleagues [9] and by Snijders and colleagues [10]. The cost listed by Bessel et al was 2.38 in 2013 USD, equivalent to 2.50 in 2018 USD. Snijders and colleagues report a cost of 23.02 in 2018 USD. Irurzun-Lopez [63] have reported a similar value for screening of meningitis in Chad, so we will assume a value equal to that of Snijders and colleages.

Because we had more than two studies to inform this parameter, we chose gamma distributions where the 2.5th and the 97.5th percentiles would match the minimum and maximum values in the literature, so our parameter distribution is Gamma(3.58, 2.83), which yields a distribution with mean and confidence intervals of 10.12 (2.48, 22.85).

G.9.5 Confirmation: microscopy

- Name in the code: `surv_cost[["dx_microscopy1"]]`
- Source: [12]
- Country of estimate: DRC: Mosango and Yasa Bonga
- Distribution and parameters: Gamma(8.47, 1.27)
- Summary statistics (mean and 95% CI or fixed value): 10.68 (4.70, 18.84)

Notes

The per-patient price to confirm a patient with a full microscopy procedure is reported in an upcoming publication by Snijders et al [12]. They report that a microscopy procedure constituted of mAECT and LNA (lymph node aspiration) costs 9.53 in 2018 USD. Because we had no report of the standard error around that estimate, we assigned a gamma distribution that had a confidence interval that spanned half the estimate and twice the estimate, yielding a distribution of Gamma(8.47, 1.27).

G.9.6 RDT algorithm: costs per test used

- Name in the code: `surv_cost[["dx_rdt"]]`
- Source: [12]
- Country of estimate: DRC
- Distribution and parameters: Gamma(8.47, 0.19)
- Summary statistics (mean and 95% CI or fixed value): 1.60 (0.71, 2.83)

Notes

To our knowledge, costs for RDT tests were listed in Sutherland et al [60], Bessel et al [9], and Snijders et al [12].

1) Both Sutherland et. al and Bessel et. al coincided on a cost of 0.50 USD 2013 for the test in the international market (after a 25 cent subsidy paid for outside of DRC). Bessel also calculated staff costs, shipment, and wastage for 0.32 USD 2013. Because we can split the cost between tradable and non-tradable costs, we inflate and adjust the costs for the staff costs and shipment and then add the cost of the RDT. In terms of 2018 USD costs, the shipment and the staff costs are 0.34, and the total cost is 1.09.

2) Snijders et. al reported a cost between 0.85 and 1.97, depending on the company from which the test is purchased. It should be noted that no single company produces enough tests for any single intervention, and so a range of prices must be considered. Snijders' estimate does not include delivery or wastage, so we take that into account separately.

It appears difficult to compare the three estimates, so we will take Snijders' estimate from a recent micro-costing analysis. The mean was $1.58, and we assign a distribution with confidence intervals equal to half and double the costs. The distribution is Gamma(8.47, 0.19).

Considering delivery costs (described in Supplementary Note 4: Parameter Glossary, section G.10.6), the cost of RDT tests are 2.30 (1.03, 4.11) in 2018 USD. Considering both delivery costs and wastage, the cost is 2.46 (1.10, 4.36) in passive screening teams, and we do not consider the deployment of RDTs in active screening teams.

G.9.7 Variable management costs (PNLTHA mark-up)

- Name in the code: `surv_cost[["pnltha_mu"]]`
- Source: [12]
- Country of estimate: DRC
- Distribution and parameters: Uniform(0.1, 0.2)
- Summary statistics (mean and 95% CI or fixed value): 0.15 (0.10, 0.20)

Notes

Snijder's and colleagues [12] have assumed that there is a component of management at the national program (PNLTHA) level that is approximately 15% of the expenses at the local and coordination level (both fixed costs and variable/consumable costs for both active screening and passive screening in fixed health posts). However, the mark-up was not applied to the consult in the fixed health post, as these were consultations paid for by patients for symptoms in general, but testing and confirmation for HAT specifically is administered and paid for by PNLTHA, so we have included a mark-up for these items.

G.9.8 PS: capital costs of a facility

- Name in the code: `surv_effect[["ps_capital"]]`
- Source: [12]
- Country of estimate: DRC: Mosango and Yasa Bonga
- Distribution and parameters: Gamma(8.47, 209.8)
- Summary statistics (mean and 95% CI or fixed value): 1,777 (778, 3,157)

Notes

Capital costs are denominated in 2018 USD and apply to each health center or hospital that is capable of HAT diagnosis.

To our knowledge, no passive screening costs have been estimated via detailed costing study [61]. We took into account the upcoming results of a micro-costing study in Yasa Bonga and Mosango [12], two health zones of the former Bandundu province and in the current study.

To parameterize the model, we assign a distribution with 95% confidence intervals equal to half and double the costs. The distribution is Gamma(8.47, 209.80), which yields a distribution with median and confidence intervals of 1,712 (796, 3,156).

G.9.9 PS: fixed recurrent management costs

- Name in the code: `surv_effect[["ps_recurrent"]]`
- Source: [12]

- Country of estimate: DRC: Mosango and Yasa Bonga
- Distribution and parameters: Gamma(8.47, 985.55)
- Summary statistics (mean and 95% CI or fixed value): 8,368 (3,743, 14,965)

Notes

Recurrent management costs are denominated in 2018 USD and apply to health health zone.

To our knowledge, no passive screening costs have been estimated via detailed costing study [61]. We took into account the upcoming results of a micro-costing study in Yasa Bonga and Mosango [12], two health zones of the former Bandundu province and two health zones of the current study. The value was 7422 in 2018 USD.

To parameterize the model, we assign a distribution with confidence intervals equal to half and double the costs. The distribution is Gamma(8.47, 985.55), which yields a distribution with median and confidence intervals of 8,063 (3,724, 14,898).

G.10 Treatment cost parameters

G.10.1 Hospital stay: cost per day

- Name in the code: `treat_cost[["ip_day"]]`
- Source: [19, 64, 65]
- Country of estimate: DRC
- Distribution and parameters: Gamma(5.81, 0.24)
- Summary statistics (mean and 95% CI or fixed value): 1.39 (0.50, 2.71)

Notes

We got the estimates of inpatient treatment costs from the 2010 WHO CHOICE cost estimates (recently updated by [64] and [65]). In 2010, a consult at a primary hospital in DRC would be 2.41 (0.90, 5.73) I$. In 2010, a consult at a secondary hospital in DRC would be 2.59 (0.98, 5.81) I$, and a consult at a tertiary hospital in DRC would be 3.25 (1.32, 7.20) I$.

The equivalent estimates in 2010 USD are 1.27 (0.47, 3.02) per day at a primary hospital, 1.36 (0.52, 3.06) per day at a secondary hospital, and 1.71 (0.70, 3.79) per day at a tertiary hospital. After converting to local currency, applying the inflation index, and converting to 2018 USD, the estimates are 1.67 (0.63, 3.98) per day at a primary hospital, 1.80 (0.68, 4.04) per day at a secondary hospital, and 2.26 (0.92, 5.00) per day at a tertiary hospital.

At the moment, we do not know how many of each kind of hospital the population of HAT patients attend, nor do we understand how costs at district hospitals, referral hospitals, etc resemble those of the two kinds of hospitals under analysis by the WHO CHOICE program. Therefore, we take the estimate with a higher mean (hospital day in a tertiary hospital) in an effort not to under-state the costs of treatment and interventions.

To parameterize the model, we assign a gamma distribution with 95% confidence intervals equal to those reported by WHO CHOICE [19]. The distribution is Gamma(5.81, 0.44), which yields a distribution with median and confidence intervals of 2.55 (0.93, 4.96). Although this yields a higher mean, the uncertainty is adequately characterized.

G.10.2 Outpatient consultation: cost

- Name in the code: `treat_cost[["op"]]`
- Source: [12, 66]
- Country of estimate: DRC
- Distribution and parameters: Uniform(1.37, 3.33)
- Summary statistics (mean and 95% CI or fixed value): 2.34 (1.42, 3.28)

Notes

We got the estimates of outpatient consultation costs from two sources:

1) Laokri and colleagues [66] presented an estimate with mean $2.33 and standard deviation 0.27 in 2013 values, or 1.38 in 2018 values.
2) Snijders and colleagues [**Snijders2020b**] reported that the cost of a consultation is $3.33 in 2018 values.

To parameterize the model, we assign a distribution: Uniform(1.37, 3.33).

G.10.3 Course of pentamidine: cost

- Name in the code: `treat_cost[["rx_pentamidine"]]`
- Source: [61]
- Country of estimate: WHO
- Distribution and parameters: Fixed value
- Summary statistics (mean and 95% CI or fixed value): 54

Notes

The cost of pentamidine, for stage 1 disease. Because it is available on the international market, where is it sold in USD, and not subject to the inflationary pressures of any particular country, we have not inflated the cost or converted them to any other currency.

In the future pentamidine treatment it may be replaced with fexinidozole treatment, which would circumvent the need for a lumbar puncture.

G.10.4 Course of NECT: cost

- Name in the code: `treat_cost[["rx_nect"]]`
- Source: [67]
- Country of estimate: WHO
- Distribution and parameters: Fixed value
- Summary statistics (mean and 95% CI or fixed value): 460

Notes

This represents the cost of NECT to the capital for stage 2 disease. Simarro and colleagues listed a cost of 1440 USD for treatment of four patients.

Because it is available on the international market, where is it sold in USD, and not subject to the inflationary pressures of any particular country, we have not inflated the cost or converted them to any other currency.

In the future it may be replaced with fexinidozole and this would be the drug for treatment failures or very severe patients.

G.10.5 Course of fexinidazole: cost

- Name in the code: `treat_cost[["rx_fexinidazole"]]`
- Source: [60]
- Country of estimate: WHO
- Distribution and parameters: Fixed value
- Summary statistics (mean and 95% CI or fixed value): 50

Notes

The cost of fexanidozole, for stage 1 and 2 disease. In the near future this will be the drug of choice for first-line treatment for both stages of disease. It may require hospitalization, but eventually it should be taken on an outpatient basis.

G.10.6 Drug delivery mark-up

- Name in the code: `treat_cost[["rx_delivery"]]`
- Source: [64, 65]
- Country of estimate: DRC
- Distribution and parameters: Beta(45, 55)
- Summary statistics (mean and 95% CI or fixed value): 0.45 (0.35, 0.55)

Notes

Because we do not know the delivery price of drugs for each country, we have applied the standard value for the mark up of traded goods recommended by the WHO CHOICE program for AFRO E: https://www.who.int/teams/health-systems-governance-and-financing/economic-analysis/costing-and-technical-efficiency/quantities-and-unit-prices-(cost-inputs).

G.11 Vector control cost parameters

G.11.1 Operational cost per kilometer of riverbank covered

- Name in the code: `vector_cost[["vc_operation"]]`
- Source: [23]
- Country of estimate: Uganda
- Distribution and parameters: Gamma(8.47, 14.17)
- Summary statistics (mean and 95% CI or fixed value): 120.28 (53.33, 212.26)

Notes

Vectoef control operational costs are denominated in 2018 US dollars on a per-kilometre basis.

To our knowledge, only one vector control micro-costing study has been performed by Shaw and colleagues [23]. In that study, centred in Arua, Uganda, 1551 targets were laid out at 20 targets per kilometre on the river banks [13], for a total of 77.55 kilometers. The total cost of vector control operations for one year was 21,337 in 2013 USD, but it included a deployment and a target maintenance mission for 4,290 USD. Further, the target deployment itself cost 7370 USD. Therefore, the cost of the operation excluding target deployment was 9,677 USD per year, or 124.79 USD per kilometre in 2013 USD in Uganda.

To convert those Uganda costs to DRC costs in 2018 (as most costs were in non-tradeable goods and labor), we turn the USD into Uganda's local currency in 2013. We can take Uganda's local currency and convert that value to PPP (international dollars) values using the PPP conversion factor (local currency units per international dollar) for Uganda. Then we took the operational costs in international dollars and convert them to local currency in DRC with the PPP conversion factor for DRC. Next, we use the CPI to inflate costs to 2018 levels and then use the exchange rate with USD to get 2018 USD values. The equivalent cost for DRC is 188.22 USD per kilometre per year.

As there was no sense of the uncertainty in target deployment costs, we assign a distribution with confidence intervals equal to half and double the costs. The distribution is Gamma(8.47, 24.99), which yields a distribution with mean and confidence intervals of 212.26 (93.20, 377.91).

G.11.2 Deployment cost per target

- Name in the code: `vector_cost[["vc_deploy"]]`
- Source: [23]
- Country of estimate: Uganda
- Distribution and parameters: Gamma(8.47, 0.54)
- Summary statistics (mean and 95% CI or fixed value): 4.57 (2.02, 8.26)

Notes

Target deployment costs are denominated in 2018 US dollars on a per-target basis.

To our knowledge, only one vector control micro-costing study has been performed by Shaw and colleagues [23]. In that study, centred in Arua, Uganda, 1,551 targets were laid out. The target deployment activities cost 7,370 USD, or per target 4.75 USD (in 2013 values).

To convert those Uganda costs to DRC costs in 2018 (as most costs were in non-tradeable goods and labour), we turn the USD into Uganda's local currency in 2013. We can take Uganda's local currency and convert that value to PPP (international dollars) values using the PPP conversion factor (local currency units per international dollar) for Uganda. Then we took the operational costs in international dollars and convert them to local currency in DRC with the PPP conversion factor for DRC. Then, we use the CPI to inflate costs to 2018 levels and then use the exchange rate with USD to get 2018 USD values. The equivalent cost for DRC is 7.17 USD per target.

Uncertainty: as there was no sense of the uncertainty in target deployment costs, we assign a distribution with confidence intervals equal to half and double the costs. The distribution is Gamma(8.47, 0.95), which yields a distribution with mean and confidence intervals of 8.03 (3.61, 14.32).

H Supplementary References

1. Crump, R. E. *et al.* Quantifying epidemiological drivers of gambiense human African Trypanosomiasis across the Democratic Republic of Congo. *PLOS Computational Biology* **17** (ed Perkins, A.) e1008532. ISSN: 1553-7358. https://dx.plos.org/10.1371/journal.pcbi.1008532 (Jan. 2021).
2. Huang, C. I. *et al. Identifying regions for enhanced control of gambiense sleeping sickness in the Democratic Republic of Congo* 2020. https://www.medrxiv.org/content/10.1101/2020.07.03.20145847v2.
3. Rock, K. S., Torr, S. J., Lumbala, C. & Keeling, M. J. Quantitative evaluation of the strategy to eliminate human African trypanosomiasis in the Democratic Republic of Congo. *Parasites and Vectors* **8,** 1–13. ISSN: 17563305. http://dx.doi.org/10.1186/s13071-015-1131-8 (2015).
4. Mahamat, M. H. *et al.* Adding tsetse control to medical activities contributes to decreasing transmission of sleeping sickness in the Mandoul focus (Chad). *PLOS Neglected Tropical Diseases* **11,** 1–19. ISSN: 19352735 (2017).
5. Dowdle, W. R. The principles of disease elimination and eradication. *Bulletin of the World Health Organization* **76,** 22–25. ISSN: 00429686 (1998).
6. WHO Expert Committee on human African trypanosomiasis. *Control and surveillance of human African trypanosomiasis: report of a WHO expert committee* tech. rep. (2013), 237. https://www.who.int/trypanosomiasis_african/surveillance/en/.
7. World Health Organization. *Report from the third WHO stakeholders meeting on elimination of gambiense human African trypanosomiasis elimination* tech. rep. (World Health Organization, Geneva, Switzerland, 2020), vii, 67. https://www.who.int/trypanosomiasis_african/resources/9789240002296/en/.
8. Lutumba, P. *et al.* Cost-effectiveness of Algorithms for Confirmation Test of Human African Trypanosomiasis. *Emerging Infectious Diseases* **13,** 1484–1490. ISSN: 1080-6040. http://wwwnc.cdc.gov/eid/article/13/10/06-0358_article.htm (Oct. 2007).
9. Bessell, P. R. *et al.* Cost-effectiveness of using a rapid diagnostic test to screen for human African trypanosomiasis in the Democratic Republic of the Congo. *PLOS ONE* **13** (ed Arez, A. P.) e0204335. ISSN: 1932-6203. https://dx.plos.org/10.1371/journal.pone.0204335 (Sept. 2018).
10. Snijders, R. *et al.* Cost of a new method of active screening for human African trypanosomiasis in the Democratic Republic of the Congo. *PLOS Neglected Tropical Diseases* **14** (ed Ndung'u, J. M.) e0008832. ISSN: 1935-2735. https://dx.plos.org/10.1371/journal.pntd.0008832 (Dec. 2020).
11. Simarro, P. P. *et al.* Mapping the capacities of fixed health facilities to cover people at risk of gambiense human African trypanosomiasis. *International Journal of Health Geographics* **13.** ISSN: 1476072X (2014).
12. Snijders, R. *et al.* Costs and outcomes of integrated human African trypanosomiasis surveillance system using rapid diagnostic tests, Democratic Republic of the Congo. *Emerging Infectious Diseases* **27,** 2144–2153. ISSN: 10806059 (2021).
13. Tirados, I. *et al.* Tsetse Control and Gambian Sleeping Sickness; Implications for Control Strategy. *PLOS Neglected Tropical Diseases* **9** (ed Dumonteil, E.) e0003822. ISSN: 1935-2735. https://dx.plos.org/10.1371/journal.pntd.0003822 (Aug. 2015).
14. Liverpool School of Tropical Medicine. *Use of Tiny Targets to control tsetse flies in Gambian HAT foci: standard operating procedures* tech. rep. October (2016).
15. Tirados, I. *et al.* Impact of tiny targets on Glossina fuscipes quanzensis, the primary vector of human African trypanosomiasis in the Democratic Republic of the Congo. *PLOS Neglected Tropical Diseases* **14** (ed Matovu, E.) e0008270. ISSN: 1935-2735. https://dx.plos.org/10.1371/journal.pntd.0008270 (Oct. 2020).
16. WHO Department of Control of Neglected Tropical Diseases. *WHO interim guidelines for the treatment of gambiense human African trypanosomiasis* tech. rep. (World Health Organization, Geneva, Switzerland, 2019). https://www.who.int/trypanosomiasis_african/resources/9789241550567/en/.
17. Claxton, K. P. *et al. The Gates Reference Case for Economic Evaluation* tech. rep. April (The Bill and Melinda Gates Foundation, 2014), 1–68. https://pure.york.ac.uk/portal/en/publications/the-gates-reference-case-for-economic-evaluation(2663ebb2-e102-48c1-af2f-3a482a2eaae0)/export.html.

18. Murray, C. J. Quantifying the burden of disease: The technical basis for disability-adjusted life years. *Bulletin of the World Health Organization* **72,** 429–445. ISSN: 00429686 (1994).

19. World Health Organization. *Making Choices in Health: WHO guide to cost-effectiveness analysis* (eds Edejer, T. T.-T. *et al.*) ISBN: 92 4 154601 8 (Geneva, Switzerland, 2003).

20. Rushby, J. A. & Hanson, K. Calculating and presenting disability adjusted life years (DALYs) in cost-effectiveness analysis. *Health Policy and Planning* **16,** 326–331. ISSN: 14602237. `https://academic.oup.com/heapol/article-lookup/doi/10.1093/heapol/16.3.326` (Sept. 2001).

21. World Bank. *Life expectancy at birth, total (years)* Washington, DC., 2020. `https://data.worldbank.org/indicator/SP.DYN.LE00.IN?locations=CD`.

22. GBD 2017 DALYs and HALE Collaborators. Global, regional, and national disability-adjusted life-years (DALYs) for 359 diseases and injuries and healthy life expectancy (HALE) for 195 countries and territories, 1990-2017: A systematic analysis for the Global Burden of Disease Study 2017. *The Lancet* **392,** 1859–1922. ISSN: 1474547X (2018).

23. Shaw, A. P. *et al.* Costs Of Using "Tiny Targets" to Control Glossina fuscipes fuscipes, a Vector of Gambiense Sleeping Sickness in Arua District of Uganda. *PLOS Neglected Tropical Diseases* **9,** 1–19. ISSN: 19352735 (2015).

24. Johns, B., Baltussen, R. & Hutubessy, R. Programme costs in the economic evaluation of health interventions. *Cost Effectiveness and Resource Allocation* **1,** 1. ISSN: 1478-7547. `http://www.pubmedcentral.nih.gov/articlerender.fcgi?artid=156020&tool=pmcentrez&rendertype=abstract` (Feb. 2003).

25. Drummond, M. F., Sculpher, M. J., Claxton, K., Stoddart, G. L. & Torrance, G. W. *Methods for the Economic Evaluation of Health Care Programmes* (Oxford University Press, Oxford, UK, 2015).

26. Briggs, A., Claxton, K. & Sculpher, M. *Decision Modelling for Health Economic Evaluation* First (Oxford University Press, Oxford, UK, 2006).

27. Stinnett, A. A. & Mullahy, J. Net Health Benefits: A New Framework for the Analysis of Uncertainty in Cost-Effectiveness Analysis. *Medical Decision Making* **18,** S68–S80. ISSN: 0272-989X. `http://mdm.sagepub.com/cgi/doi/10.1177/0272989X9801800209` (Apr. 1998).

28. Barton, G. R., Briggs, A. H. & Fenwick, E. A. Optimal cost-effectiveness decisions: The role of the cost-effectiveness acceptability curve (CEAC), the cost-effectiveness acceptability frontier (CEAF), and the expected value of perfection information (EVPI). *Value in Health* **11,** 886–897. ISSN: 15244733. `http://dx.doi.org/10.1111/j.1524-4733.2008.00358.x` (2008).

29. Sutherland, C. S. & Tediosi, F. Is the elimination of sleeping sickness' affordable? Who will pay the price? Assessing the financial burden for the elimination of human African trypanosomiasis Trypanosoma brucei gambiense in sub-Saharan Africa. *BMJ Global Health* **4,** 1–11. ISSN: 20597908 (2019).

30. Courtin, F. *et al.* Reducing human-tsetse contact significantly enhances the efficacy of sleeping sickness active screening campaigns: A promising result in the context of elimination. *PLOS Neglected Tropical Diseases* **9,** 1–12. ISSN: 19352735 (2015).

31. Bertram, M. Y. *et al.* Cost – effectiveness thresholds: pros and cons Use and misuse of thresholds. *Bull World Health Organization* **94,** 925–930 (2016).

32. Marseille, E., Larson, B., Kazi, D. S., Kahn, J. G. & Rosen, S. Thresholds for the cost–effectiveness of interventions: Alternative approaches. *Bulletin of the World Health Organization* **93,** 118–124. ISSN: 15640604 (2015).

33. Woods, B., Revill, P., Sculpher, M. & Claxton, K. Country-Level Cost-Effectiveness Thresholds: Initial Estimates and the Need for Further Research. *Value in Health* **19,** 929–935. ISSN: 15244733. `http://dx.doi.org/10.1016/j.jval.2016.02.017` (2016).

34. Behrend, M. R. *et al.* Modelling for policy: The five principles of the neglected tropical diseases modelling consortium. *PLoS Neglected Tropical Diseases* **14,** 1–17. ISSN: 19352735 (2020).

35. Briggs, A. H., Goeree, R., Blackhouse, G. & O'Brien, B. J. Probabilistic Analysis of Cost-Effectiveness Models: Choosing between Treatment Strategies for Gastroesophageal Reflux Disease. *Medical Decision Making* **22,** 290–308. ISSN: 0272-989X. `http://mdm.sagepub.com/cgi/doi/10.1177/0272989X0202200408` (Aug. 2002).

36. Cook, J. D. *Determining distribution parameters from quantiles* Jan. 2010. https://www.johndcook.com/quantiles_parameters.pdf.

37. Joseph, L. & Bélisle, P. *beta.parms.from.quantiles: [R] Computing Beta distribution parameters* 2017. http://www.medicine.mcgill.ca/epidemiology/joseph/pbelisle/BetaParmsFromQuantiles.html.

38. OCHA Office for the Coordination of Humanitarian Affairs. *Journees Nationales de Vaccination (JNV) activities de vaccination supplementaire, RDC* https://data.humdata.org/dataset/rdc-statistiques-des-populations.

39. Mitashi, P. *et al.* Human African Trypanosomiasis Diagnosis in First-Line Health Services of Endemic Countries, a Systematic Review. *PLOS Neglected Tropical Diseases* **6.** ISSN: 19352727 (2012).

40. Jamonneau, V. *et al.* Accuracy of Individual Rapid Tests for Serodiagnosis of Gambiense Sleeping Sickness in West Africa. *PLOS Neglected Tropical Diseases* **9,** 1–9. ISSN: 19352735 (2015).

41. Eperon, G., Schmid, C., Loutan, L. & Chappuis, F. Clinical presentation and treatment outcome of sleeping sickness in Sudanese pre-school children. *Acta Tropica* **101,** 31–39. ISSN: 0001706X. https://linkinghub.elsevier.com/retrieve/pii/S0001706X06002439 (Jan. 2006).

42. Schmid, C. *et al.* In-Hospital Safety in Field Conditions of Nifurtimox Eflornithine Combination Therapy (NECT) for T. b. gambiense Sleeping Sickness. *PLOS Neglected Tropical Diseases* **6.** ISSN: 19352727 (2012).

43. Priotto, G. *et al.* Three Drug Combinations for Late-Stage Trypanosoma brucei gambiense Sleeping Sickness: A Randomized Clinical Trial in Uganda. *PLOS Clinical Trials* **1,** e39. ISSN: 1555-5887 (2006).

44. Priotto, G. *et al.* Nifurtimox-Eflornithine Combination Therapy for Second-Stage Trypanosoma brucei gambiense Sleeping Sickness: A Randomized Clinical Trial in Congo. *Clinical Infectious Diseases* **45,** 1435–1442. ISSN: 1058-4838 (2007).

45. Checchi, F. *et al.* Nifurtimox plus eflornithine for late-stage sleeping sickness in Uganda: A case series. *PLOS Neglected Tropical Diseases* **1,** 1–6 (2007).

46. Priotto, G. *et al.* Nifurtimox-eflornithine combination therapy for second-stage African Trypanosoma brucei gambiense trypanosomiasis: a multicentre, randomised, phase III, non-inferiority trial. *The Lancet* **374,** 56–64. ISSN: 01406736. http://dx.doi.org/10.1016/S0140-6736(09)61117-X (2009).

47. Ngoyi, D. M. *et al.* How to Shorten Patient Follow-Up after Treatment for Trypanosoma brucei gambiense Sleeping Sickness. *The Journal of Infectious Diseases* **201,** 453–463. ISSN: 0022-1899 (2010).

48. Burri, C. *et al.* Efficacy, Safety, and Dose of Pafuramidine, a New Oral Drug for Treatment of First Stage Sleeping Sickness, in a Phase 2a Clinical Study and Phase 2b Randomized Clinical Studies. *PLOS Neglected Tropical Diseases* **10,** 1–18. ISSN: 19352735 (2016).

49. Pohlig, G. *et al.* Efficacy and Safety of Pafuramidine versus Pentamidine Maleate for Treatment of First Stage Sleeping Sickness in a Randomized, Comparator-Controlled, International Phase 3 Clinical Trial. *PLOS Neglected Tropical Diseases* **10,** 1–17. ISSN: 19352735 (2016).

50. Kansiime, F. *et al.* A multicentre, randomised, non-inferiority clinical trial comparing a nifurtimox-eflornithine combination to standard eflornithine monotherapy for late stage Trypanosoma brucei gambiense human African trypanosomiasis in Uganda. *Parasites and Vectors* **11,** 1–11. ISSN: 17563305 (2018).

51. Mesu, V. K. B. K. *et al.* Oral fexinidazole for late-stage African Trypanosoma brucei gambiense trypanosomiasis: a pivotal multicentre, randomised, non-inferiority trial. *The Lancet* **391,** 144–154. ISSN: 1474547X (2018).

52. Balasegaram, M., Harris, S., Checchi, F., Hamel, C. & Karunakara, U. Treatment outcomes and risk factors for relapse in patients with early-stage human African trypanosomiasis (HAT) in the Republic of the Congo. *Bulletin of the World Health Organization* **84,** 777–782. ISSN: 00429686 (2006).

53. Hasker, E. *et al.* Human African Trypanosomiasis in the Democratic Republic of the Congo: A Looming Emergency? *PLOS Neglected Tropical Diseases* **6,** e1950 (2012).

54. Alirol, E. *et al.* Nifurtimox-eflornithine combination therapy for second-stage gambiense human African trypanosomiasis: Médecins Sans Frontières experience in the Democratic Republic of the Congo. *Clinical Infectious Diseases* **56,** 195–203. ISSN: 10584838 (2013).

55. Bastide, S., Priotto, G., Ecochard, R. & Etard, J. F. Effectiveness of short vs. long treatment schedules with pentamidine in first-stage HAT: a large field cohort study. *Tropical Medicine & International Health* **16,** 68–69. https://onlinelibrary.wiley.com/toc/13653156/2011/16/s1 (2011).

56. Checchi, F., Filipe, J. A., Haydon, D. T., Chandramohan, D. & Chappuis, F. Estimates of the duration of the early and late stage of gambiense sleeping sickness. *BMC Infectious Diseases* **8,** 1–10. ISSN: 14712334 (2008).

57. Checchi, F., Funk, S., Chandramohan, D., Haydon, D. T. & Chappuis, F. Updated estimate of the duration of the meningo-encephalitic stage in gambiense human African trypanosomiasis. *BMC Research Notes* **8,** 8–10. ISSN: 17560500 (2015).

58. Checchi, F., Funk, S., Chandramohan, D., Chappuis, F. & Haydon, D. T. The impact of passive case detection on the transmission dynamics of gambiense Human African Trypanosomiasis. *PLOS Neglected Tropical Diseases* **12,** 1–17. ISSN: 19352735 (2018).

59. Hackett, F., Berrang Ford, L., Fèvre, E. & Simarro, P. Incorporating Scale Dependence in Disease Burden Estimates: The Case of Human African Trypanosomiasis in Uganda. *PLOS Neglected Tropical Diseases* **8.** ISSN: 19352735 (2014).

60. Sutherland, C. S., Stone, C. M., Steinmann, P., Tanner, M. & Tediosi, F. Seeing beyond 2020: an economic evaluation of contemporary and emerging strategies for elimination of Trypanosoma brucei gambiense. *The Lancet Global Health* **5,** e69–e79. ISSN: 2214109X. http://dx.doi.org/10.1016/S2214-109X(16)30237-6%20https://linkinghub.elsevier.com/retrieve/pii/S2214109X16302376 (Jan. 2017).

61. Keating, J., Yukich, J. O., Sutherland, C. S., Woods, G. & Tediosi, F. Human African trypanosomiasis prevention, treatment and control costs: A systematic review. *Acta Tropica* **150,** 4–13. ISSN: 0001706X. http://dx.doi.org/10.1016/j.actatropica.2015.06.003%20https://linkinghub.elsevier.com/retrieve/pii/S0001706X15300267 (Oct. 2015).

62. Lutumba, P. *et al.* Efficience de differentes strategies de detection de la Trypanosomiase Humaine Africaine aT. b. gambiense. *Tropical Medicine and International Health* **10,** 347–356. ISSN: 1360-2276. http://doi.wiley.com/10.1111/j.1365-3156.2005.01391.x (Apr. 2005).

63. Irurzun-Lopez, M. *et al.* The actual and potential costs of meningitis surveillance in the African meningitis belt: Results from Chad and Niger. *Vaccine* **34,** 1133–1138. ISSN: 18732518. http://dx.doi.org/10.1016/j.vaccine.2015.10.045 (2016).

64. Bertram, M. Y. *et al.* Disease control programme support costs: an update of WHO-CHOICE methodology, price databases and quantity assumptions. *Cost Effectiveness and Resource Allocation* **15,** 21. ISSN: 1478-7547. http://resource-allocation.biomedcentral.com/articles/10.1186/s12962-017-0083-6 (Dec. 2017).

65. Stenberg, K., Lauer, J. A., Gkountouras, G., Fitzpatrick, C. & Stanciole, A. Econometric estimation of WHO-CHOICE country-specific costs for inpatient and outpatient health service delivery. *Cost Effectiveness and Resource Allocation* **16,** 1–15. ISSN: 14787547. https://doi.org/10.1186/s12962-018-0095-x (2018).

66. Laokri, S., Soelaeman, R. & Hotchkiss, D. R. Assessing out-of-pocket expenditures for primary health care: How responsive is the Democratic Republic of Congo health system to providing financial risk protection? *BMC Health Services Research* **18,** 1–19. ISSN: 14726963 (2018).

67. Simarro, P. P., Diarra, A., Ruiz Postigo, J. A., Franco, J. R. & Jannin, J. G. The human African trypanosomiasis control and surveillance programme of the World Health Organization 2000-2009: the way forward. eng. *PLOS neglected tropical diseases* **5,** e1007. ISSN: 1935-2735 (Electronic) (Feb. 2011).
