# Supplementary material for: Cost-effectiveness of sleeping sickness elimination campaigns in five settings of the Democratic Republic of Congo
Source: Nat Commun. 2022 Feb 25;13:1051. doi: 10.1038/s41467-022-28598-w (PMC8881616; doi:10.1038/s41467-022-28598-w)
Supplement: Supplementary file 3 — Reporting Summary [file 41467_2022_28598_MOESM3_ESM.pdf]

nature research

Corresponding author(s): Marina Antillon
Last updated by author(s): 25 Oct 2021

Reporting Summary

Nature Research wishes to improve the reproducibility of the work that we publish. This form provides structure for consistency and transparency in reporting. For further information on Nature Research policies, see our Editorial Policies and the Editorial Policy Checklist.

Statistics

For all statistical analyses, confirm that the following items are present in the figure legend, table legend, main text, or Methods section.

n/a	Confirmed	
☒	☐	The exact sample size (*n*) for each experimental group/condition, given as a discrete number and unit of measurement
☒	☐	A statement on whether measurements were taken from distinct samples or whether the same sample was measured repeatedly
☒	☐	The statistical test(s) used AND whether they are one- or two-sided<br>*Only common tests should be described solely by name; describe more complex techniques in the Methods section.*
☒	☐	A description of all covariates tested
☒	☐	A description of any assumptions or corrections, such as tests of normality and adjustment for multiple comparisons
☐	☒	A full description of the statistical parameters including central tendency (e.g. means) or other basic estimates (e.g. regression coefficient) AND variation (e.g. standard deviation) or associated estimates of uncertainty (e.g. confidence intervals)
☒	☐	For null hypothesis testing, the test statistic (e.g. *F*, *t*, *r*) with confidence intervals, effect sizes, degrees of freedom and *P* value noted<br>*Give P values as exact values whenever suitable.*
☐	☒	For Bayesian analysis, information on the choice of priors and Markov chain Monte Carlo settings
☒	☐	For hierarchical and complex designs, identification of the appropriate level for tests and full reporting of outcomes
☒	☐	Estimates of effect sizes (e.g. Cohen's *d*, Pearson's *r*), indicating how they were calculated

Our web collection on statistics for biologists contains articles on many of the points above.

Software and code

Policy information about availability of computer code

Data collection	We were not responsible for data collection. The basic inputs to the model were projections from a simulation study and values from the literature.
Data analysis	Epidemiological model fitting (including the MCMC algorithm) was run on Matlab 2018b simulations were run on Matlab 2018b and the economic analyses were run using R 3.6.3. All the code are available in https://osf.io/xbwte/.

For manuscripts utilizing custom algorithms or software that are central to the research but not yet described in published literature, software must be made available to editors and reviewers. We strongly encourage code deposition in a community repository (e.g. GitHub). See the Nature Research guidelines for submitting code & software for further information.

Data

Policy information about availability of data

All manuscripts must include a data availability statement. This statement should provide the following information, where applicable:

- Accession codes, unique identifiers, or web links for publicly available datasets
- A list of figures that have associated raw data
- A description of any restrictions on data availability

Information about the WHO HAT Atlas data used for fitting is described in Crump et al. [17] and screening data from the five health zones in this study were used to inform future potential screening coverage and were obtained through the WHO HAT Atlas. Data cannot be shared publicly because they were aggregated from the World Health Organisation's HAT Atlas which is under the stewardship of the WHO; our data-sharing agreement does not allow us to share that data. WHO HAT Atlas data include identifiable data. Data are available from the WHO (contact neglected.diseases@who.int or visit https://www.who.int/trypanosomiasis_african/country/foci_AFRO/en/) for researchers who meet the criteria for access to confidential data, including secure computational facilities and an existing relationship to the national sleeping control program of the DRC. Time-frame for response would depend on the WHOs timelines and workloads. Clinical outcomes and costs

(listed in Table 5) were simulated using estimates from the literature and are described in Section S6. Assumptions and estimates were parameterized according to conventions in the economic evaluation literature [38].

Field-specific reporting

Please select the one below that is the best fit for your research. If you are not sure, read the appropriate sections before making your selection.

☒ Life sciences ☐ Behavioural & social sciences ☐ Ecological, evolutionary & environmental sciences

For a reference copy of the document with all sections, see nature.com/documents/nr-reporting-summary-flat.pdf

Life sciences study design

All studies must disclose on these points even when the disclosure is negative.

Sample size	As this is a secondary analysis and simulation study parameterized by information from the literature and administrative case data, there is no sample size.
Data exclusions	As this is a simulation study, there were no data exclusions.
Replication	As this is a simulation study, there was no replicability of experiments, but monte carlo samples were designated to be large enough to yield stable results.
Randomization	As this is a simulation study, there is no randomization of patients, etc.
Blinding	As this is a secondary analysis and simulation study and not a trial, blinding is not relevant.

Reporting for specific materials, systems and methods

We require information from authors about some types of materials, experimental systems and methods used in many studies. Here, indicate whether each material, system or method listed is relevant to your study. If you are not sure if a list item applies to your research, read the appropriate section before selecting a response.

Materials & experimental systems

n/a	Involved in the study	
☒	☐	Antibodies
☒	☐	Eukaryotic cell lines
☒	☐	Palaeontology and archaeology
☒	☐	Animals and other organisms
☒	☐	Human research participants
☒	☐	Clinical data
☐	☒	Dual use research of concern

Methods

n/a	Involved in the study	
☒	☐	ChIP-seq
☒	☐	Flow cytometry
☒	☐	MRI-based neuroimaging

Dual use research of concern

Policy information about dual use research of concern

Hazards

Could the accidental, deliberate or reckless misuse of agents or technologies generated in the work, or the application of information presented in the manuscript, pose a threat to:

No	Yes	
☒	☐	Public health
☒	☐	National security
☒	☐	Crops and/or livestock
☒	☐	Ecosystems
☒	☐	Any other significant area

Experiments of concern

Does the work involve any of these experiments of concern:

No	Yes	
☒	☐	Demonstrate how to render a vaccine ineffective
☒	☐	Confer resistance to therapeutically useful antibiotics or antiviral agents
☒	☐	Enhance the virulence of a pathogen or render a nonpathogen virulent
☒	☐	Increase transmissibility of a pathogen
☒	☐	Alter the host range of a pathogen
☒	☐	Enable evasion of diagnostic/detection modalities
☒	☐	Enable the weaponization of a biological agent or toxin
☒	☐	Any other potentially harmful combination of experiments and agents
